# Intriguing Reactivity of a 1,2‐Dihydrodialumane Towards Organic Azides – From a Terminal Diazido–Dialumane to *Pendulum‐Clock‐Like* Azide Bridging

**DOI:** 10.1002/anie.202503638

**Published:** 2025-07-04

**Authors:** Xiaobai Wang, Franziska Traeger, Raphael F. Ligorio, Nico Graw, Regine Herbst‐Irmer, Anna Krawczuk, Malte Fischer, Dietmar Stalke

**Affiliations:** ^1^ Institut für Anorganische Chemie Georg‐August‐Universität Göttingen Tammannstraße 4 Göttingen 37077 Germany

**Keywords:** Dialane, Dialumene, Diazido‐dialane, Organoazide

## Abstract

The reactivity of dihydro‐dialane with organic azides is described. Treatment of the hybrid ligand‐based dialane [(DNI{H}Al)_2_] (**I**) (DNI = [3,3‐dimethyl‐2‐2‐methyl‐2‐(2,6‐diisopropylaniline)ethenyl]‐3H‐indolenine) with TMSN_3_ (TMS = trimethylsilyl) at room temperature gives the first diazido‐dialane [(DNI{N_3_}Al)_2_] (**1**). The transformation from here to a more stable aluminium‐tetrazole [DNIAl(NTMS)_2_N_2_] (**2**) is established. The reaction of other RN_3_ gives [(DNI{H}Al)_2_(κ^2^‐N_3_R)] (R = Benzyl in **3** and 1‐Adamantyl in **4**) with the azide in a μ‐bridging position between two aluminium atoms. Using ^1^H NOESY/EXSY NMR spectroscopy, a positional exchange of the two TMS groups (**2**) via rotation of the tetrazole unit is observed. In contrast, compound **3** exhibits a *pendulum‐clock‐like* dynamic, with N_α_ oscillating between the two aluminium atoms in solution. The reaction of dialane **I** with DippN_3_ (Dipp = 2,6‐^i^Pr_2_‐C_6_H_3_) gives the dialuminium amine [{DNI(H)Al}_2_(μ‐NDipp)] (**5**).

## Graphical abstract

The reaction of a dihydro‐dialane supported by a hybrid ligand (DNI) with organic azides yields structurally diverse aluminium species, including the first example of low oxidation state aluminium azide complex, a diazido‐dialane, along with an aluminium‐tetrazole, and azide alumination. Spectroscopic studies reveal dynamic behaviour in solution, featuring a *pendulum‐clock*‐like motion of the benzyl azide and N_α_ oscillation within the complex.

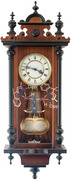

## Introduction

Main‐group chemistry, involving central elements in atypical electronic configurations – such as varied oxidation states – demonstrates great promise in activating strong chemical bonds and advancing catalytic processes.^[^ ^1^, ^2^, ^3^, ^4^, ^5^, ^6^ ^]^ While redox‐type oxidative addition and reductive elimination are typically associated with transition metals, such reactivity has increasingly been observed in main‐group systems. Aluminium, the most abundant metal in the Earth's crust, exemplifies this potential. In the solid state, trivalent aluminium (Al^3+^) plays a key role in materials like zeolites, aluminas, and oxide frameworks, where it enables catalysis, ion exchange, and gas separation.^[^ ^7^, ^8^, ^9^, ^10^ ^]^ Aluminium chemistry has recently expanded beyond its conventional +III state, with growing interest in +I oxidation state species.^[^ ^11^, ^12^, ^13^, ^14^, ^15^ ^]^ These include neutral aluminylenes, first discovered in 1991,^[^ ^16^ ^]^ and aluminyl anions established in 2018,^[^ ^17^ ^]^ both of which, along with their successors and other low‐valent aluminium derivatives, have emerged as true powerhouses in selectively activating and transforming even the thermodynamically strongest bonds.^[^ ^13^, ^18^, ^19^, ^20^, ^21^, ^22^, ^23^, ^24^ ^]^

**Scheme 1:**
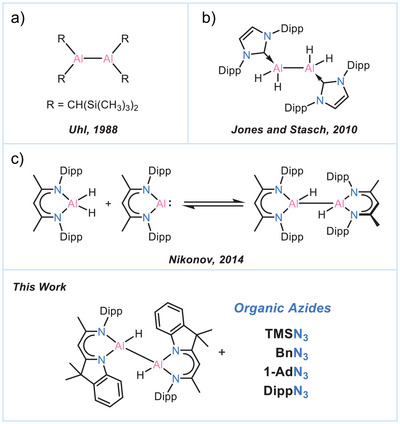
a–c) Milestones in the chemistry of dialumanes.

Neutral complexes of the type R_2_AlAlR_2_, which possess Al—Al single bonds [commonly referred to as dialumanes or dialane(4)], represent another remarkable class of low‐valent aluminium complexes where both aluminium atoms are in the +II oxidation state. Isolating and stabilizing these complexes is rather challenging due to the small Al─Al bond energy, the electropositive nature of Al, and the inherent Lewis acidity of these species with formal six valence electrons.^[^ ^13^ ^]^ Indeed, the isolation of these complexes was predicted to be inaccessible based on calculations for Al_2_H_4_, stating that complexes of this type will generally decompose through disproportionation.^[^ ^25^ ^]^ This assumption was debunked with the first dialumane synthesis of [{(Me_3_Si)_2_CH}_2_Al]_2_, which was structurally characterized. Its isolation was enabled by the support of kinetically stabilizing and sterically demanding substituents (Scheme 1a).^[^ ^26^ ^]^ Since then, this field has rapidly expanded.^[^ ^13^, ^27^, ^28^, ^29^, ^30^ ^]^ Consequently, various 1,2‐dihalodialumanes,^[^ ^31^, ^32^, ^33^, ^34^ ^]^ first examples of unsymmetrically functionalized dialumanes,^[^ ^35^, ^36^ ^]^ and 1,2‐dihydrodialanes have been synthesized and thoroughly characterized in recent years.^[^ ^13^, ^22^, ^37^, ^38^, ^39^, ^40^ ^]^ Remarkably, even the parent 1,2‐dihydrodialane Al_2_H_4_ can be tamed through stabilization by *N*‐heterocyclic carbenes (Scheme 1b).^[^ ^37^ ^]^ Despite these significant developments in the field, the reactivity of dialumanes remains relatively underexplored due to their propensity for disproportionation into corresponding Al(I) and Al(III) complexes (Scheme 1c). This includes 1,2‐dihydrodialanes, which tend to disproportionate into respective dihydroalanes and aluminylene species. These reactions were found to be in equilibrium, suggesting facile reductive elimination at the Al─H bonds and raising expectations for catalytic applications of these complexes in the foreseeable future.^[^ ^4^, ^22^, ^37^, ^39^ ^]^

We have recently reported the straightforward synthesis of 1,2‐dihydrodialane [(DNI{H}Al)_2_] (**I**), supported by a β‐diketiminato‐indole‐hybrid ligand (DNI),^[^ ^41^ ^]^ and revealed its propensity to cleave the C_Ar_(sp^2^)─H bond.^[^ ^42^ ^]^ Given the scarce reports on the selective reactivity of 1,2‐dihydrodialanes (*vide supra*), we herein present the diverse reactivity of **I** towards a series of organic azides. Sterically encumbered azides have ever since been in the focus of low‐valent aluminium chemistry.^[^ ^43^, ^44^, ^45^ ^]^ In this paper we present the wide product range from a unique and highly reactive terminal diazido‐dialane to *pendulum‐clock‐like* bridged aluminium azide species. Accordingly, this study represents the first investigation of 1,2‐dihydrodialanes' reactivity towards organic azides. Our findings highlight new reaction pathways that are distinctly different from those observed in the limited reports on the reactions of dihalo‐ and diamido‐substituted dialumanes.^[^ ^34^, ^46^ ^]^

## Results and Discussion

**Scheme 2:**
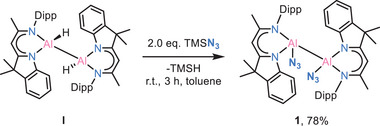
Synthesis of complex 1.

The reaction of dialane [(DNI{H}Al)_2_] (**I**) with 2.0 eq. TMSN_3_ in toluene at room temperature gives the diazido‐dialane complex [(DNI{N_3_}Al)_2_] (**1**) with a good yield of 78%. Rapid crystal formation was observed upon cooling the solution to −30°C (Scheme 2).

**Figure 1:**
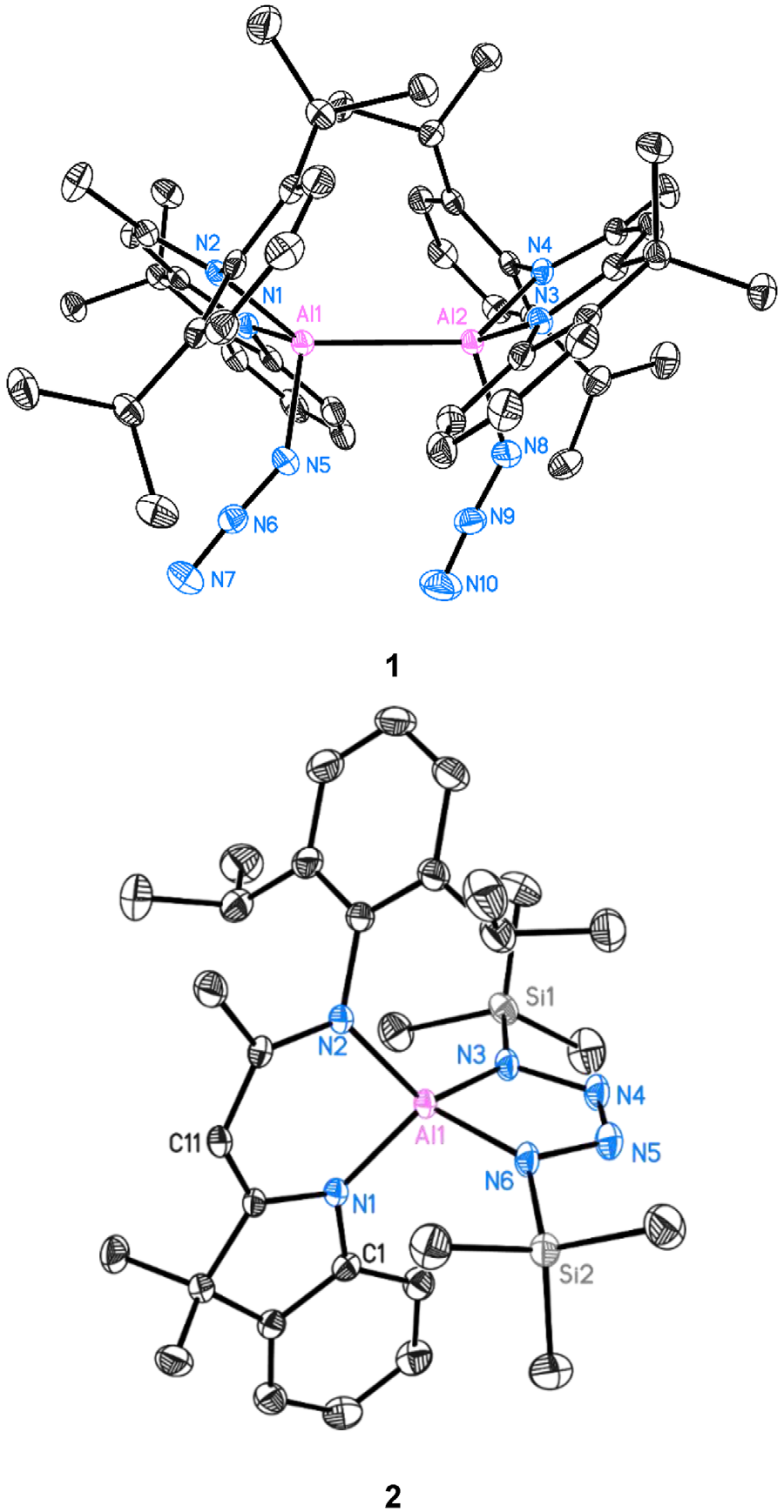
Molecular structure of [(DNI{N_3_}Al)_2_] (**1**) and [DNIAl(NTMS)_2_N_2_] (**2**). Anisotropic displacement parameters are depicted at the 50% probability level. All ligand‐based hydrogen atoms are omitted. Selected bond lengths (Å) and angles (°): For **1**: Al1─Al2 2.6008(12), Al1─N1 1.9043(16), Al1─N2 1.9310(16), Al1─N5 1.8669(18), N5─N6 1.203(2), N6─N7 1.150(2), Al2─N3 1.9009(16), Al2─N4 1.9225(17), Al2─N8 1.8686(18), N8─N9 1.210(2), N9─N10 1.142(2); N1─Al1─N2 94.25(7), Al2─Al1─N5 97.21(6), Al1─N5─N6 140.59(14), N5─N6─N7 175.91(19), N3─Al2─N4 94.74(7), Al1─Al2─N8 106.60(6), Al2─N8─N9 135.60(14), N8─N9─N10 176.2(2). For **2**: Al1─N1 1.8708(13), Al1─N2 1.8812(13), Al1─N3 1.8260(13), Al1─N6 1.8312(13), N3─N4 1.4128(17), N4─N5 1.2671(19), N5─N6 1.4010(17), Si(1)─N3 1.7417(13), Si(2)─N6 1.7414(14); N1─Al1─N2 95.99(6), N1─Al1─N6 110.76(6), N2─Al1─N3 121.87(6), N1─Al1─N3 121.20(6), N2─Al1─N6 121.80(6), N3─Al1─N6 87.04(6), N3─N4─N5 116.59(12), N4─N5─N6 116.21(12), Al1─N6─N5 110.32(10).

Complex **1** crystallizes in the triclinic space group *P* $\bar{1}$ with two toluene molecules in the asymmetric unit. The molecular structure reveals the maintenance of the Al─Al bond with each Al(II) atom coordinated by an azide group (Figure 1). The Al─Al bond length of 2.6008(12) Å remains almost identical to that of the dialane [(DNI{H}Al)_2_] (**I**) starting material (2.6007(6) Å). The Al─N bond lengths to the azide group are 1.8669(18) Å (Al1─N5) and 1.8686(18) Å (Al2─N8), respectively. Accordingly, they are slightly longer than in the previously reported Al(III)−N_3_ complex, for example, [(^Dipp^BDIAl{N_3_}N{μ‐Si(N_3_)*t*Bu})_2_] by Roesky^[^ ^47^ ^]^ and [Ter*^i^* ^Pr8^Al{N_3_}{N(SiMe_3_)_2_}] by Power.^[^ ^44^ ^]^ These differences can be attributed to the slightly smaller radius of Al(III) compared to Al(II). In **1** the two azide groups point in the same direction. Their structural parameters are N5─N6 1.203(2) Å, N6─N7 1.150(2) Å, N8─N9 1.210(2), N9─N10 1.142(2), and they adopt a slightly nonlinear arrangement of N_α_─N_β_─N_γ_ with 175.91(19)° for N5─N6─N7 and 176.2(2)° for N8─N9─N10, respectively.

The dislocation of the two Al atoms from their C_3_N_2_ mean planes are minute with *d*(Al···C_3_N_2_) = 0.039(2) Å for Al1 and 0.048(2) Å for Al2. This is consistent with the slight folding angle of the ligand C_3_N_2_ mean plane [N1─C10─C11/N2─C12─C11: 4.1(4)°, N3─C35─C36/N4─C37─C36: 5.3(3)°].

Density functional theory (DFT) calculations at the B3LYP/6–31+G(d) level of theory were carried out using crystallographic coordinates to investigate the bonding in **1**. The calculated energies of the highest occupied molecular orbital (HOMO) and lowest unoccupied molecular orbital (LUMO) are presented in the Supporting Information (see Figure S35). The HOMO, and similarly the HOMO‐2, is primarily localized on the Al─Al σ‐bond, while significant contributions to the molecular orbitals from the azide ligands are noted between HOMO‐3 and HOMO‐5. This highlights the electronic interplay between the aluminium core and the azide groups.

**Figure 2:**
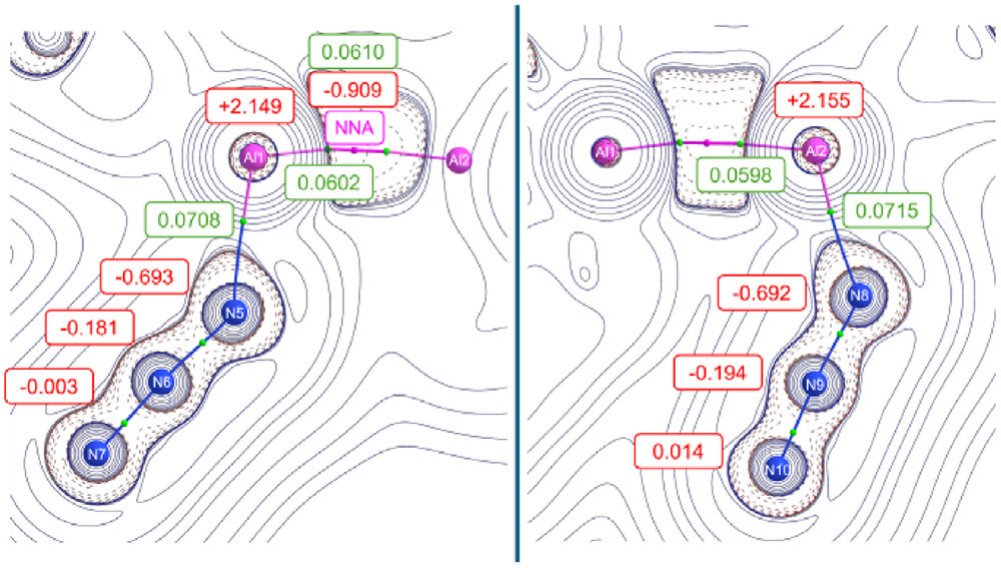
Laplacian operator of the electron density in a plane containing aluminium nuclei and the azide unit in **1**. Contours are drawn in a logarithmic scale: positive as solid blue lines and negative ones as dashed red lines. The non‐nuclear attractor (NNA) is depicted as a magenta dot, AIM charges are given in red boxes for both Al ions and all nitrogen atoms, charge density magnitudes at both bond critical points at green dots are given in green boxes (e^−^/bohr^3^).

The Al─Al interaction is characterized by a non‐nuclear attractor (NNA), a local maximum in the electron density that does not coincide with any nucleus, as first predicted by Gatti and Bader^[^ ^48^ ^]^ later experimentally verified by Platts et al.^[^ ^49^ ^]^ Subsequently, it was observed in our previous work^[^ ^42^ ^]^ and reported by others.^[^ ^50^, ^51^, ^52^, ^53^, ^54^, ^55^ ^]^ QTAIM analysis^[^ ^56^ ^]^ assigns to the NNA an electron density of 0.06 e^−^/bohr^3^ and a charge of −0.909 e^−^, indicating localized electron accumulation in the interatomic region (Figure 2 and Table S8 in SI). This represents the first observation of an NNA in an aluminium azide complex. Additionally, the Wiberg bond index (WBI) of 0.91 further supports a single‐bond framework, highlighting the shared electronic interaction facilitated by the NNA. The Al─N bond lengths to the azide units reflect moderate covalency. The QTAIM analysis reveals slightly elevated charge densities at the Al─N bond critical points (BCPs) compared to other Al─N ligand bonds, consistent with the electron‐withdrawing inductive effect of the azide groups. Notably, natural bond orbital (NBO) calculations (see Figure S36 and Table S10 in the Supporting Information) indicate partial charge transfer from the Al atoms to the azide moieties, highlighting their role as strong electron acceptors. This effect is further supported by an increase in the partial charges on Al1 and Al2 compared to the parent dialane **I** (2.102 and 2.129 e^−^, respectively). The observed Δq increases, Δq(Al1) = +0.047 and Δq(Al2) = +0.026, suggest that the azide groups exert a stronger electron‐withdrawing inductive effect than the hydrogen substituents in dialane **I**.

The azide groups themselves exhibit distinct bonding features. The N─N electron density at the BCPs ranges from 0.465 to 0.553 e^−^/bohr^3^, showcasing partial delocalization, with stronger covalent character of the terminal N─N bonds. Ellipticity values derived from QTAIM indicate a mixed bonding character, with the terminal N─N bonds (Table S8) displaying lower ellipticity compared to the central N─N bond, suggesting a more pronounced π‐contribution in the former, pointing towards triple bond behaviour. The Wiberg bond indices for the azide N─N bonds, averaging to 1.62 for the central bonds and 2.24 for the terminal bonds (see Table S9 in SI), supporting these trends with the terminal bonds indicating stronger multiple‐bond character.

Complex **1** remains stable in the solid‐state for approximately one week at −30°C. However, in solution, it gradually decomposes into unidentified products under ambient conditions, leading to poorly resolved ¹H NMR spectra despite multiple attempts. Both ^1^H and ^13^C NMR spectroscopy indicate the presence of multiple species in solution. Additionally, ^1^H DOSY NMR experiments^[^ ^57^, ^58^, ^59^, ^60^, ^61^ ^]^ further support the decomposition of complex **1** (Figures S2–S4 in SI). Attempts to determine the molecular weight revealed that the decomposed products have lower molecular weights than the parent diazido‐dialane (856.5 gmol^−1^); however, an unambiguous structural assignment was not possible. Notably, the decomposition process accelerates significantly at elevated temperatures.

**Scheme 3:**
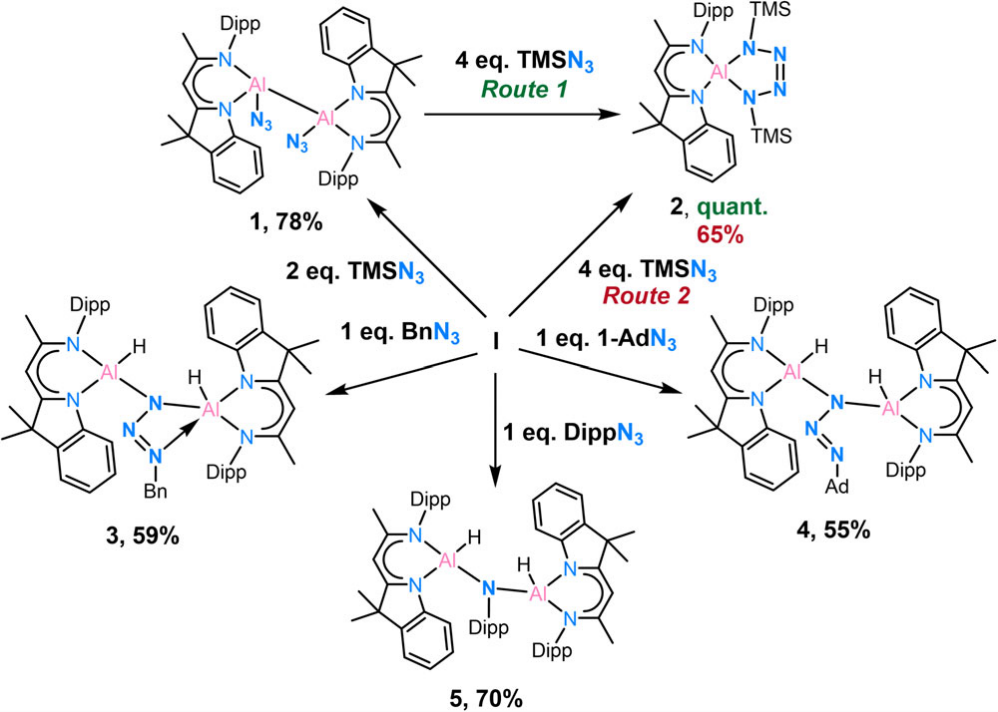
Syntheses of complexes **1**–**5**.

To the best of our knowledge, **1** is the first reported example of a diazido‐dialane, and its reactivity remains unexplored. In here, however, it was serendipitously discovered that the addition of an excess of TMSN_3_ to **1** at −30°C for one week (**Route 1**, Scheme 3) results in the formation of a thermally stable aluminium‐containing tetrazole complex [DNIAl(NTMS)_2_N_2_] (**2**) in quantitative yield. Alternatively, the addition of 4.0 eq. TMSN_3_ to the starting dialane material **I** (**Route 2**, Scheme 3) followed by warming to 70°C for 18 h, consistently yields the identical yellow crystalline solid of **2** with a yield of 65%. It is worthy to note that even when 2.0 eq. of TMSN_3_ were employed, only **2** can be isolated. Its single crystal X‐ray structure is depicted in Figure 1. Complex **2** crystallised in space group *C*2/*c* with one moelcule and a half toluene molecules in the asymmetric unit. The molecular structure reveals a distorted tetrahedral coordination geometry at the aluminium atom. It exhibits notable dislocation of *d*(Al···C_3_N_2_) = 0.5363(17) Å from the ligand's C_3_N_2_ mean plane. The five‐membered tetrazole ring, however, is planar (*d*(Al···(NTMS)_2_N_2_) = 0.012(2) Å). The Al1−N3 (1.8260(13) Å) and Al1−N6 (1.8312(13) Å) distances are similar to those reported for [^Dipp^BDIAl(NTMS)_2_N_2_]^[^ ^62^ ^]^ (*d*(Al−N): 1.8152(15) Å, 1.851(2) Å).

**Figure 3:**
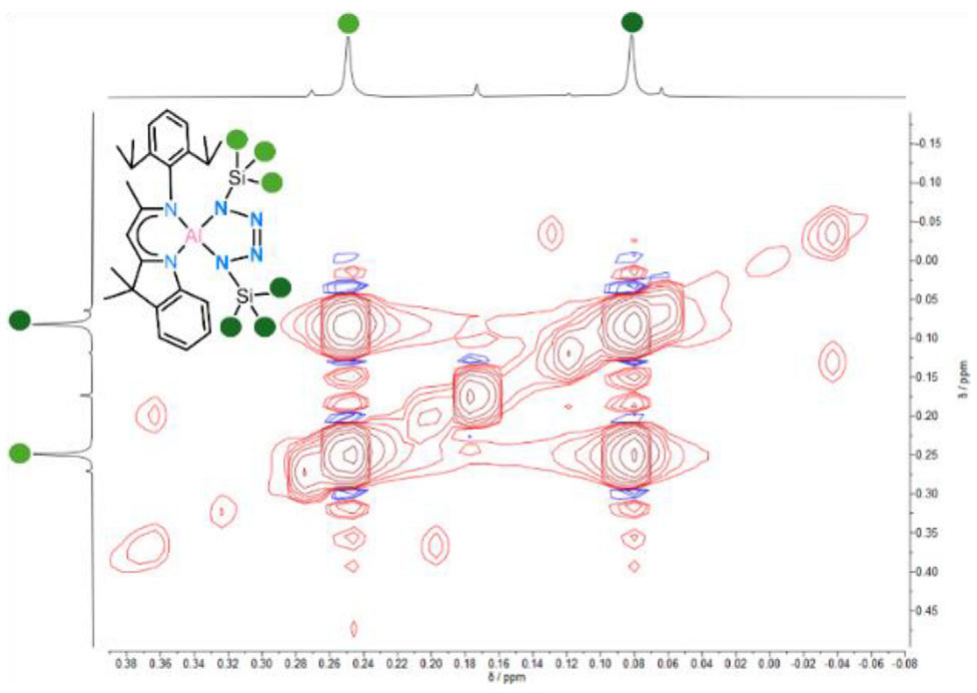
Excerpt from the ^1^H EXSY spectrum of complex **2** showing the TMS methyl groups.

The ^1^H NMR spectroscopic signal of backbone proton H11 (δ = 5.08 ppm) is slightly downfield shifted compared to the starting material **I** (δ = 5.01 ppm). The protons of the trimethylsilyl group display two separate signals at δ = 0.25 and 0.08 ppm. The ^27^Al NMR spectrum shows a single signal at δ = 77.49 ppm while the ^1^H,^29^Si HMBC NMR spectrum shows two different signals at δ = 2.5 and −1.6 ppm, respectively. Especially the ^1^H EXSY NMR spectrum reveals exchange signals between the two TMS methyl groups (Figure 3). This observation suggests dynamic behaviour of the tetrazole moiety in solution, indicating a position change of the two TMS groups via rotation of the tetrazole unit while the ligand geometry is preserved.

**Figure 4:**
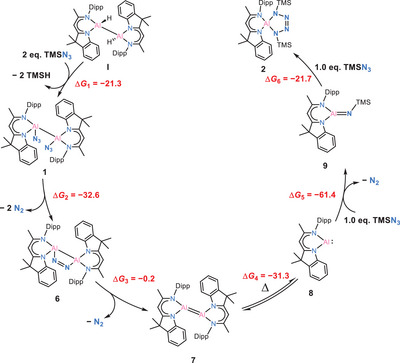
Proposed mechanism for synthesis of complex **2**. The free energy changes for each step are given in kcal mol^−1^.

Even given the existing reports of aluminium‐tetrazoles synthesized by Roesky,^[^ ^47^, ^62^ ^]^ Aldridge,^[^ ^63^ ^]^ and Power,^[^ ^44^ ^]^ the synthesis of aluminium‐tetrazoles via dihydrodialane or diazido‐dialane was previously unknown. Based on the recent and previously reported examples, we propose that the final step in forming the tetrazole complex **2** involves a [2+3] cycloaddition between an aluminium‐imide intermediate [DNIAl═NTMS] (**9**) and an additional azide molecule. The aluminium‐imide intermediate is suggested to be formed through the reaction of [DNIAl:] (**8**) with an azide, accompanied by the release of dinitrogen gas. This leads to the critical point that generating monomeric [DNIAl:] (**8**) is clearly the key step in this reaction. Accordingly, we propose a mechanism to explain this process, shown in the Figure 4. It begins with the azidation of dialane **I** with 2.0 eq. of TMSN_3_, yielding diazido‐dialane **1**. NMR spectroscopy suggests the decomposition of **1** in solution, potentially involving the release of N_2_ gas from the parent diazido‐dialane. This transformation most likely proceeds, similar to Staudinger‐type reaction, via the formation of the Al_2_N_2_ four‐membered ring complex [(DNI)Al(μ‐η¹:η¹‐N_2_)Al(DNI)] (**6**). In this process, the liberation of diatomic nitrogen reduces Al(II), leading to the formation of a transient dialumene species, [(DNI)Al═Al(DNI)] (**7**), accompanied by the release of another equivalent of N_2_. The comparison of experimental conditions in **Route 1** and **Route 2** confirms that this sequence of reactions is endothermic. Notably, the dissociation of dialumene to the monomeric aluminium(I) species has previously been firmly recognized and characterized as an endothermic process.^[^ ^39^, ^46^, ^64^, ^65^ ^]^ Drawing from studies on aluminium‐tetrazole complexes,^[^ ^44^, ^47^, ^62^, ^63^ ^]^ the subsequent reaction pathway involves the formation of an iminodialane intermediate [(DNI)Al═N(TMS)] (**9**), undergoing a [2+3] cycloaddition and ultimately resulting in the formation of a five‐membered aluminium‐tetrazole ring. This sequence rationalizes the observed experimental reactivity and the corresponding products.

To examine the proposed mechanism for the formation of the tetrazole compound **2**, a series of DFT calculations were conducted to evaluate the thermodynamic feasibility of each elementary step (Figure 4 and Supporting Information). The transformation begins with the reaction of dialane **I** [(DNI{H}Al)_2_] with two equivalents of TMSN_3_, forming the diazido‐dialane **1** and releasing two equivalents of TMSH (Δ*G*_1_ = −21.3 kcal mol^−1^). Subsequently, **1** decomposes via extrusion of dinitrogen to form the Al_2_N_2_ four‐membered ring compound **6** (Δ*G*_2_ = −32.6 kcal mol^−1^), a structural motif reminiscent of an intermediate proposed in Staudinger‐type reactivity. Further loss of dinitrogen from **6** affords the dialumene **7** (Δ*G*_3_ = −0.2 kcal mol^−1^), which then dissociates into two monomeric aluminium(I) [DNIAl:] **8** (Δ*G*_4_ = −31.3 kcal mol^−1^). Although dissociation of dialumenes into monomeric Al(I) species is typically considered endergonic due to the associated bond dissociation energy, the process is thermodynamically favourable in this case. This can be rationalized by the high reactivity and pronounced instability of **7**, which likely destabilizes the dimer relative to the monomers in our system. The highly reactive aluminium(I) species **8** then reacts with TMSN_3_ to afford the iminoalane **9**, accompanied by the release of N_2_ (Δ*G*_5_ = −61.4 kcal mol^−1^). A second equivalent of TMSN_3_ converts **9** to the final tetrazole product **2** (Δ*G*_6_ = −21.7 kcal mol^−1^). All individual steps are exergonic, supporting the thermodynamic plausibility of the proposed multistep mechanism.

**Figure 5:**
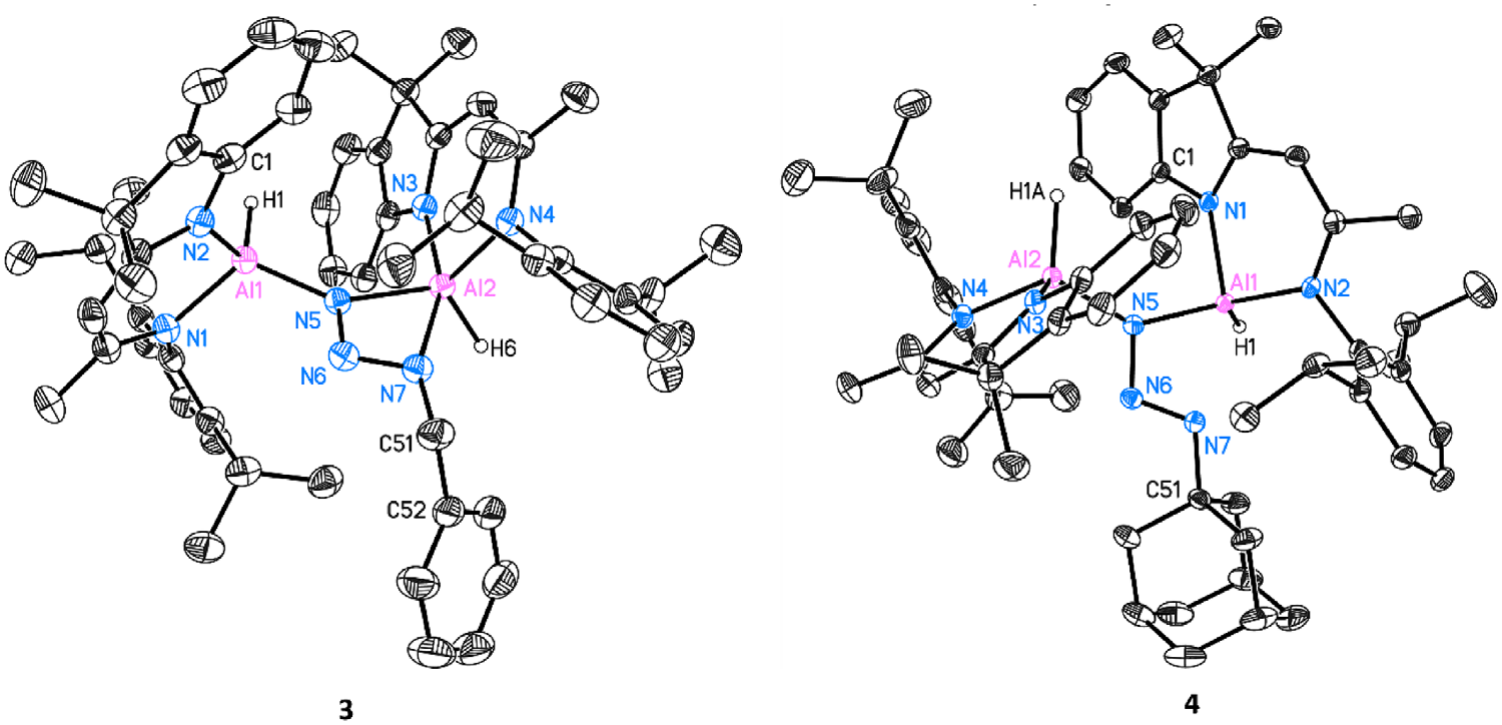
Molecular structure of [{DNI(H)Al}_2_(κ^2^‐N_3_Bn)] (**3**) and [{DNI(H)Al}_2_(μ‐N_3_Ad)] (**4**). The anisotropic displacement parameters are depicted at the 50% probability level. All ligand‐based hydrogen atoms are omitted for clarity. Selected bond lengths (Å) and angles (°): For **3**: Al1─N5 1.8361(14), Al2─N5 1.9074(14), N5─N6 1.3436(19), N6─N7 1.288(2), Al2─N7 2.1633(15), N7─C51 1.465(2), Al1─N1 1.9061(15), Al1─N2 1.8877(14), Al2─N3 1.9492(14), Al2─N4 1.9405(14); Al1─N5─Al2 140.97(8), N5─N6─N7 108.17(13), N6─N7─Al2 89.49(9), N6─N7─C51 114.20(14), N7─C51─C52 113.47(15), N1─Al1─N2 95.52(6), N3─Al2─N4 91.98(6). For **4**: Al1─N1 1.9460(11), Al1─N2 1.9453(11), Al2─N3 1.8829(11), Al2─N4 1.9083(11), Al1─N5 1.8765(11), Al1─N7 2.3347(12) Å, Al2─N5 1.8321(11), N5─N6 1.3565(15), N6─N7 1.2739(15), N7─C51 1.471(5); N1─Al1─N2 91.82(5), N1─Al1─N5 95.89(5), N2─Al1─N5 123.22(5), N3─Al2─N4 94.90(5), N3─Al2─N5 107.31(5), N4─Al2─N5 115.82(5), N5─N6─N7 109.42(10), N6─N7─C51 115.50(15).

The observed reactivity encouraged further investigation with other organic azides. Thus, BnN_3_, 1‐AdN_3_, and DippN_3_ were separately reacted with dialane **I** in 1:1 ratio at room temperature. Complex [(DNI{H}Al)_2_(κ^2^‐N_3_Bn)] (**3**) was obtained in the reaction with BnN_3_ in toluene as yellow crystalline solid in good yield (Scheme 3, 59%). The single crystal X‐ray structure determination unambiguously reveals a bridging organo‐azido group and a five‐coordinated aluminium centre (Al2) exhibiting a κ^2^ planar coordinated geometry [*d*(Al···N_3,azide_) = 0.0077(4) Å] (see Figure 5). The Al─N bond lengths to the five‐coordinated aluminium atom Al2 of *d*(Al2─N3) = 1.9492(14) Å and *d*(Al2─N4) = 1.9405(14) Å are longer than *d*(Al1─N1) = 1.9061(15) Å and *d*(Al1─N2) = 1.8877(14) Å, which is attributable to the higher coordination number. A significantly longer additional δ‐donor bond N7→Al2 is formed at 2.1633(15) Å.

The reaction of dialane I with 1‐AdN_3_ in toluene yielded the crystalline complex [(DNI{H}Al)_2_(μ‐N_3_Ad)] (**4**, 54%, Scheme 3). The molecular structure, as shown in Figure 5 reveals the presence of a bridging azido group. The orientation of N7 towards Al1 results in a much longer Al1···N7 distance of 2.3347(12) Å than in **3**, suggesting an even weaker interaction. The bending angle of N5─N6─N7 (109.42(10)°) is slightly larger than in **3** (108.17(13)°). Additionally, the N─N distances within the azido group [N5─N6 1.3565(15), N6─N7 1.2739(15) Å] indicate less electron delocalization compared to complex **3**.

**Figure 6:**
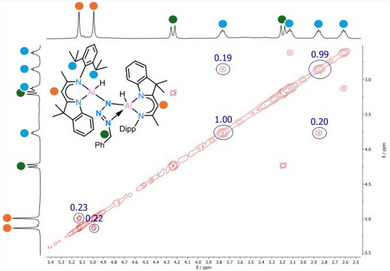
Excerpt from ^1^H EXSY NMR spectrum of complex **3**.

**Scheme 4:**
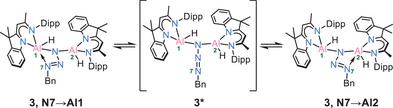
The “N_3_Bn − *pendulum clock*” dynamic equilibrium of the benzyl azide adduct **3** in solution.

In the solid‐state, **3** remains stable for at least one month when stored at −30°C. Similarly, in toluene solution, **3** exhibits a remarkable thermal stability, with neither N_2_ loss nor significant changes in the ¹H NMR spectrum observed, unless heated to 120°C for 1 h. At room temperature additional insights into the dynamic of **3** in solution comes from NMR analysis. Two distinct backbone signals are visible at 5.13 and 4.99 ppm, respectively. The ^1^H‐EXSY spectrum recorded at 298 K (Figure 6) reveals exchange signals involving the two backbone resonances and several other protons, including those of the Bn─C*H_2_* group (highlighted in green). This indicates that N7 is not rigidly coordinated to either of the aluminium atoms (Al1, Al2) but rather undergoes continuous oscillation between them via 3*, with an estimated exchange rate of ∼4 s⁻¹. This dynamic behaviour resembles a *pendulum‐like* motion of the N_3_Bn group, similar to that observed from the tetrazole moiety in **2** (Scheme 4).

Attempts to obtain satisfactory ^1^H, ^13^C, and 2D NMR spectra of **4** at 298 K were unsuccessful, likely due to its instability in solution. Consequently, NMR measurements were attempted at 243 K. However, despite multiple attempts, a satisfactory spectral analysis could not be achieved either. However, DOSY NMR spectroscopy with adamantane as an internal reference in tol‐*d*_8_ at 243 K was instructive. The diffusion coefficients of the selected singlets at δ = 5.35, 5.22, 4.91, 4.80, and 4.70 ppm in the backbone region showed only minimal differences, ranging from 1.646 to 1.665 × 10^−10^ m^2^. This indicates that the species in solution are likely not undergoing significant structural changes or aggregation. However, the molar mass calculated from the diffusion coefficients (*MW_calc_* = 883 g mol^−1^) is lower than the expected molar mass of **4** (*MW_det_* = 952 g mol^−1^). From that, we propose that this outcome may result from the gradual release of nitrogen gas in solution, leading ultimately to the formation of [(DNI{H}Al)_2_(μ‐NAd)]. To facilitate azide decomposition, the solution was heated to 60°C for 1 h, which resulted in a significant darkening of its colour. Unfortunately, attempts to crystallize and isolate the expected dialuminium amine [(DNI{H}Al)_2_(μ‐NAd)] as the product from nitrogen gas liberation were unsuccessful.

Building on this structural information, the ^1^H‐NOESY NMR spectrum of complex **4** shows no clear exchange signals, indicating that the *pendulum‐clock* dynamic observed in **2** and **3** is absent in **4**. This confirms that the bridging azide in complex **4** exists in a relatively “free” state in solution, with the N_α_ atom not directly coordinating to either aluminium atom.

**Figure 7:**
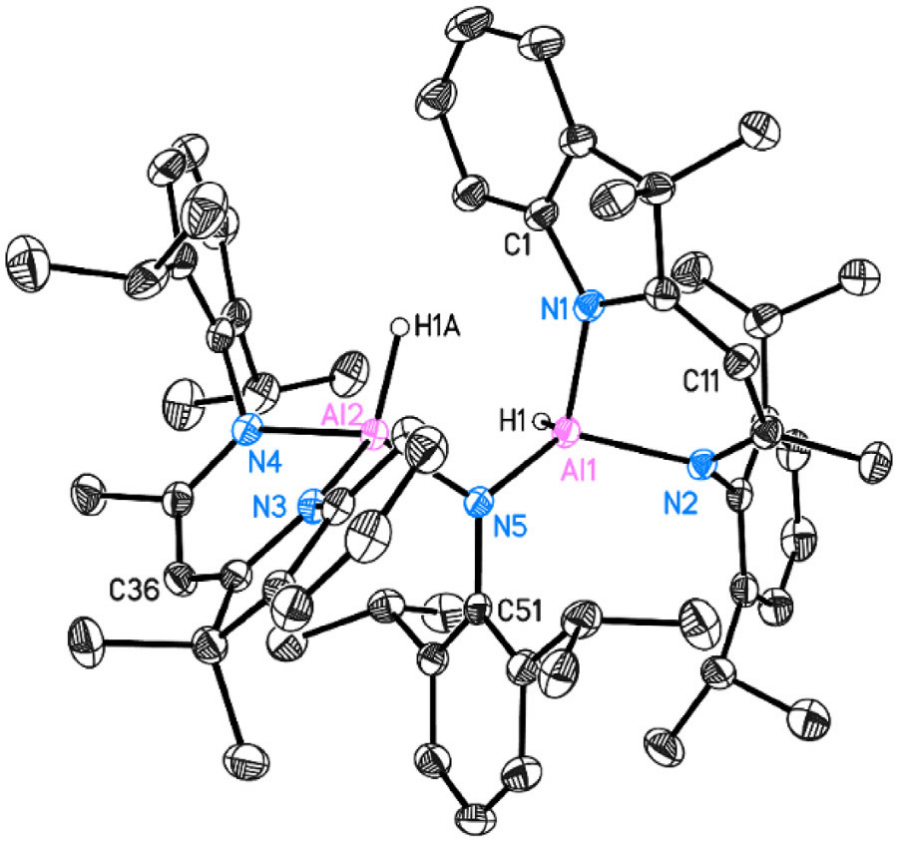
Molecular structure of [{DNI(H)Al}_2_(μ‐NDipp)] (5). The anisotropic displacement parameters are depicted at the 50% probability level. All ligand‐based hydrogen atoms are omitted for clarity. Selected bond lengths (Å) and angles (°): Al1─N1 1.9081(11), Al1─N2 1.9318(11), Al2─N3 1.9035(11), Al2─N4 1.9543(11), Al1─N5 1.8402(11), Al2─N5 1.8295(11), N5─C51 1.4363(16); N1─Al1─N2 92.79(5), N3─Al2─N4 91.75(5), N1─Al1─N5 109.09(5), N2─Al1─N5 120.46(5), N3─Al2─N5 112.22(5), N4─Al2─N5 120.29(5), Al1─N5─Al5 118.27(6), Al1─N5─C51 121.68(8), Al2─N5─C51 119.13(8).

The reaction between dialane **I** and the more sterically demanding DippN_3_ azide was anticipated to form the κ^2^‐ or μ‐N_3_R (R = Dipp) structure like in **3** and **4**. Unexpectedly, a significant release of gas was observed during the reaction, enabling the formation of a dialuminium amine product impossible to isolate from the reaction with Ad─N_3_. Crystals suitable for SC‐XRD experiments are obtained by leaving the benzene solution at room temperature for one day. The structure confirms the N_2_ loss during the reaction, resulting in a N─Dipp bridging moiety between the two aluminium atoms (Figure 7).

**5** crystallises in the monoclinic space group *P*2_1_/*n* with one molecule and disordered lattice solvent in the asymmetric unit. The structure shows a significant butterfly folding of the ligand's C_3_N_2_ mean planes (N1─C10─C11/N2─C12─C11: 21.27(12), N3─C35─C36/N4─C37─C36: 18.5(2)°), resulting in a remarkable dislocation of aluminium from the ligand's C_3_N_2_ mean plane [*d*(Al1···C_3_N_2_) = 0.7668(14) Å; *d*(Al2···C_3_N_2_) = 0.7150(14) Å].

The ^1^H NMR spectrum shows two singlet signals at δ = 5.33 and 4.82 ppm, respectively, assigned to the ligand's backbone. (See Supporting Information) The Dipp─C*H* protons at both ligands show four signals at δ = 3.89, 2.59, 2.45, and 2.23 ppm, respectively. The ^1^H‐NOESY NMR spectrum of **5** shows cross‐peaks between the Dipp─C*H* protons of two different groups: one belonging to the bridging Dipp ligand and the other attached to the DNI ligand. This observation is corroborated by the solid‐state structure, which reveals a spatial distance of 2.113 Å between H45 and H57, close enough to generate cross‐peaks in the ^1^H‐NOESY spectrum. Consequently, the protons of the bridging Dipp group are unambiguously assigned to two distinct signals at δ = 4.31 and 3.60 ppm. These findings confirm that the molecule retains the solid‐state structure in solution.

## Conclusion

In summary, we have investigated the reactivity of hybrid‐ligand based (DNI) dialane [(DNI{N_3_}Al)_2_] (**I**) with different organo‐azides, containing various bulky substitutes. The first low oxidation state aluminium azide complex, diazido‐dialane [(DNI{N_3_}Al)_2_] (**1**), was successfully isolated and characterized. Notably, complex **1** was identified as an isolable intermediate in the reaction of dialane with TMSN_3_ at elevated temperature, ultimately yielding the stable tetrazole complex [DNIAl(NTMS)_2_N_2_] (**2**), most probably via (DNI)Al═Al(DNI) and (DNI)Al═N(TMS). The reaction of dialane with BnN_3_ and 1‐AdN_3_ both led to the formation of bridged azido‐dialane complex **3** and **4** ([(DNI{H}Al)_2_(κ^2^‐N_3_R)] (R = Bn in **3** and 1‐Ad in **4**). Detailed ^1^H EXSY NMR spectroscopy reveals for **2** a positional exchange of the two TMS groups through the rotation of the tetrazole unit and **3** exhibits *pendulum‐clock‐like* dynamics in solution, with exchange rate successfully determined to be 4 s^−1^. On the other hand, the reaction of dialane with DippN_3_ yielded the dialuminium amine product [{DNI(H)Al}_2_(μ‐NDipp)] (**5**). Hence, this work expands the understanding of the reactivity of dialane with organic azides considerably.

## Experimental

### General Procedures

All manipulations that require processing under inert conditions were carried out under an argon atmosphere using standard Schlenk techniques or an MBraun Glovebox. Toluene was distilled from sodium. Toluene‐*d*_8_, benzene and benzene‐*d*_6_ were distilled from potassium. *n*‐Hexane was distilled from Na/K alloy. All solvents were routinely degassed three times using the freeze‐pump‐thaw method and stored in a glove box over molecular sieve. The discussed dialane was synthesised according to a literature procedure.^[^ ^42^ ^]^ TMSN_3_ and 1‐AdN_3_ were purchased from Sigma Aldridge. BnN_3_ was purchased from ABCR. DippN_3_ was synthesised according to a literature procedure.^[^ ^66^ ^]^ 1‐AdN_3_ was purified by stirring a toluene solution of 1‐AdN_3_ over molecular sieves, followed by filtration and vacuum drying. Elemental analysis was performed on an Elementar Vario EL3 by the Analytisches Labor, Institut für Anorganische Chemie, Universität Göttingen. Mass spectra were measured by the Zentrale Analytik within the Faculty of Chemistry, Universität Göttingen applying a Liquid Injection Field Desorption Ionisation‐technique on a JEOL accuTOF instrument with an inert‐sample application setup under argon atmosphere. Fluorescence analyses were conducted using a FluoroMax‐4 spectrometer from HORIBA Jobin Yvon. The quantum yield was determined with the quanta‐φ integrating sphere. At the host computer, the software FluorEssence v3.0 and OriginPro 8.5 G were used to record the information of the fluorescence spectra and analyse the data in the graph. The ^1^H, ^13^C, ^15^N, and 2D NMR spectroscopic data were recorded on a Bruker Avance III 300 MHz, a Bruker Avance III HD 400 MHz, a Bruker Avance III HD 500 MHz, and a Bruker Avance NEO 600 MHz instrument.

### Crystallographic Details

Crystals were selected from a Schlenk flask under an argon atmosphere, then put into a droplet of perfluorinated polyether oil on a microscope slide, and immediately shock cooled using the X‐TEMP2 device.^[^ ^67^, ^68^ ^]^ The diffraction data were collected using Mo Kα radiation and a Bruker Photon III C7 Detector. The data were integrated with *SAINT*.^[^ ^69^ ^]^ A multi‐scan absorption correction was applied using *SADABS* ^[^ ^70^ ^]^ or *TWINABS*.^[^ ^71^ ^]^ The structures were solved by *SHELXT* ^[^ ^72^ ^]^ and refined on *F* ^2^ using *SHELXL*.^[^ ^73^ ^]^ in the graphical user interface *ShelXle*.^[^ ^74^ ^]^ All hydrogen atoms bond to carbon atoms were placed according to geometrical criteria and refined with a riding model.

### Computational Details

Single‐molecule calculations were performed with the use of Gaussian16 software^[^ ^75^ ^]^ at the B3LYP/6–31+G(d) level of theory, which offers a balance between accuracy and computational cost. Coordinates were taken from crystallographic data and were kept frozen for further calculations. Such obtained wave function was further used to perform QTAIM partitioning of electron density with the use of AIMAll software.^[^ ^76^ ^]^ Natural bonding analysis of compound **1** was performed with the natural bond orbital^[^ ^77^ ^]^ (NBO 3.1) partitioning scheme implemented in the Gaussian16. Natural charges and Wiberg bond indices^[^ ^78^ ^]^ (WBI) were obtained directly from NBO analysis. To evaluate the free energy changes associated with the proposed mechanism, initial geometry optimizations were performed at the HF/6–31G(d) level in toluene, modelled with the polarizable continuum model (PCM). These optimized geometries served as the starting point for subsequent refinements that incorporated electron correlation effects using the B3LYP functional with the same basis set. More detailed information on these free energy calculations is provided in the Supporting Information.

## Supporting Information

Experimental and characterization details for all new complexes, including spectroscopic, crystallographic, and theoretical data are available in the Supporting Information.

## Conflict of Interests

The authors declare no conflict of interest.

## Supporting information



Supporting Information

Supporting Information

## Data Availability

The data that support the findings of this study are available in the supplementary material of this article.

## References

[1] P. P. Power , Nature 2010, 463, 171–177. 20075912 10.1038/nature08634

[2] D. W. Stephan , Acc. Chem. Res. 2015, 48, 306–316. 25535796 10.1021/ar500375j

[3] M. S. Hill , D. J. Liptrot , C. Weetman , Chem. Soc. Rev. 2016, 45, 972–988. 26797470 10.1039/c5cs00880h

[4] T. Chu , G. I. Nikonov , Chem. Rev. 2018, 118, 3608–3680. 29558125 10.1021/acs.chemrev.7b00572

[5] C. Weetman , S. Inoue , ChemCatChem 2018, 10, 4213–4228.

[6] R. L. Melen , Science 2019, 363, 479–484. 30705183 10.1126/science.aau5105

[7] C. W. Andersen , E. Borfecchia , M. Bremholm , M. R. V. Jørgensen , P. N. R. Vennestrøm , C. Lamberti , L. F. Lundegaard , B. B. Iversen , Angew. Chem. Int. Ed. 2017, 56, 10367–10372. 10.1002/anie.201703808 28670829

[8] A. F. Holleman , E. Wiberg , N. Wiberg , G. Fischer , Anorganische Chemie, De Gruyter, Berlin, Boston, 2017.

[9] G. Chen , G. Liu , Y. Pan , G. Liu , X. Gu , W. Jin , N. Xu , Chem. Soc. Rev. 2023, 52, 4586–4602. 37377411 10.1039/d3cs00370a

[10] N. Kosinov , J. Gascon , F. Kapteijn , E. J. Hensen , J. Membr. Sci. 2016, 499, 65–79.

[11] C. Dohmeier , D. Loos , H. Schnöckel , Angew. Chem. Int. Ed. 1996, 35, 129–149.

[12] H. W. Roesky , Inorg. Chem. 2004, 43, 7284–7293. 15530077 10.1021/ic0400641

[13] P. Bag , C. Weetman , S. Inoue , Angew. Chem. Int. Ed. 2018, 57, 14394–14413. 10.1002/anie.201803900 29790227

[14] J. Hicks , P. Vasko , J. M. Goicoechea , S. Aldridge , Angew. Chem. Int. Ed. 2021, 60, 1702–1713. 10.1002/anie.202007530 32567755

[15] M. He , C. Hu , R. Wei , X.‐F. Wang , L. L. Liu , Chem. Soc. Rev. 2024, 53, 3896–3951. 38436383 10.1039/d3cs00784g

[16] C. Dohmeier , C. Robl , M. Tacke , H. Schnöckel , Angew. Chem. Int. Ed. 1991, 30, 564–565.

[17] J. Hicks , P. Vasko , J. M. Goicoechea , S. Aldridge , Nature 2018, 557, 92–95. 29662211 10.1038/s41586-018-0037-y

[18] H. W. Roesky , S. S. Kumar , Chem. Commun. 2005, 4027. 10.1039/b505307b 16091791

[19] R. J. Baker , C. Jones , Coord. Chem. Rev. 2005, 249, 1857–1869.

[20] S. Nagendran , H. W. Roesky , Organometallics 2008, 27, 457–492.

[21] Y. Liu , J. Li , X. Ma , Z. Yang , H. W. Roesky , Coord. Chem. Rev. 2018, 374, 387–415.

[22] C. Bakewell , K. Hobson , C. J. Carmalt , Angew. Chem. Int. Ed. 2022, 61, e202205901. 10.1002/anie.202205901 PMC9401008 35474268

[23] M. Zhong , S. Sinhababu , H. W. Roesky , Dalton Trans. 2020, 49, 1351–1364. 31942579 10.1039/c9dt04763h

[24] X. Zhang , Y. Mei , L. L. Liu , Chem. ‐ Eur. J. 2022, 28, e202202102. 35942883 10.1002/chem.202202102

[25] K. Lammertsma , O. F. Guener , R. M. Drewes , A. E. Reed , P. v. R. Schleyer , Inorg. Chem. 1989, 28, 313–317.

[26] W. Uhl , Z. Naturforsch. B 1988, 43, 1113–1118.

[27] W. Uhl , Angew. Chem. Int. Ed. Engl. 1993, 32, 1386–1397.

[28] W. Uhl , Coord. Chem. Rev. 1997, 163, 1–32.

[29] G. Linti , H. Schnöckel , Coord. Chem. Rev. 2000, 206–207, 285–319.

[30] W. Uhl , Adv. Organomet. Chem. 2004, 51, 53.

[31] R. J. Wright , A. D. Phillips , P. P. Power , J. Am. Chem. Soc. 2003, 125, 10784–10785. 12952447 10.1021/ja034478p

[32] S. G. Minasian , J. Arnold , Chem. Commun. 2008, 4043. 10.1039/b806804f 18758620

[33] T. Agou , K. Nagata , H. Sakai , Y. Furukawa , N. Tokitoh , Organometallics 2012, 31, 3806–3809.

[34] A. Hofmann , A. Lamprecht , O. F. González‐Belman , R. D. Dewhurst , J. O. C. Jiménez‐Halla , S. Kachel , H. Braunschweig , Chem. Commun. 2018, 54, 1639–1642. 10.1039/c7cc09596a 29376161

[35] B. Li , S. Kundu , H. Zhu , H. Keil , R. Herbst‐Irmer , D. Stalke , G. Frenking , D. M. Andrada , H. W. Roesky , Chem. Commun. 2017, 53, 2543–2546. 10.1039/c7cc00325k 28180224

[36] J. Kretsch , A. Kreyenschmidt , T. Schillmöller , R. Herbst‐Irmer , D. Stalke , Inorg. Chem. 2020, 59, 13690–13699. 32897060 10.1021/acs.inorgchem.0c02066

[37] S. J. Bonyhady , D. Collis , G. Frenking , N. Holzmann , C. Jones , A. Stasch , Nat. Chem 2010, 2, 865–869. 20861903 10.1038/nchem.762

[38] T. Chu , I. Korobkov , G. I. Nikonov , J. Am. Chem. Soc. 2014, 136, 9195–9202. 24893309 10.1021/ja5038337

[39] R. L. Falconer , K. M. Byrne , G. S. Nichol , T. Krämer , M. J. Cowley , Angew. Chem. Int. Ed. 2021, 60, 24702–24708. 10.1002/anie.202111385 PMC8596890 34520616

[40] I. Squire , M. Tritto , J. Morell , C. Bakewell , Chem. Commun. 2024, 60, 12908–12911. 10.1039/d4cc03904a 39417244

[41] X. Wang , F. Rüttger , A. Krawczuk , R. Herbst‐Irmer , D. Stalke , Eur. J. Inorg. Chem. 2023, 27, e202300629.

[42] X. Wang , R. F. Ligorio , F. Rüttger , D. M. J. Krengel , N. Graw , R. Herbst‐Irmer , A. Krawczuk , D. Stalke , Dalton Trans. 2024, 53, 15441–15450. 39120603 10.1039/d4dt01798f

[43] N. J. Hardman , C. Cui , H. W. Roesky , W. H. Fink , P. P. Power , Angew. Chem. Int. Ed. 2001, 40, 2172–2174. 10.1002/1521-3773(20010601)40:11<2172::AID-ANIE2172>3.0.CO;2-Y 29712210

[44] J. D. Queen , S. Irvankoski , J. C. Fettinger , H. M. Tuononen , P. P. Power , J. Am. Chem. Soc. 2021, 143, 6351–6356. 33882237 10.1021/jacs.1c02463 PMC8154528

[45] L. Zhu , R. Kinjo , Chem. Soc. Rev. 2023, 52, 5563–5606. 37519098 10.1039/d3cs00290j

[46] D. Dhara , F. Fantuzzi , M. Härterich , R. D. Dewhurst , I. Krummenacher , M. Arrowsmith , C. Pranckevicius , H. Braunschweig , Chem. Sci. 2022, 13, 9693–9700. 36091914 10.1039/d2sc02783f PMC9400590

[47] H. Zhu , Z. Yang , J. Magull , H. W. Roesky , H.‐G. Schmidt , M. Noltemeyer , Organometallics 2005, 24, 6420–6425.

[48] W. L. Cao , C. Gatti , P. J. MacDougall , R. Bader , Chem. Phys. Lett. 1987, 141, 380–385.

[49] J. A. Platts , J. Overgaard , C. Jones , B. B. Iversen , A. Stasch , J. Phys. Chem. A 2011, 115, 194–200. 21158464 10.1021/jp109547w

[50] L. Werner , J. Hagn , U. Radius , Chem. ‐ Eur. J. 2023, 29, e202303111. 37792718 10.1002/chem.202303111

[51] R. Yamanashi , K. Mizutani , M. Yamashita , Chem. ‐ Eur. J. 2025, 31, e202403926. 39632272 10.1002/chem.202403926

[52] K. Koshino , R. Kinjo , J. Am. Chem. Soc. 2021, 143, 18172–18180. 34697939 10.1021/jacs.1c07389

[53] F. Lu , X. Li , Z. Sun , Y. Zeng , L. Meng , Dalton Trans. 2015, 44, 14092–14100. 26171664 10.1039/c5dt01901j

[54] W. Haider , D. M. Andrada , I.‐A. Bischoff , V. Huch , A. Schäfer , Dalton Trans. 2019, 48, 14953–14957. 31577309 10.1039/c9dt03470f

[55] M. Michalski , A. J. Gordon , S. Berski , J. Mol. Model 2019, 25, 211. 31273474 10.1007/s00894-019-4075-7 PMC7406486

[56] R. F. W. Bader , Atoms in Molecules. A Quantum Theory, Clarendon Press; Oxford University Press, Oxford England, New York, 1994.

[57] R. Neufeld , D. Stalke , Chem. Sci. 2015, 6, 3354–3364. 29142693 10.1039/c5sc00670h PMC5656982

[58] S. Bachmann , B. Gernert , D. Stalke , Chem. Commun. 2016, 52, 12861–12864. 10.1039/c6cc07273a 27731871

[59] S. Bachmann , R. Neufeld , M. Dzemski , D. Stalke , Chem. ‐ Eur. J. 2016, 22, 8462–8465. 27061592 10.1002/chem.201601145

[60] A.‐K. Kreyenschmidt , S. Bachmann , T. Niklas , D. Stalke , ChemistrySelect 2017, 2, 6957–6960.

[61] A.‐K. Kreyenschmidt , M. John , D. Stalke , Organometallics 2023, 42, 2957–2962.

[62] C. Cui , H. W. Roesky , H.‐G. Schmidt , M. Noltemeyer , Angew. Chem. Int. Ed. 2000, 39, 4531–4533. 11169660

[63] A. Heilmann , J. Hicks , P. Vasko , J. M. Goicoechea , S. Aldridge , Angew. Chem. Int. Ed. 2020, 59, 4897–4901. 10.1002/anie.201916073 31999037

[64] D. Dhara , A. Jayaraman , M. Härterich , R. D. Dewhurst , H. Braunschweig , Chem. Sci. 2022, 13, 5631–5638. 35694334 10.1039/d2sc01436j PMC9116349

[65] J. M. Léon Baeza , H. Xu , S. Takahashi , A. Baceiredo , R. S. Rojas Guerrero , D. Hashizume , N. Saffon‐Merceron , V. Branchadell , T. Kato , Angew. Chem. Int. Ed. 2025, 64, e202505181. 10.1002/anie.202505181 PMC12124428 40162864

[66] S.‐K. Chen , W.‐Q. Ma , Z.‐B. Yan , F.‐M. Zhang , S.‐H. Wang , Y.‐Q. Tu , X.‐M. Zhang , J.‐M. Tian , J. Am. Chem. Soc. 2018, 140, 10099–10103. 30067344 10.1021/jacs.8b05386

[67] T. Kottke , D. Stalke , J. Appl. Crystallogr. 1993, 26, 615–619.

[68] D. Stalke , Chem. Soc. Rev. 1998, 27, 171.

[69] Bruker AXS Inc ., SAINT v8.40B, Madison, WI, USA, 2019.

[70] L. Krause , R. Herbst‐Irmer , G. M. Sheldrick , D. Stalke , J. Appl. Crystallogr. 2015, 48, 3–10. 26089746 10.1107/S1600576714022985 PMC4453166

[71] M. Sevvana , M. Ruf , I. Usón , G. M. Sheldrick , R. Herbst‐Irmer , Acta Crystallogr. D 2019, 75, 1040–1050. 10.1107/S2059798319010179 PMC6889912 31793898

[72] G. M. Sheldrick , Acta Crystallogr. A 2015, 71, 3–8. 10.1107/S2053273314026370 PMC4283466 25537383

[73] G. M. Sheldrick , Acta Crystallogr. C 2015, 71, 3–8. 10.1107/S2053273314026370 PMC4283466 25537383

[74] C. B. Hübschle , G. M. Sheldrick , B. Dittrich , J. Appl. Crystallogr. 2011, 44, 1281–1284. 22477785 10.1107/S0021889811043202 PMC3246833

[75] M. J. Frisch , G. W. Trucks , H. B. Schlegel , G. E. Scuseria , M. A. Robb , J. R. Cheeseman , G. Scalmani , V. Barone , G. A. Petersson , H. Nakatsuji , X. Li , M. Caricato , A. V. Marenich , J. Bloino , B. G. Janesko , R. Gomperts , B. Mennucci , H. P. Hratchian , J. V. Ortiz , A. F. Izmaylov , J. L. Sonnenberg , D. Williams‐Young , F. Ding , F. Lipparini , F. Egidi , J. Goings , B. Peng , A. Petrone , T. Henderson , D. Ranasinghe , et al., Gaussian16, Gaussian Inc., Wallingford CT, 2016.

[76] AIMAII (Version 19.10.12), Todd A. Keith, Overland Park KS, USA, 2019 (aim.tkgristmill.com).

[77] E. D. Glendening , A. E. Reed , J. E. Carpenter , F. Weinhold , NBO Version 3.1: natural bond orbital analysis program 2001.

[78] K. B. Wiberg , Tetrahedron 1968, 24, 1083–1096.

