# Supplementary material for: Intriguing Reactivity of a 1,2‐Dihydrodialumane Towards Organic Azides – From a Terminal Diazido–Dialumane to Pendulum‐Clock‐Like Azide Bridging
Source: Angew Chem Int Ed Engl. 2025 Jul 4;64(33):e202503638. doi: 10.1002/anie.202503638 (PMC12338445; doi:10.1002/anie.202503638)
Supplement: Supplementary file 1 — Supporting Information [file ANIE-64-e202503638-s002.pdf]

Intriguing Reactivity of a 1,2-Dihydrodialumane towards Organic Azides – From A Terminal Diazido–Dialumane to *Pendulum-Clock-Like* Azide Bridging

Xiaobai Wang[a], Franziska Traeger[a], Raphael F. Ligorio[a], Nico Graw[a], Regine Herbst-Irmer[a], Anna Krawczuk[a], Malte Fischer[a], and Dietmar Stalke*[a]

[a]: Institut für Anorganische Chemie
Georg-August-University Göttingen
Tammannstaße 4, 37077 Göttingen, Germany
E-Mail: dstalke@chemie.uni-goettingen.de

Contents

General Procedures 3
Synthesis and Analytical Data 4
Complex 1 4
Complex 2 7
Complex 3 14
Complex 4 18
Complex 5 19
Photophysical Data 24
General Data Acquisition and Processing 25
Crystal structure of 1 27
Crystal Structure of 2 31
Crystal Structure of 3 34
Crystal Structure of 4 38
Crystal Structure of 5 43
Computational Details 47
Reference 51

General Procedures

The ^{1}H, ^{13}C, ^{15}N and 2D NMR spectroscopic data were recorded on a Bruker Avance III 300 MHz, a Bruker Avance III HD 400 MHz, a Bruker Avance III HD 500 MHz and a Bruker Avance NEO 600 MHz instrument. The measurements were carried out at room temperature in solutions of deuterated solvents. For reasons of solubility and comparison, the most used deuterated solvent for ^{1}H-NMR spectra is toluene-d_8. The chemical shifts δ are given in ppm and the coupling constants J in Hz. The observed multiplicities are abbreviated as follows: s = singlet, d = doublet, t = triplet, q = quartet, m = multiplet. Young-type Teflon-valve NMR tubes have been used throughout this work. The crystallographic information files (CIF) can be obtained free of charge from the Cambridge Crystallographic Data Centre (CCDC nos. see **Table S1**).

Synthesis and Analytical Data

Complex 1

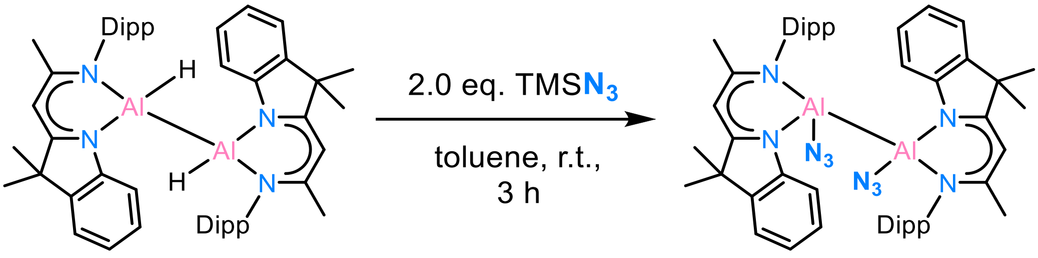

I | **1**, 78%

Dialane **I** (0.021 g, 0.026 mmol, 1.0 eq.) and trimethylsilyl azide (6.10 mg, 7.00 µL, 0.053 mmol, 2.0 eq) were added to 0.5 mL of toluene-d_8 and transferred to a JY NMR tube. The reaction was monitored by ^{1}H NMR spectroscopy. After 3 hours, the measured ^{1}H NMR spectrum indicated the completion of the reaction. The solvent was removed *in vacuo*, *n*-hexane was added and the sample was dried to remove all volatiles. Crystals suitable for SC-XRD analysis were grown in toluene at -30 °C over 1 hour. The deep yellow crystals were isolated and dried *in vacuo*. A second crop of yellow crystals were grown from the concentrated mother liquor at -30 °C (total yield: 17.70 mg, 0.021 mmol, 78%). LIFDI (positive mode, toluene): Calcd. for $C_{50}H_{62}Al_2N_{10}$: 856.5; Found: 856.2. Elemental analysis: Calcd: C 70.07, H 7.29, N 16.34, found: C 68.69, H 7.68, N 15.72. **Note**: Complex **1** slowly decomposes at low temperature (−30 °C), room temperature and immediately decomposes at elevated temperatures (110 °C). Attempts to crystallise the decomposition products using different solvents were unsuccessful. NMR analysis of crystalline material in toluene-d_8, benzene-d_6 and THF-d_8 were attempted several times but no satisfactory data could be obtained. Elemental analysis of crystalline material were attempted several times but due to slow N_2 losses, the percentage of N was always lower than expected.

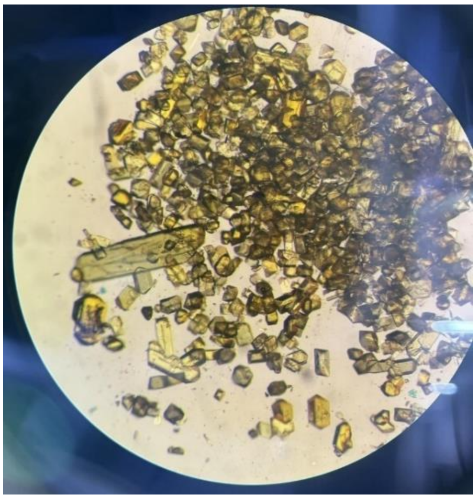

Figure S1: Crystals of complex **1** under the microscope.

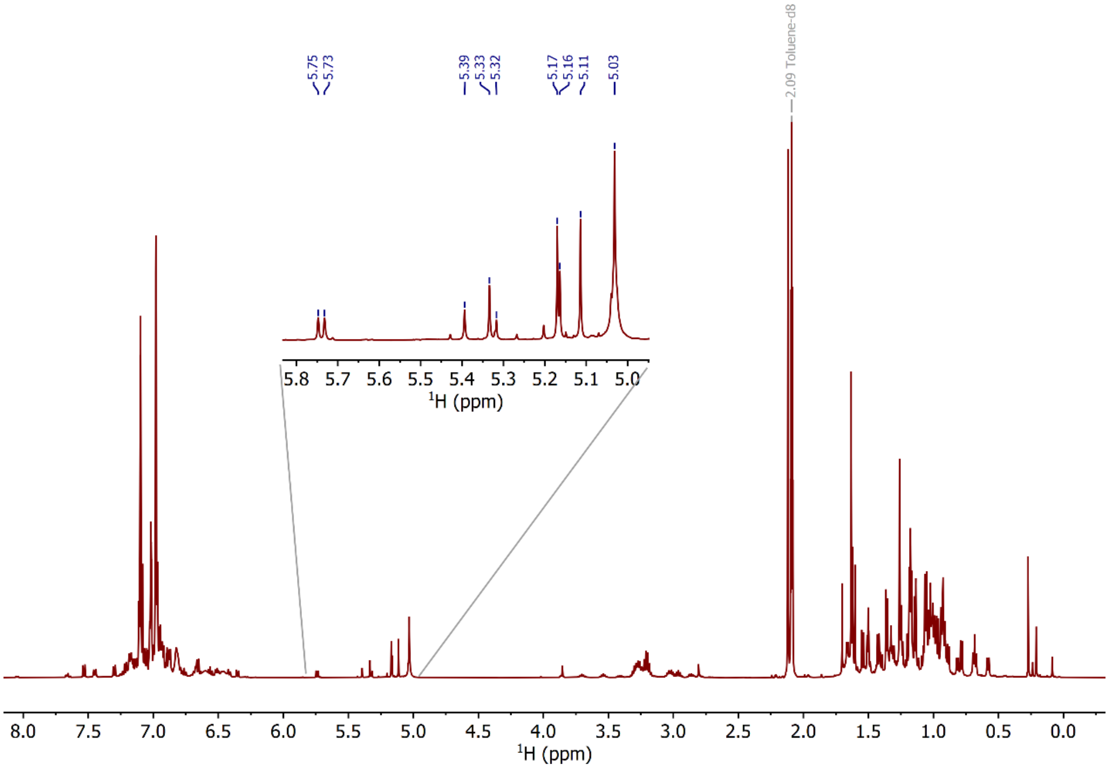

Figure S2: 500.3 MHz ^{1}H NMR spectrum of complex **1** in toluene-d_8.

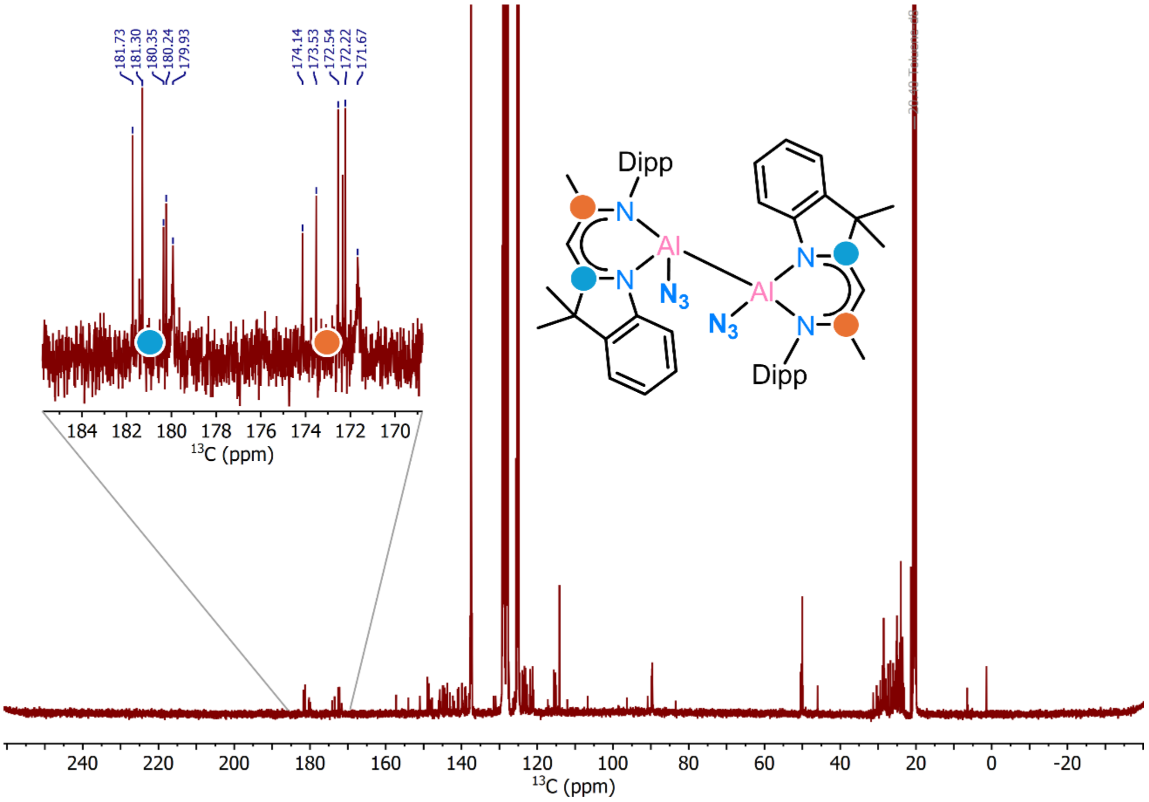

Figure S3: 125.8 MHz $^{13}C\{^{1}H\}$ NMR spectrum of complex **1** in toluene-d_8.

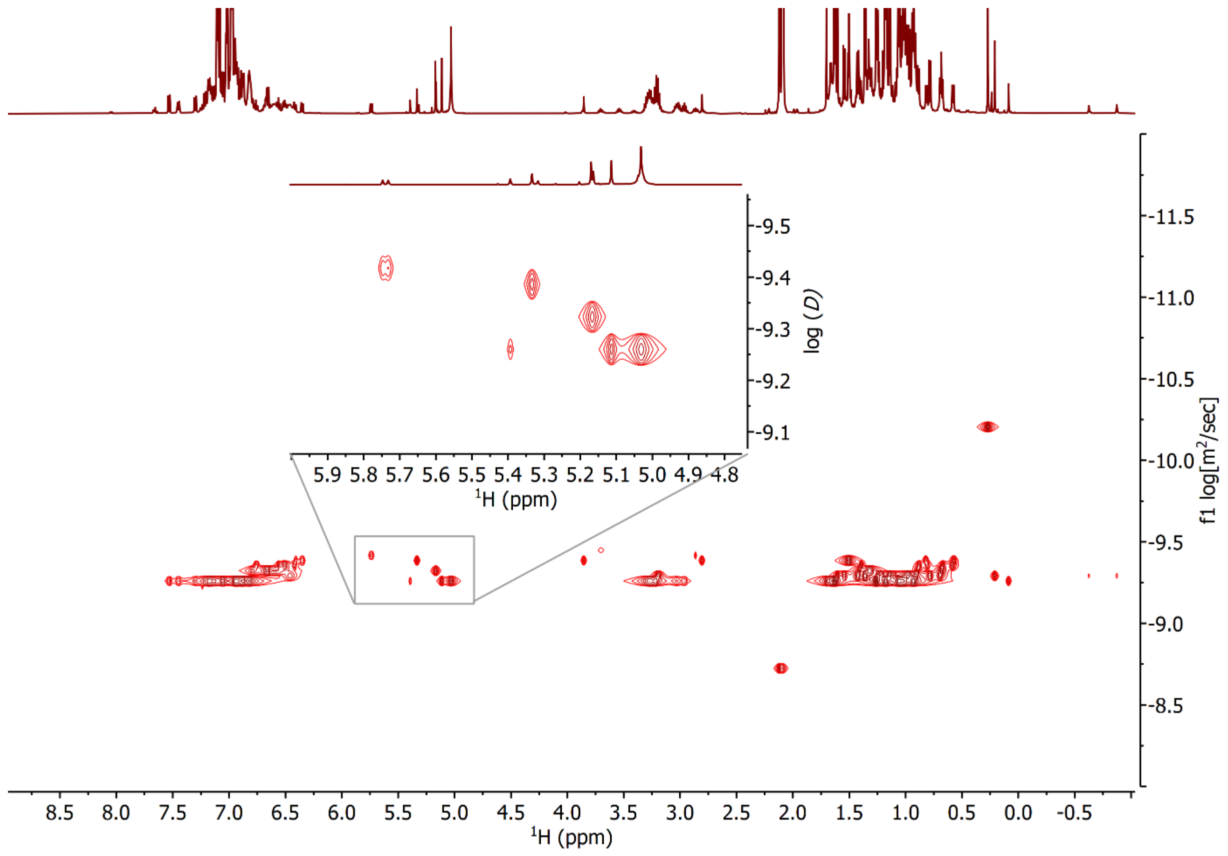

Figure S4: 500.3 MHz ^{1}H DOSY spectrum of **1** in toluene-d_8 at room temperatures (298 K).

Complex 2

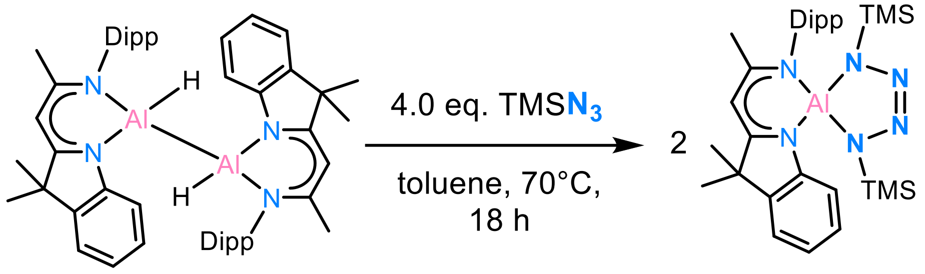

Route 1: Dialane **I** (0.023 g, 0.030 mmol, 1.0 eq.) and trimethylsilyl azide (13.85 mg, 15.92 µL, 0.120 mmol, 4.0 eq) were added dropwise to 0.5 ml toluene-d_8 and transferred to a JY NMR tube. The reaction was heated to 70 °C, and after 18 hours, ^{1}H NMR indicated the completion of the reaction. The solvent was removed *in vacuo*, *n*-hexane was added and the sample was dried to remove all volatiles. Crystals suitable for SC-XRD analysis were grown from a toluene/*n*-hexane (1:1) mixture at -30 °C. The bright yellow crystals were isolated and dried *in vacuo* (13.19 mg, 0.034 mmol, 65%; when $TMSN_3$ 2.0 eq., 43%). LIFDI (positive mode, toluene): Calcd. for $C_{31}H_{49}AlN_6Si_2$: 588.3; Found: 588.6. Elemental analysis: Calcd: C 63.22, H 8.39, N 14.27, found: C 65.76, H 8.14, N 13.92. ^{1}H NMR (298 K, toluene-d_8, 500.3 MHz): δ (ppm) = 7.79 (dd, 1H, 2-H, J = 7.95, 1.65 Hz), 6.87-7.17 (m, 6H, 3-H, 4-H, 5-H, 16-H, 17-H, 18-H), 5.08 (s, 1H, 11-H), 3.50 (hept, 1H, 23-H, J = 6.78 Hz), 3.11 (hept, 1H, 20-H, J = 6.74 Hz), 1.59 (s, 3H, 13-H), 1.50 (d, 3H, 22-H, J = 6.68 Hz), 1.40 (d, 3H, 25-H, J = 6.79 Hz), 1.21 (s, 3H, 8-H), 1.07 (s, 3H, 9-H), 1.06 (d, 3H, 24-H, J = 7.02 Hz), 1.02 (d, 3H, 21-H, J = 6.80 Hz), 0.25 (s, 9H, 29/30/31-H), 0.08 (s, 9H, 26/27/28-H). ^{13}C NMR (298 K, toluene-d_8, 125.8 MHz): δ (ppm) = 181.3 (1C, 10-C), 173.8 (1C, 12-C), 148.2 (1C, 1-C), 145.8 (1C, 19-C), 143.8 (1C, 15-C), 140.2 (1C, 6-C), 139.4 (1C, 14-C), 128.6 (1C, 3-C), 128.4 (1C, 17-C), 125.6 (1C, 18-C), 124.6 (1C, 16-C), 124.3 (1C, 4-C), 122.2 (1C, 5-C), 115.5 (1C, 2-C), 90.5 (1C, 11-C), 50.2 (1C, 7-C), 28.8 (1C, 20-C), 28.4 (1C, 23-C), 27.5 (1C, 9-C), 25.4 (1C, 21-C), 25.3 (1C, 8-C), 25.0 (1C, 25-C), 24.7 (1C, 24-C), 24.5 (1C, 13-C), 24.3 (1C, 22-C), 5.5 (3C, 26/27/28-C), 5.2 (3C, 29/30/31-C). ^{15}N NMR (298 K, toluene-d_8, 50.7 MHz): δ (ppm) = −200.3 (1N, 1-N), −205.2 (1N, 2-N), −341.6 (2N, 3/6-N). ^{29}Si NMR (298 K, toluene-d_8, 79.5 MHz): δ (ppm) = 2.5 (1Si, 1-Si), −1.6 (1Si, 2-Si). ^{27}Al NMR (298 K, toluene-d_8, 130.4 MHz): δ (ppm) = 77.5.

Route 2: Diazido-dialane **1** (16.0 mg, 0.019 mmol, 1.0 eq.) and trimethylsilyl azide (8.60 mg, 9.89 µL, 0.075 mmol, 4.0 eq.) were dissolved in 1 mL toluene. The sample was cooled and stored at -30 °C for one week. After that, the solvent was removed *in vacuo*, yielding the identical tetrazole complex **2**.

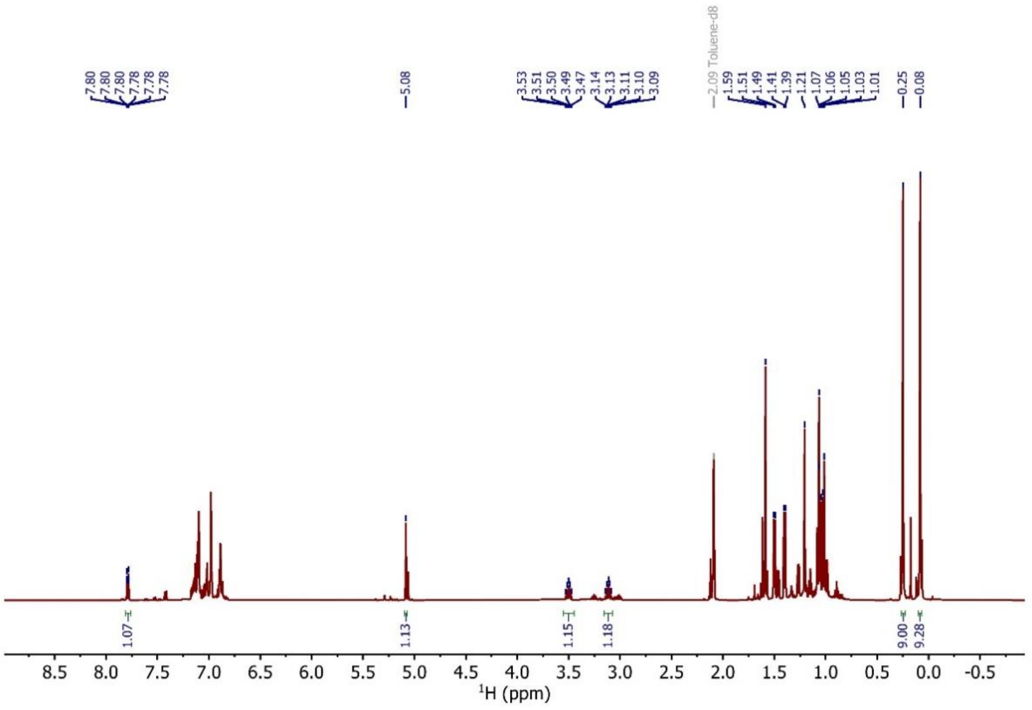

Figure S5. 500.3 MHz ^{1}H NMR spectrum of complex **2** in toluene-d_8.

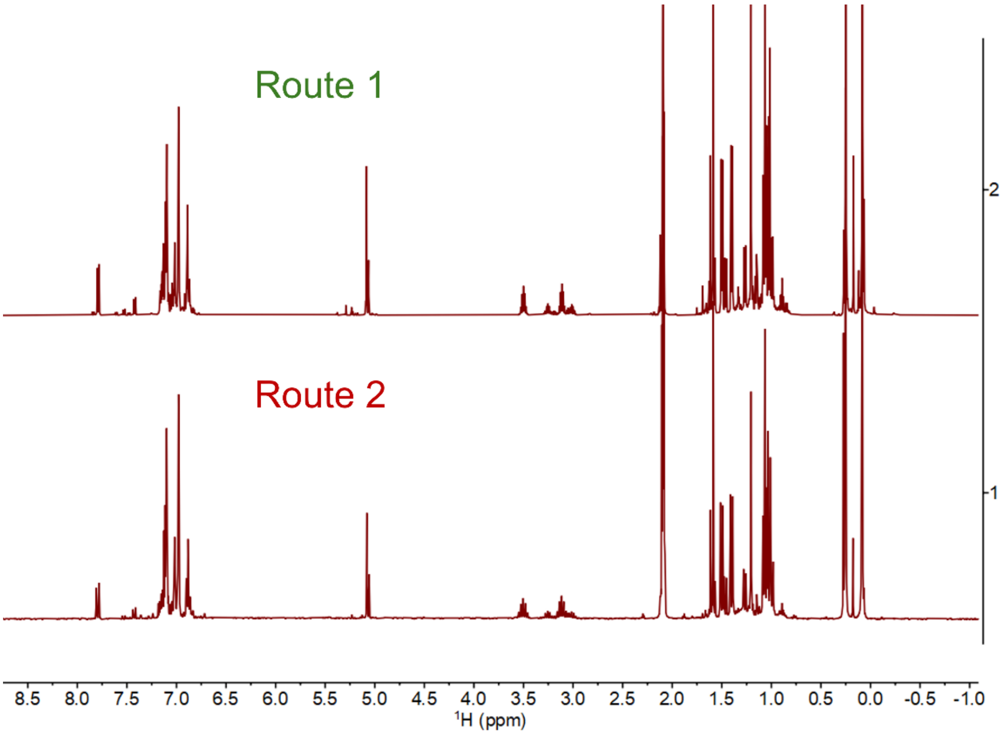

Figure S6: 500.3 MHz ^{1}H NMR spectrum of complex **2** in toluene-d_8 (**Route 1**, top; **Route 2**, bottom).

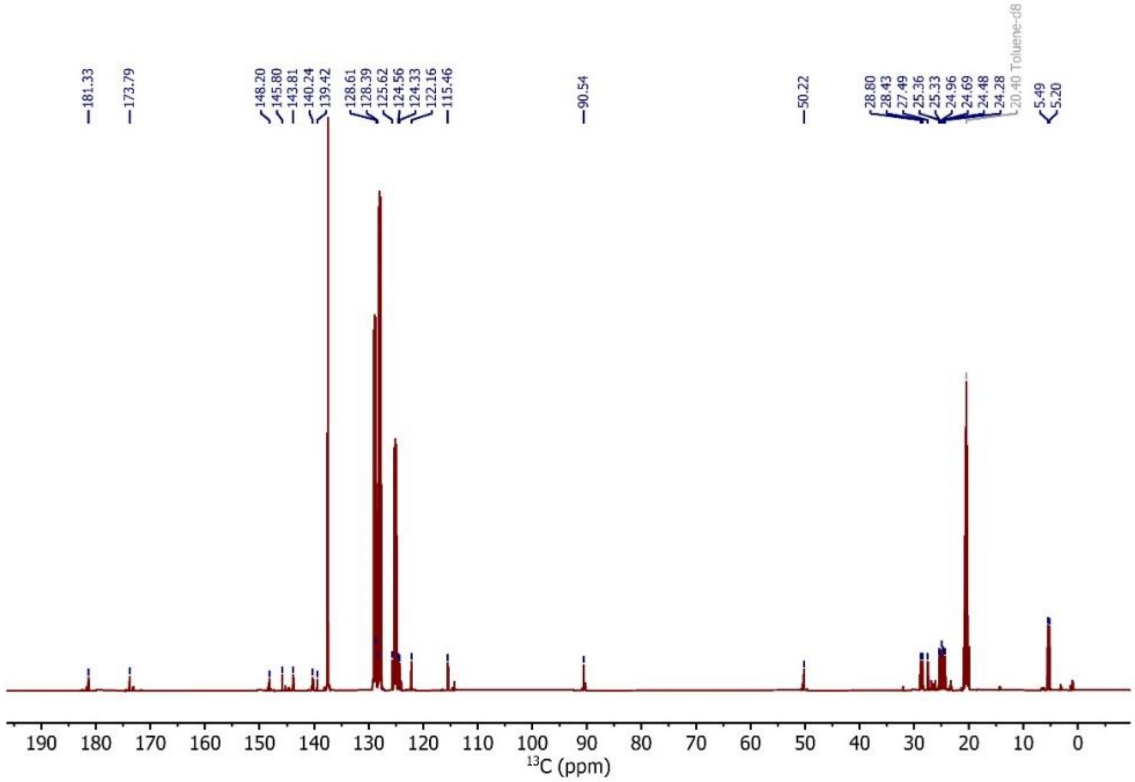

Figure S7. 125.8 MHz $^{13}C\{^{1}H\}$ NMR spectrum of complex **2** in toluene-d_8.

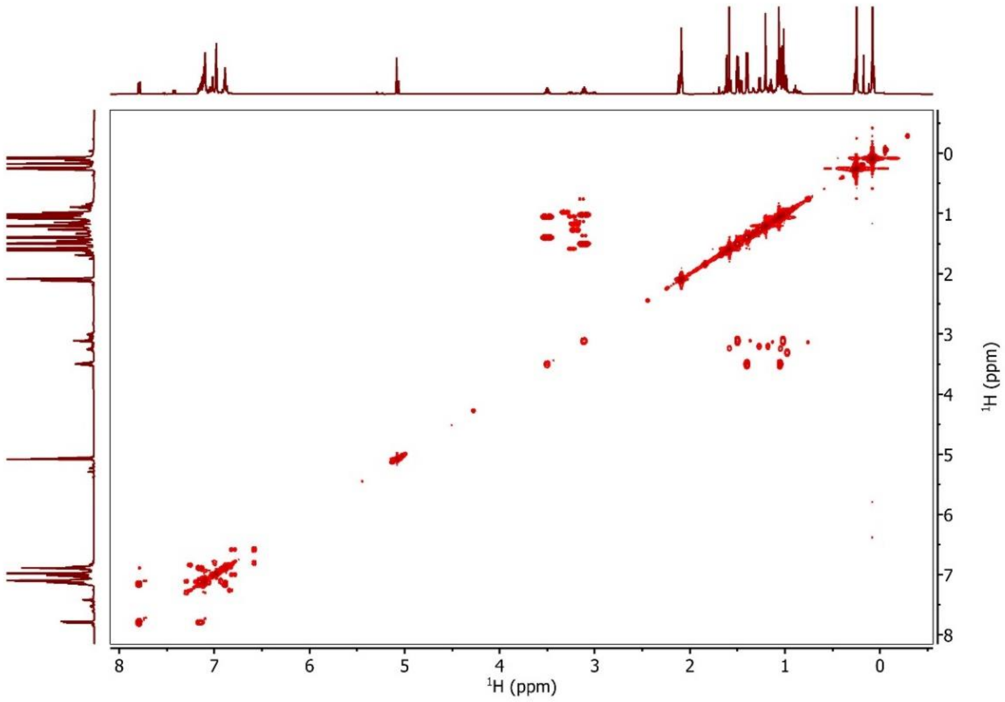

Figure S8. 400.3 MHz ^{1}H COSY spectrum of complex **2** in toluene-d_8.

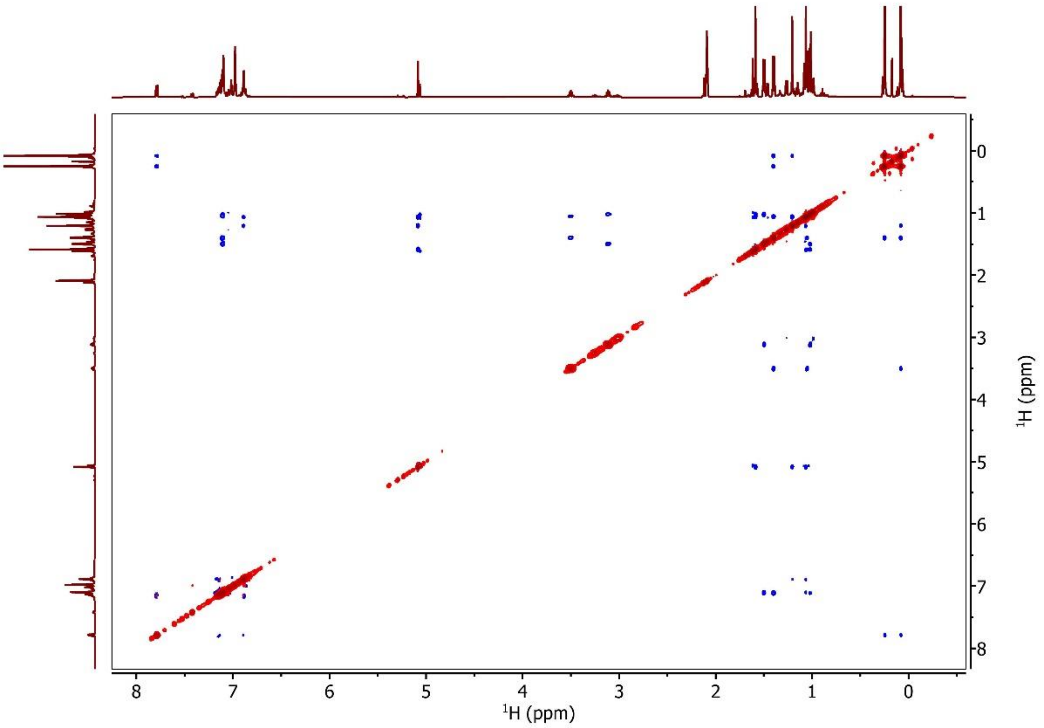

Figure S9. 500.3 MHz ^{1}H EXSY spectrum of complex **2** in toluene-*d*$_8$.

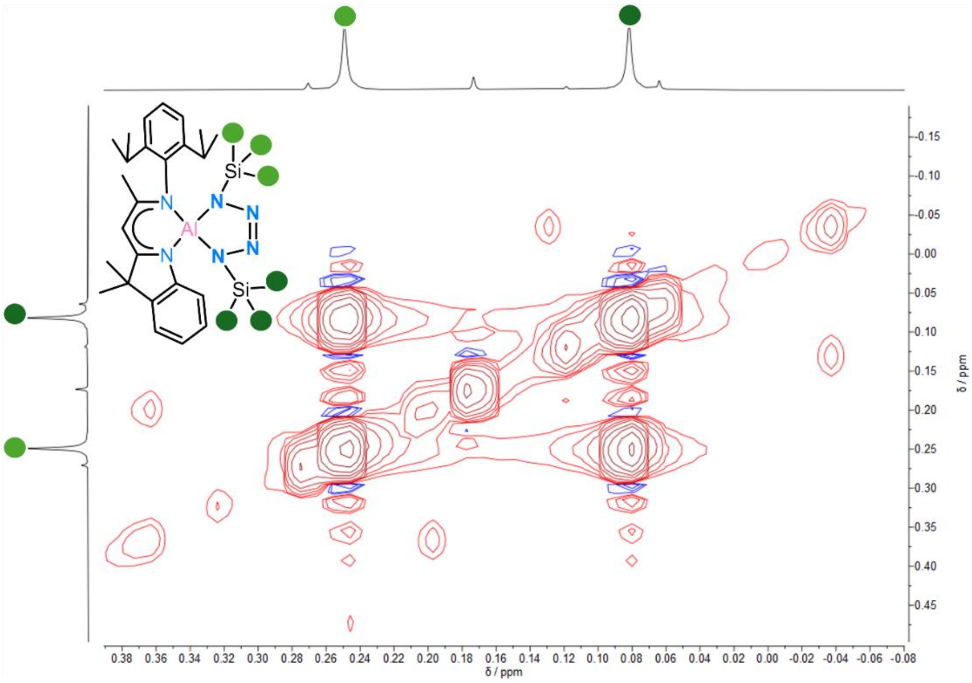

Figure S10. Excerpt of a 500.3 MHz ^{1}H EXSY spectrum of complex **2** in toluene-*d*$_8$ showing the TMS groups.

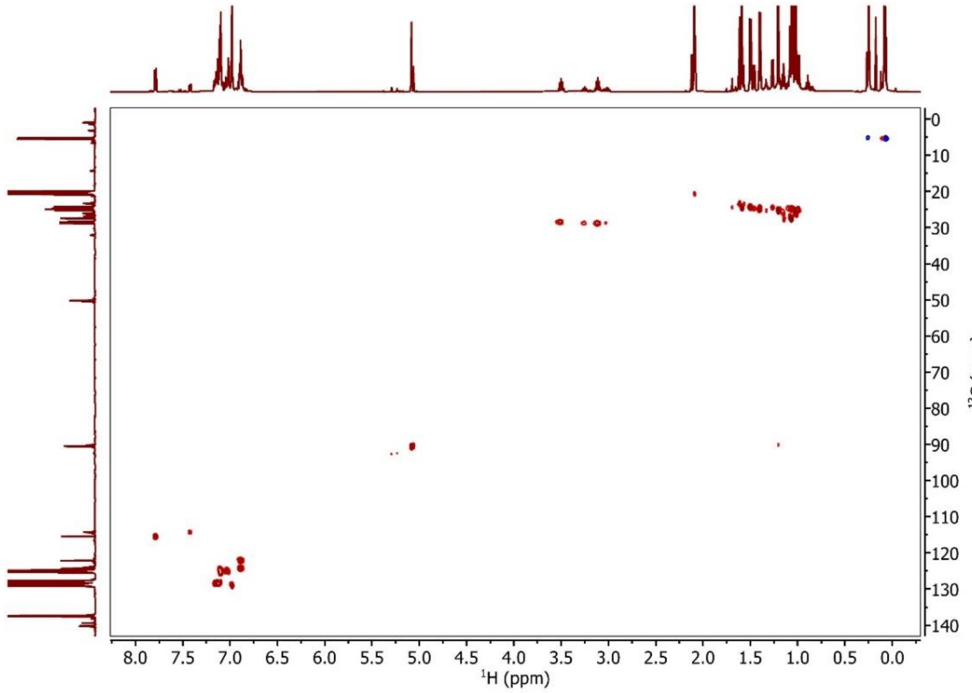

Figure S11. 500.3/125.8 MHz ^{1}H,^{13}C HSQC spectrum of complex **2** in toluene-d_8.

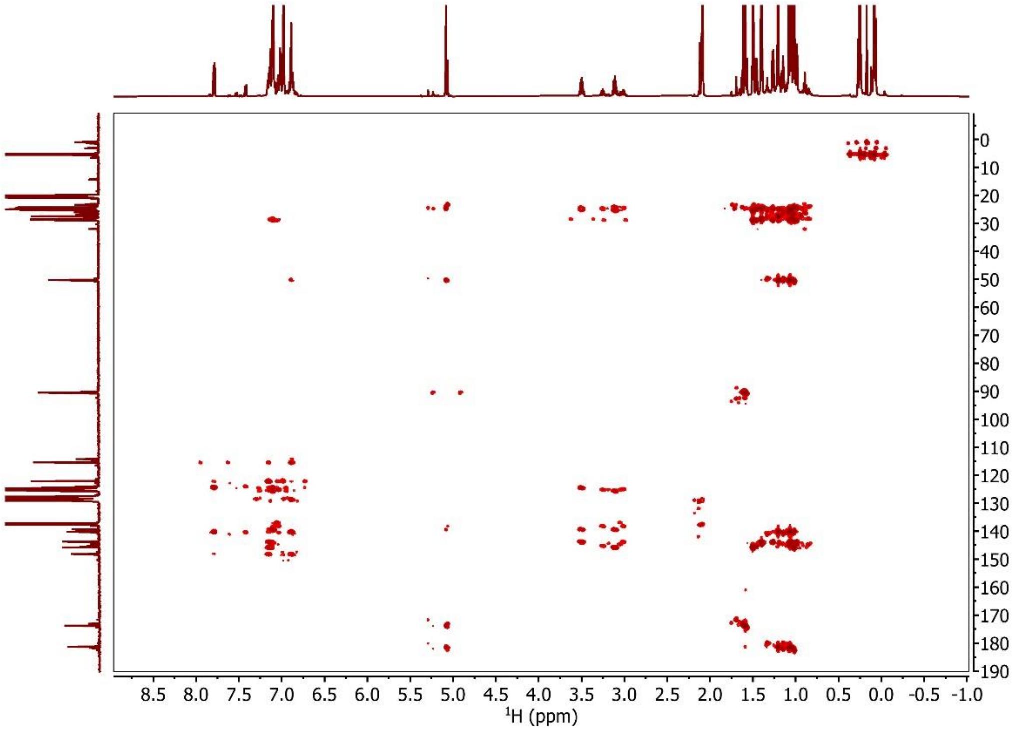

Figure S12. 500.3/125.8 MHz ^{1}H,^{13}C HMBC spectrum of complex **2** in toluene-d_8.

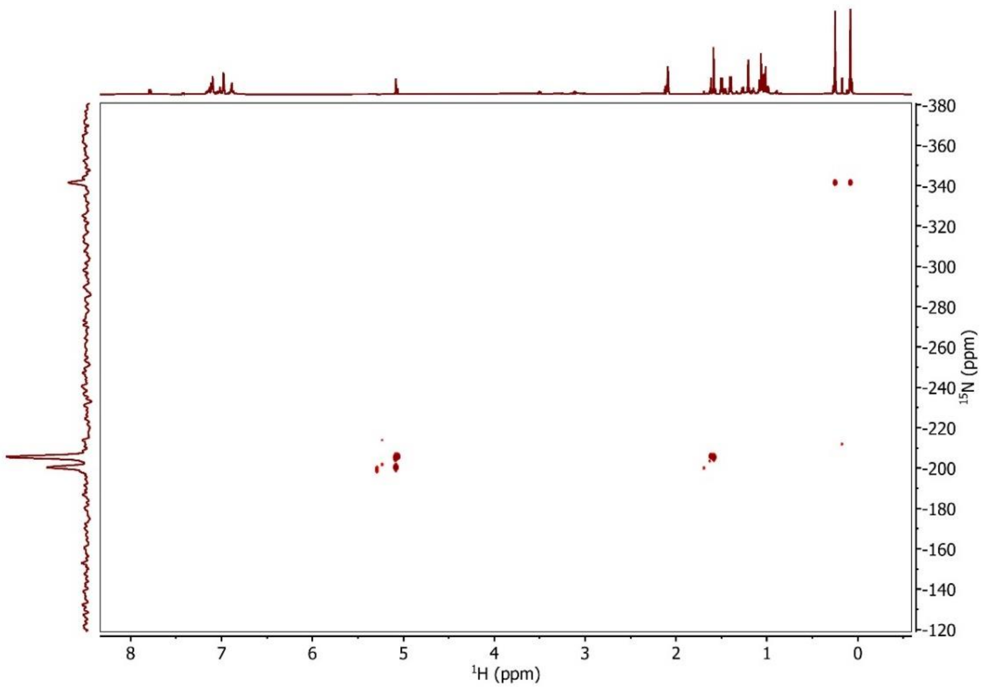

Figure S13. 500.3/50.7 MHz ^{1}H,^{15}N HMBC spectrum of complex **2** in toluene-d_8.

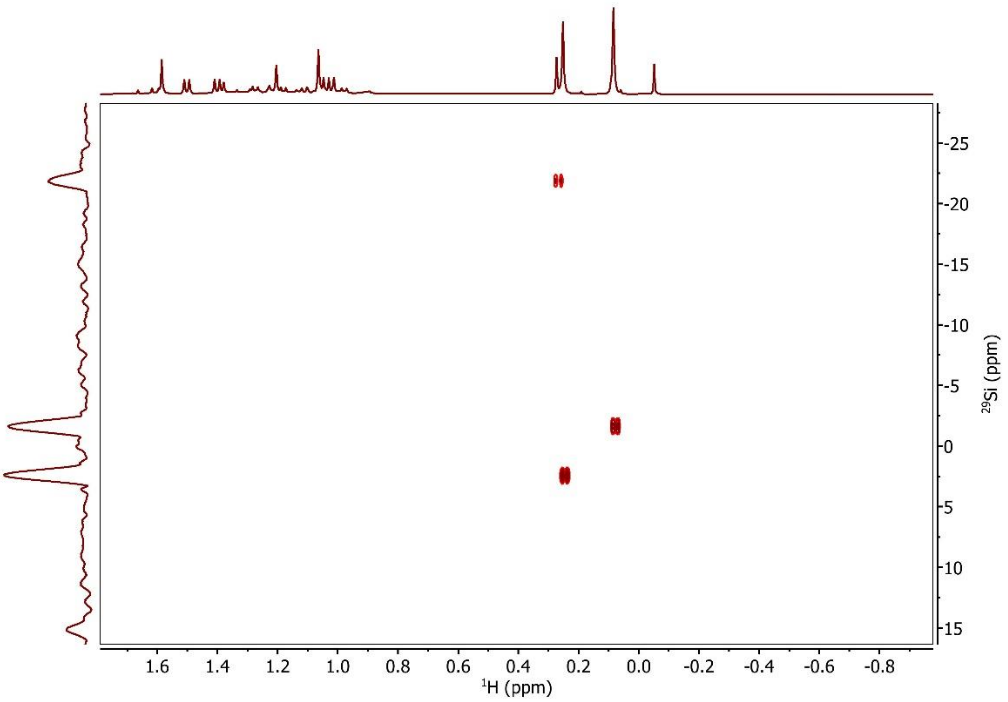

Figure S14. 400.3/79.5 MHz ^{1}H,^{29}Si HMBC spectrum of complex **2** in toluene-d_8.

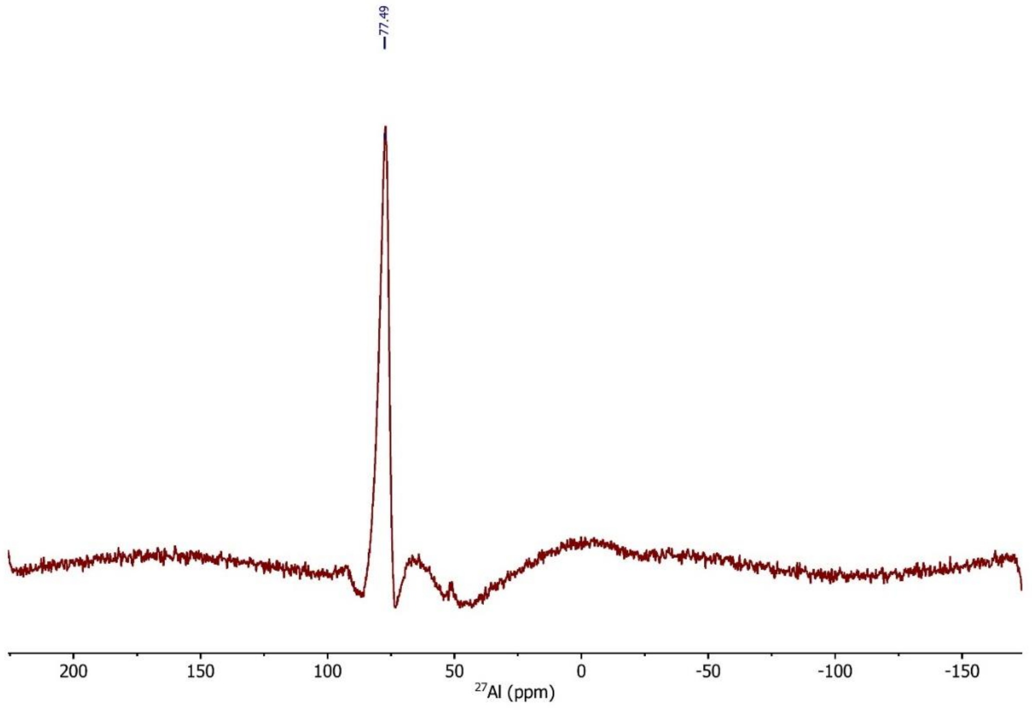

Figure S15. 130.4 MHz ^{27}Al NMR spectrum of complex **2** in toluene-d_8.

Complex 3

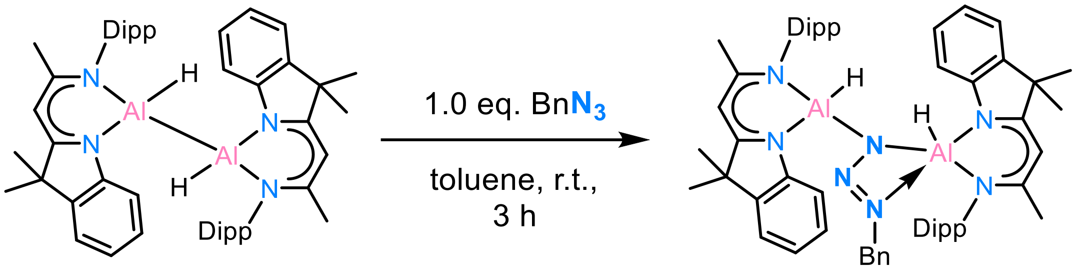

I **3**, 59%

Dialane (0.044 g, 0.056 mmol, 1.00 eq.) was dissolved in 5 mL toluene at ambient temperature. Benzyl azide (7.97 mg, 94%, 7.49 µL, 0.056 mmol) was slowly added dropwise to the solution of dialane **I**. The mixture turned light yellow within one hour. It was stirred continuously for an additional three hours, after which ^{1}H NMR confirmed the reaction to be completed. The solvent was then removed under reduced pressure. Crystals suitable for SC-XRD experiments were grown from a solvent mixture (toluene+*n*-hexan=1:1) at -30 °C after 1 day (30.2 mg, 0.033 mmol, 59%). ^{1}H NMR (298 K, tol-d_8, 500 MHz, solvent peak=2.09 ppm): 7.42 (d, 1H, 28-H, J = 7.9 Hz), 7.23 (m, Ar-H, 14H), 6.82 (t, 1H, 4-H, J = 14.5 Hz), 6.46 (t, 1H, 3-H, J = 15.0 Hz), 5.13, (s, 1H, 11-H), 4.99 (s, 1H, 36-H), 4.24 (d, 1H, 51-H, J = 14.1 Hz), 3.77 (q,1H, iPrCH), 3.19 (d, 1H, 51-H, J = 14.2 Hz), 3.13 (q, 1H, iPrCH), 2.86 (q, 1H, iPrCH), 2.62 (q, 1H, iPrCH), 1.79 (s, 3H, 13-H), 1.62 (s, 3H, 38-H), 1.47 (s, 3H, 8-H), 1.38 (d, 3H, iPr, J = 6.8 Hz), 1.26 (s, 3H, 33-H), 1.17 (s, 3H, 9-H), 1.08 (s, 3H, 9-H), 1.04 (m, 3H, iPr), 1.02 (m, 3H, iPr), 0.91 (m, 3H, iPr), 0.89 (solv. *n*-hexane), 0.79 (d, 3H, iPr, J = 6.5 Hz), 0.41 (d, 3H, iPr, J = 6.7 Hz). $^{13}C\{^{1}H\}$ NMR (298 K,tol-d_8, 126 MHz, solvent peak=20.40 ppm): 180.31 (10-C), 180.23 (35-C), 172.37 (12-C), 170.91 (37-C), 151.77 (1-H), 150.11 (26-C), 145.71 (14-C), 144.95 (39-C), 144.05 (15-C, 19-C), 143.83 (40-C, 44-C), 141.16 (6-C), 140.94 (31-C), 140.14 (51-C), 129.37 (53-C), 127.84 (54-C), 126.30 (41-C), 121.96 (4-C), 121.80 (29-C), 120.72 (5-C), 115.91 (2-C), 114.59 (21-C), 89.56 (11-C), 87.45 (36-C), 58.79 (51-C), 49.89 (7-C), 49.65 (31-C), 28.52 (20-C, iPrCH), 28.39 (45-C, iPrCH), 27.72 (23-C), 27.57 (23-C, iPrCH), 26.86 (8-C), 26.49 (33-C), 26.30 (9-C), 25.67 (21-C, iPrCH), 25.56 (9-C), 25.11 (21-C, iPrCH), 25.01 (13-C), 24.24 (24-C, iPr), 23.90 (22-C, iPr), 23.58 (38-C), 23.09 (49-C, iPr), 22.85 (50-C, iPr). Elemental analysis: $C_{57}H_{71}Al_2N_7$ calculated C 75.38, H 7.88, N 10.80, found C 75.12, H 7.66, N 11.25. LIFDI-MS (positive mode, toluene): 105.4 (100) $[BnN_3\text{-}2\times N]^+$, 389.9 (90) $[L+H]^+$, 879.3 (67) [**3**-2×N]$^+$, 908.3 (22) [**3**+H]$^+$.

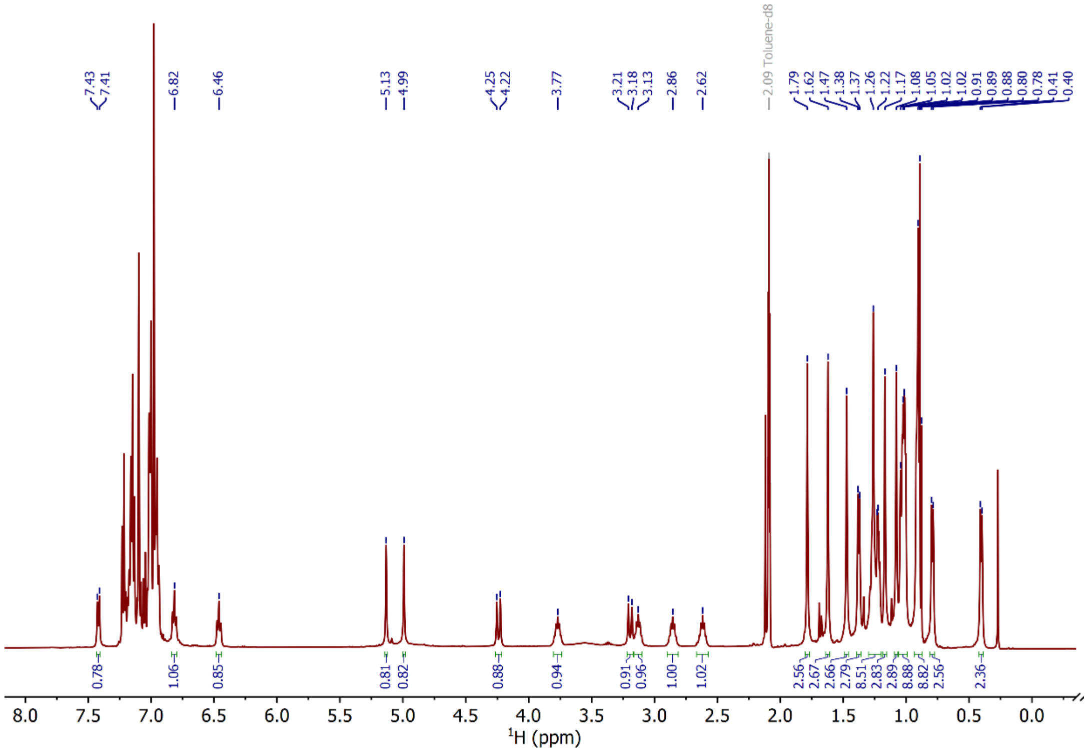

Figure S16. 500.3 MHz ^{1}H NMR spectrum of complex **3** in toluene-d_8.

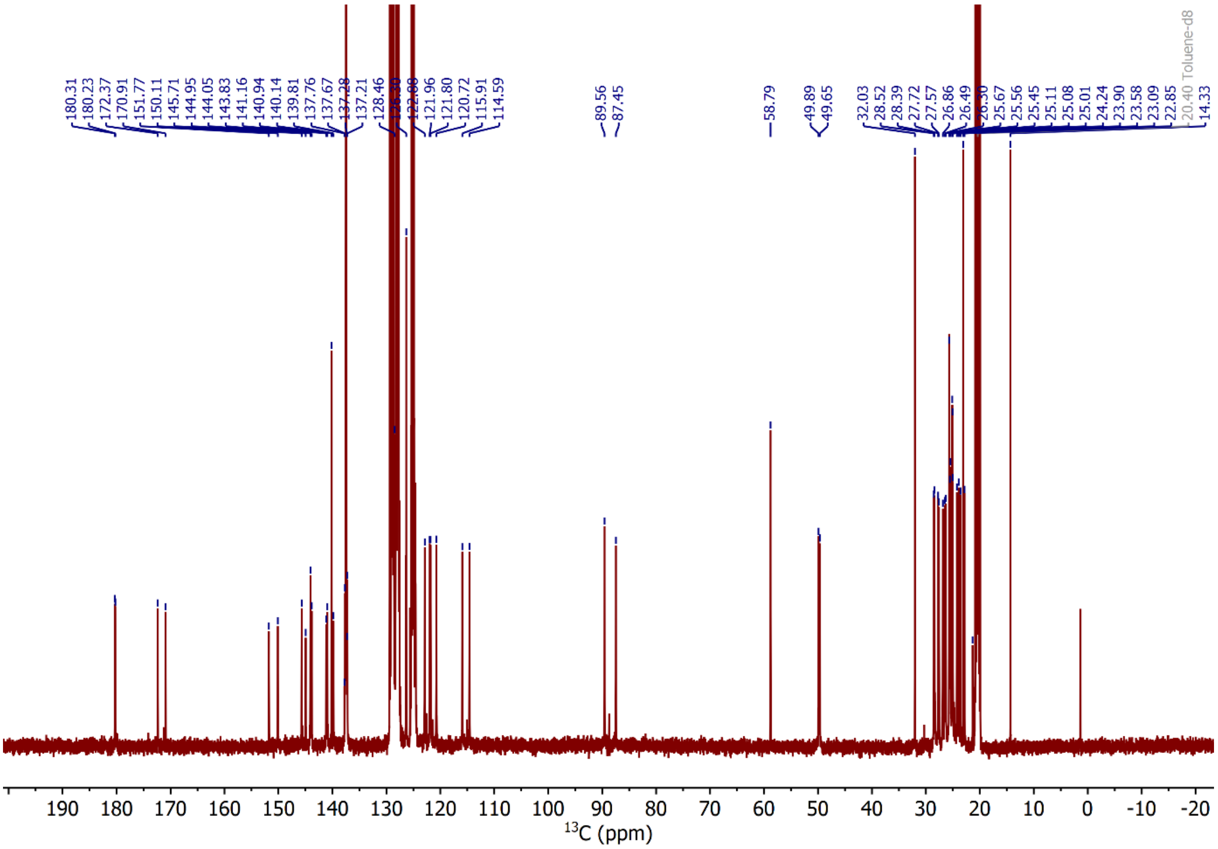

Figure S17. 125.8 MHz ^{13}C{^{1}H} NMR spectrum of complex **3** in toluene-d_8.

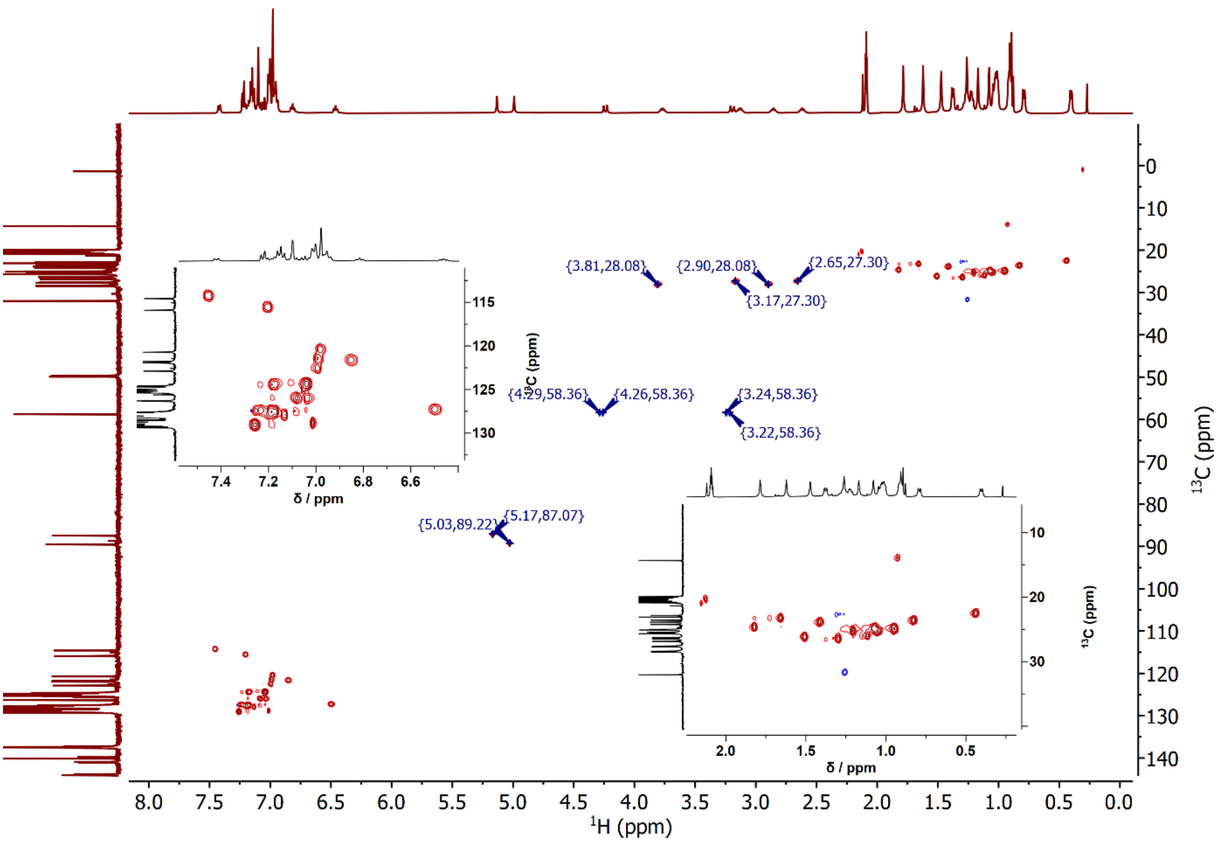

Figure S18. 600.3/150.9 MHz $^{1}H,^{13}C$ HSQC spectrum of complex **3** in toluene-d_8.

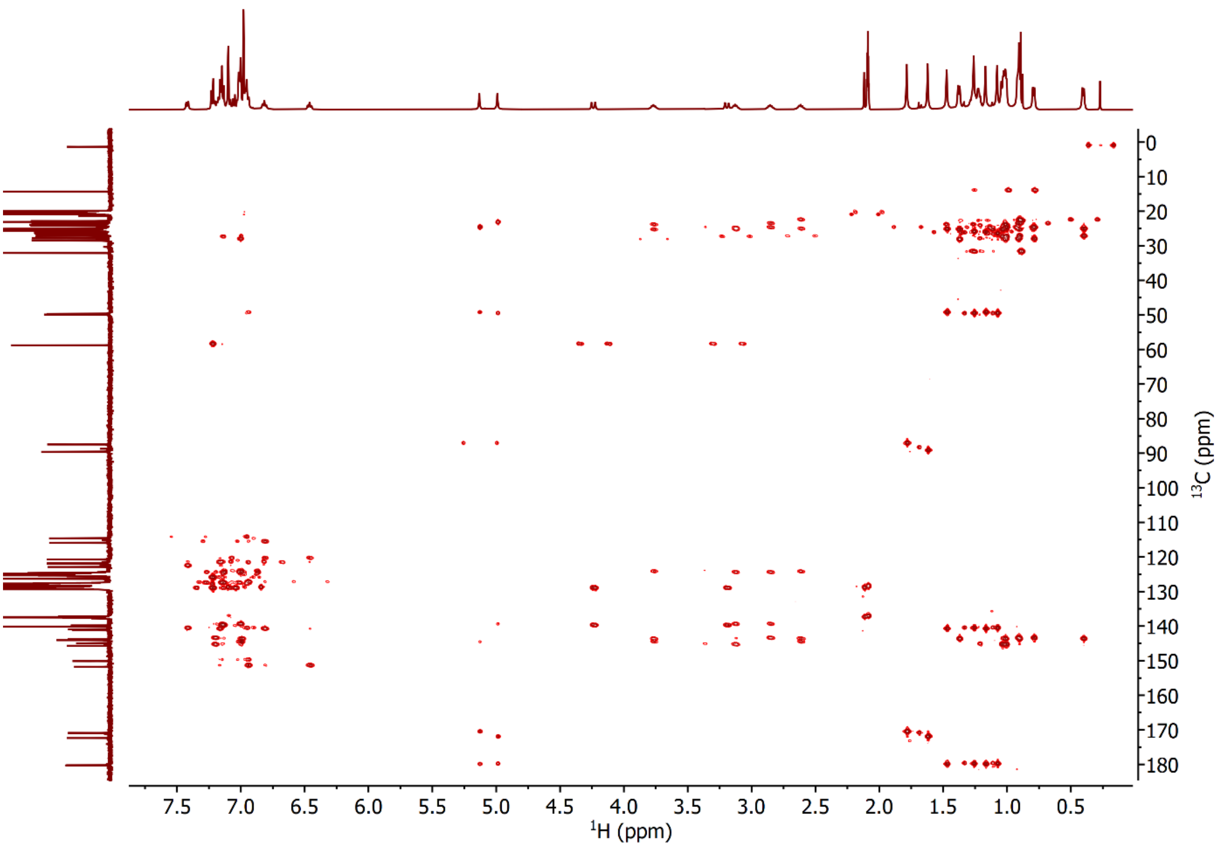

Figure S19. 600.3/150.9 MHz $^{1}H,^{13}C$ HMBC spectrum of complex **3** in toluene-d_8.

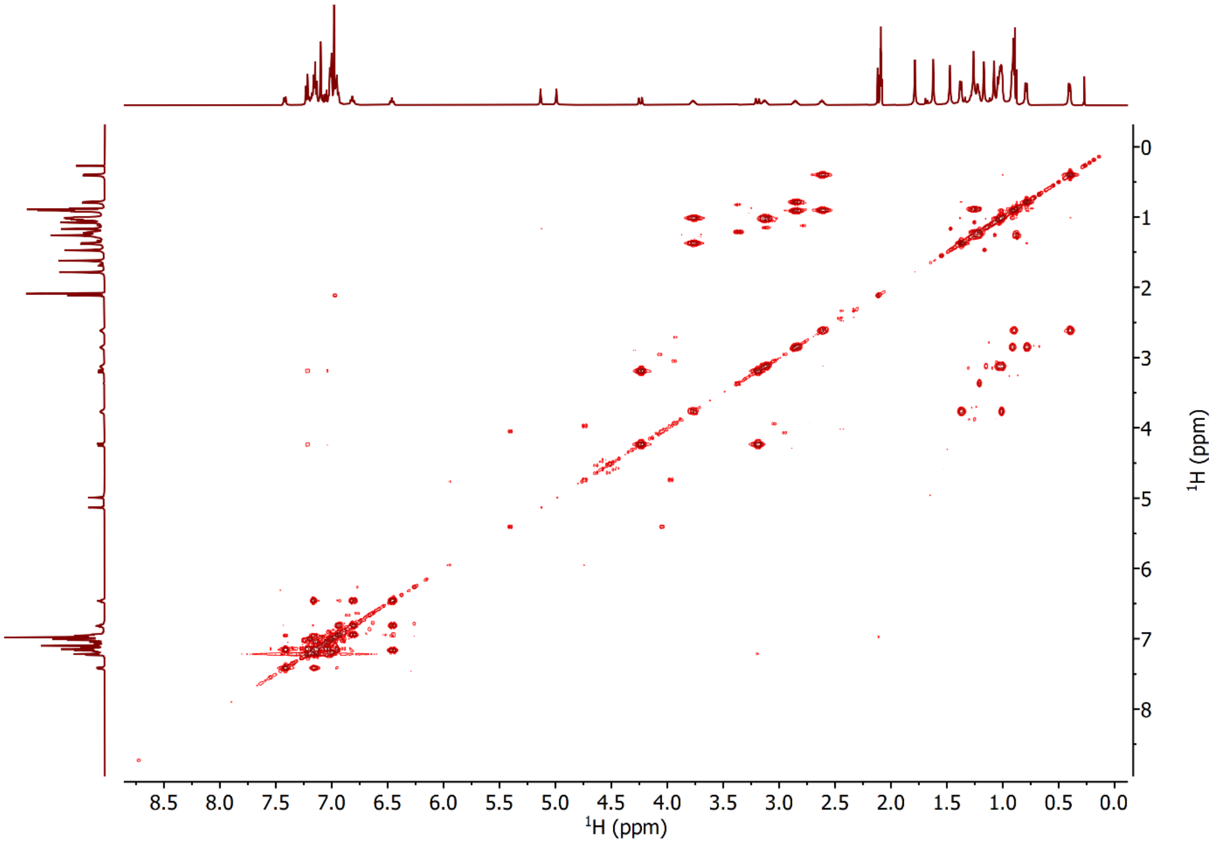

Figure S20: 600.3 MHz ^{1}H COSY spectrum of complex **3** in toluene-d_8.

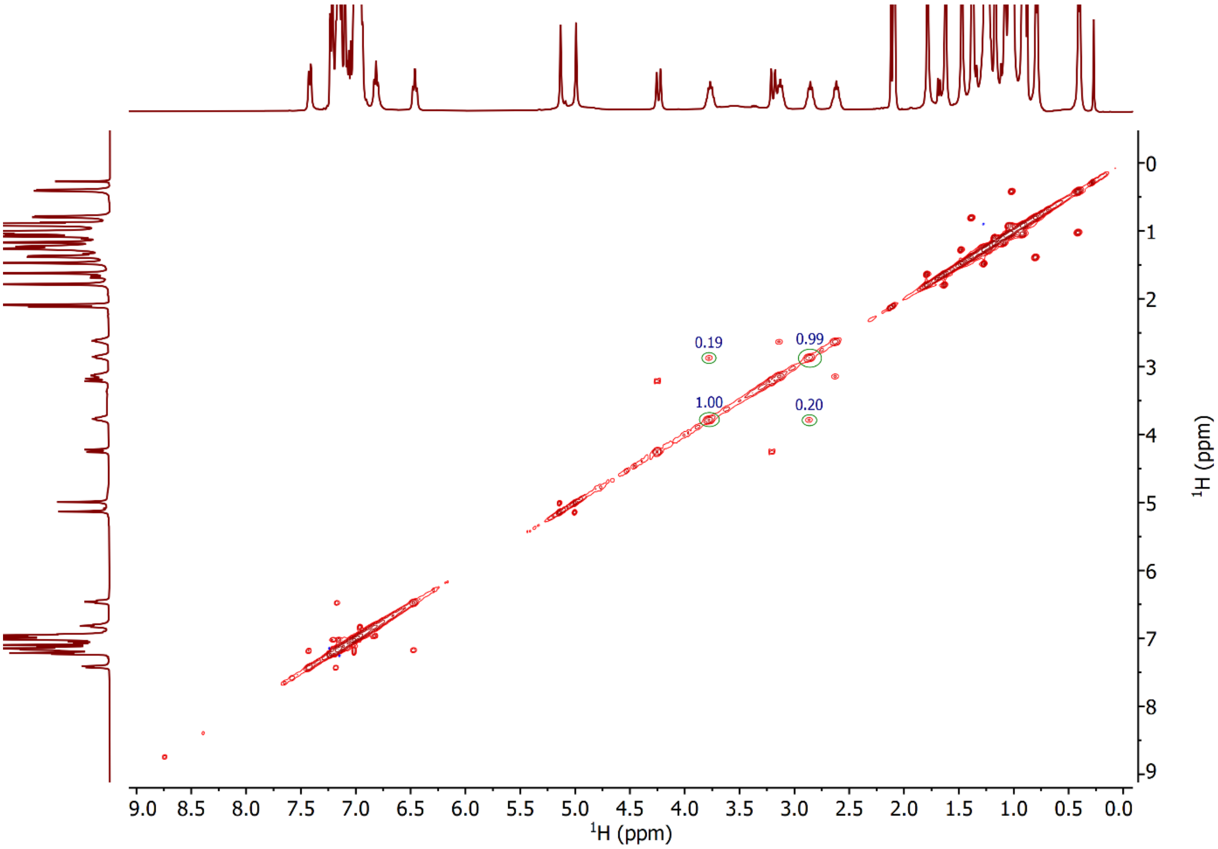

Figure S21. 400.3 MHz ^{1}H EXSY spectrum of complex **3** in toluene-d_8.

Complex 4

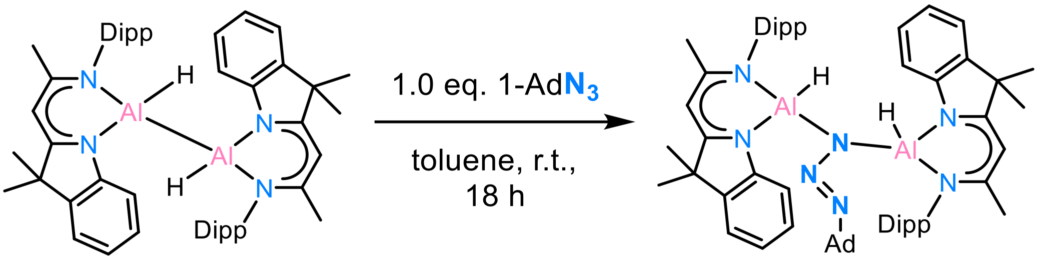

I

4, 55%

Dialane **I** (0.030 g, 0.038 mmol, 1.0 eq.) and 1-azidoadamantane (6.86 mg, 0.038 mmol, 1.0 eq) were added dropwise to 0.5 mL toluene-d_8 and transferred to a JY NMR tube. The reaction was monitored by ^{1}H NMR spectroscopy. After 18 hours the ^{1}H NMR confirmed the reaction to be completed. The solvent was removed *in vacuo*. *n*-Hexane was added and the sample was dried to remove all volatiles. Crystals suitable for SC-XRD analysis were grown from a toluene/*n*-hexane (1:1) mixture at -30 °C over night. The yellow crystals were isolated and dried *in vacuo* (20.20 mg, 0.021 mmol, 55%). LIFDI (positive mode, toluene): Calcd. for $C_{60}H_{79}Al_2N_7$: 951.6; Found: 951.4. Elemental analysis: Calcd: C 75.68, H 8.36, N 10.30, found: C 75.83, H 8.11, N 9.99. The detailed NMR discussion can be found in the article.

Complex 5

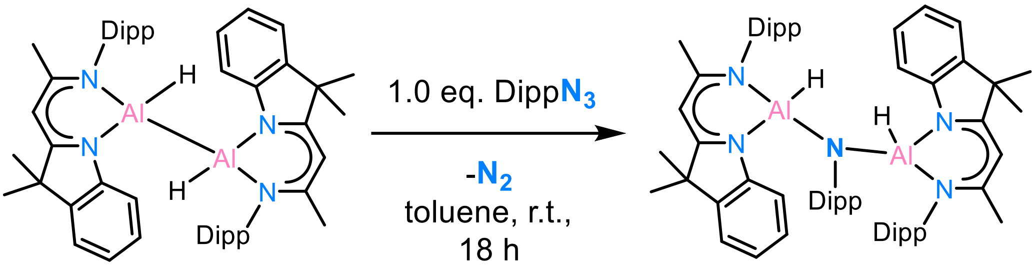

I

5, 70%

Dialane **I** (0.025 g, 0.032 mmol, 1.0 eq.) and 2,6-diisopropylphenyl azide (6.60 mg, 0.032 mmol, 1.0 eq) were added into 0.5 mL toluene-d_8 and transferred to a JY NMR tube. The reaction was monitored by ^{1}H NMR spectroscopy. After 18 hours ^{1}H NMR confirmed the reaction to be completed. The solvent was removed *in vacuo*. *n*-Hexane was added and the sample was dried to remove all volatiles. Crystals suitable for SC-XRD experiment were grown in benzene at room temperature over one day. The yellow-brown crystals were isolated and dried *in vacuo* (18.01 mg, 0.023 mmol, 70%). LIFDI (positive mode, toluene): Calcd. for $C_{62}H_{81}Al_2N_5$: 949.6; Found: 949.4. Elemental analysis: Calcd: C 78.36, H 8.59, N 7.37, found: C 77.98, H 8.55, N 7.01. ^{1}H NMR (298 K, toluene-d_8, 500.3 MHz): δ (ppm): 7.32 (d, 1H, 27-H, *J* = 7.83 Hz), 7.29 (dd, 1H, 41-H, *J* = 7.76, 1.60 Hz), 7.22 (t, 1H, 42-H, J = 7.71 Hz), 7.09 (m, 1H, 28-H), 6.97-7.02 (m, 5H, 54-H, 55-H, 2-H, 5-H, 16-H), 6.92-6.94 (m, 2H, 18-H, 43-H), 6.80-6.88 (m, 5H, 53-H, 17-H, 4-H, 29-H, 30-H), 6.58 (td, 1H, 3-H, *J* = 7.64, 1.27 Hz), 5.33 (s, 1H, 11-H), 4.82 (s, 1H, 36-H), 4.31 (hept, 1H, 57-H, *J* = 6.67 Hz), 3.89 (hept, 1H, 45-H, *J* = 6.67 Hz), 3.60 (hept, 1H, 60-H, *J* = 6.62 Hz), 2.59 (hept, 1H, 20-H, *J* = 6.81 Hz), 2.45 (hept, 1H, 48-H, *J* = 6.81 Hz), 2.23 (hept, 1H, 23-H, *J* = 6.67 Hz), 1.89 (d, 3H, 47-H, *J* = 6.63 Hz), 1.64 (s, 3H, 38-H), 1.63 (s, 3H, 8-H), 1.58 (s, 3H, 13-H), 1.38 (d, 3H, 58-H, *J* = 6.83 Hz), 1.37 (d, 3H, 46-H, *J* = 6.83 Hz), 1.27 (s, 3H, 9-H), 1.15-1.19 (m, 9H, 59-H, 61-H, 22-H), 0.95 (s, 3H, 33-H), 0.94 (d, 3H, 25-H, *J* = 7.05 Hz), 0.81 (d, 3H, 21-H, J = 6.67 Hz), 0.79 (d, 3H, 24-H, *J* = 6.67 Hz), 0.77 (d, 3H, 50-H, *J* = 6.73 Hz), 0.68 (s, 3H, 34-H), 0.56 (d, 3H, 49-H, *J* = 6.84 Hz), 0.30 (d, 3H, 62-H, *J* = 6.68 Hz). ^{13}C NMR (298 K, toluene-d_8, 125.8 MHz): δ (ppm) = 180.3 (1C, 10-C), 180.2 (1C, 35-C), 172.9 (1C, 12-C), 172.0 (1C, 37-C), 151.4 (1C, 1-C, 150.6 (1C, 26-C), 149.2 (1C, 51-C), 145.1 (1C, 19-C), 145.0 (1C, 52-C), 144.9 (1C, 40-C), 144.7 (2C, 44-C, 56-C), 144.6 (1C, 15-C), 143.7 (1C, 39-C), 143.1 (1C, 14-C), 141.3 (1C, 31-C), 140.1 (1C, 6-C), 127.9 (1C, 3-C), 127.3 (1C, 42-C), 127.1 (1C, 28-C), 125.4 (1C, 54-C), 124.9 (1C, 43-C), 124.5 (1C, 18-C), 124.4 (1C, 41-C), 124.0 (1C, 16-C), 123.6 (1C, 55-C), 123.0 (1C, 53-C), 122.7 (1C, 29-C), 122.0 (1C, 4-C), 121.8 (1C, 30-C), 120.9 (1C, 5-C), 120.3 (1C, 17-C), 116.0 (1C, 2-C), 114.1 (1C, 27-C), 94.4 (2C, 11-C, 36-C), 49.7 (1C, 7-C), 49.1 (1C, 32-C), 28.6 (1C, 57-C), 28.5 (1C, 45-C), 28.4 (2C, 48-C, 59-C), 28.3 (1C, 23-C), 28.1 (1C, 20-C), 27.5 (2C, 60-C, 61-C), 27.1 (2C, 33-C, 47-C), 27.0 (1C, 9-C), 26.3 (1C, 25-C), 26.1 (1C, 46-C), 25.8 (1C, 8-C), 25.2

(1C, 34-C), 24.9 (2C, 13-C, 58-C), 24.7 (2C, 24-C, 49-C), 24.6 (1C, 22-C), 24.4 (1C, 38-C), 24.3 (1C, 21-C), 23.9 (1C, 62-C), 23.2 (1C, 50-C). ^{15}N NMR (298 K, toluene-d_8, 50.7 MHz): δ (ppm) = −195.5 (1N, 4-N), −202.3 (2N, 1-N, 2-N), −213.5 (1N, 3-N).

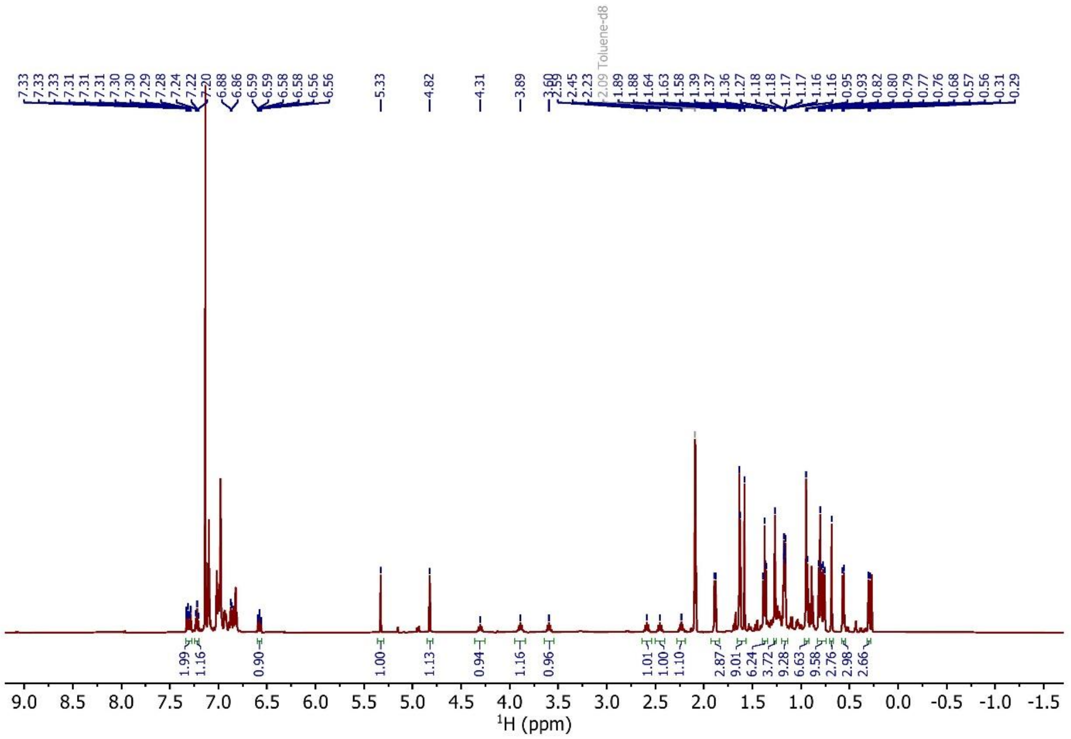

Figure S22. 500.3 MHz ^{1}H NMR spectrum of complex **5** in toluene-d_8.

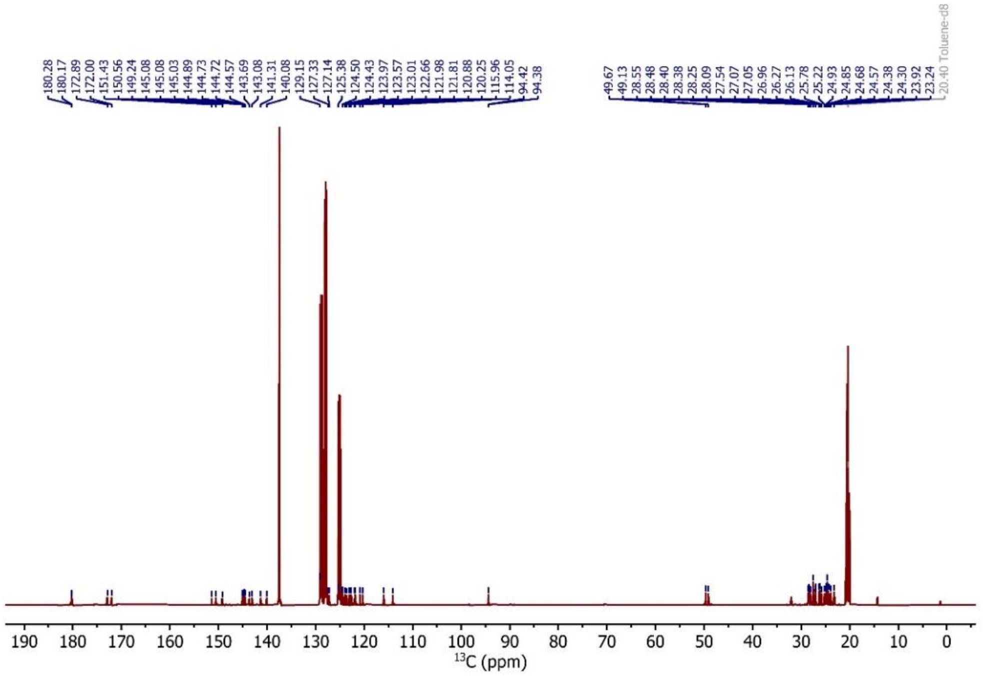

Figure S23. 125.8 MHz ^{13}C{^{1}H} NMR spectrum of complex **5** in toluene-d_8.

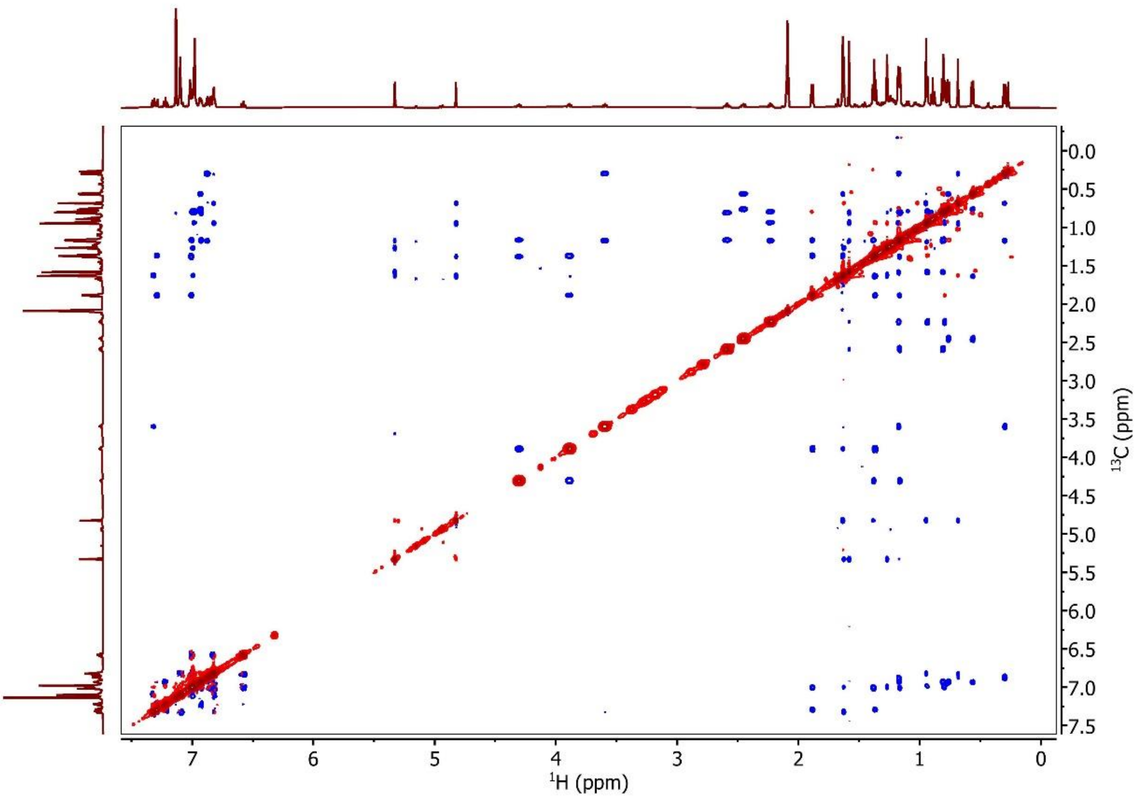

Figure S24. 500.3 MHz ^{1}H NOESY spectrum of complex **5** in toluene-d_8.

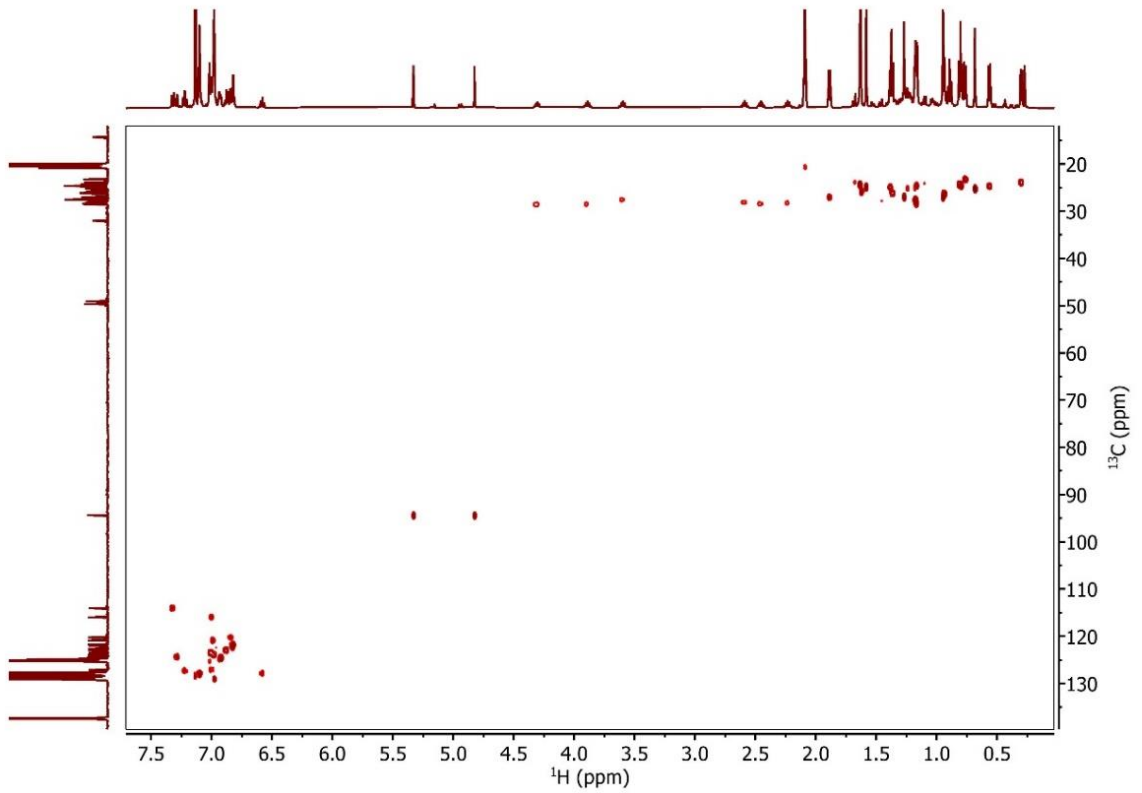

Figure S25. 500.3/125.8 MHz ^{1}H,^{13}C HSQC spectrum of complex **5** in toluene-d_8.

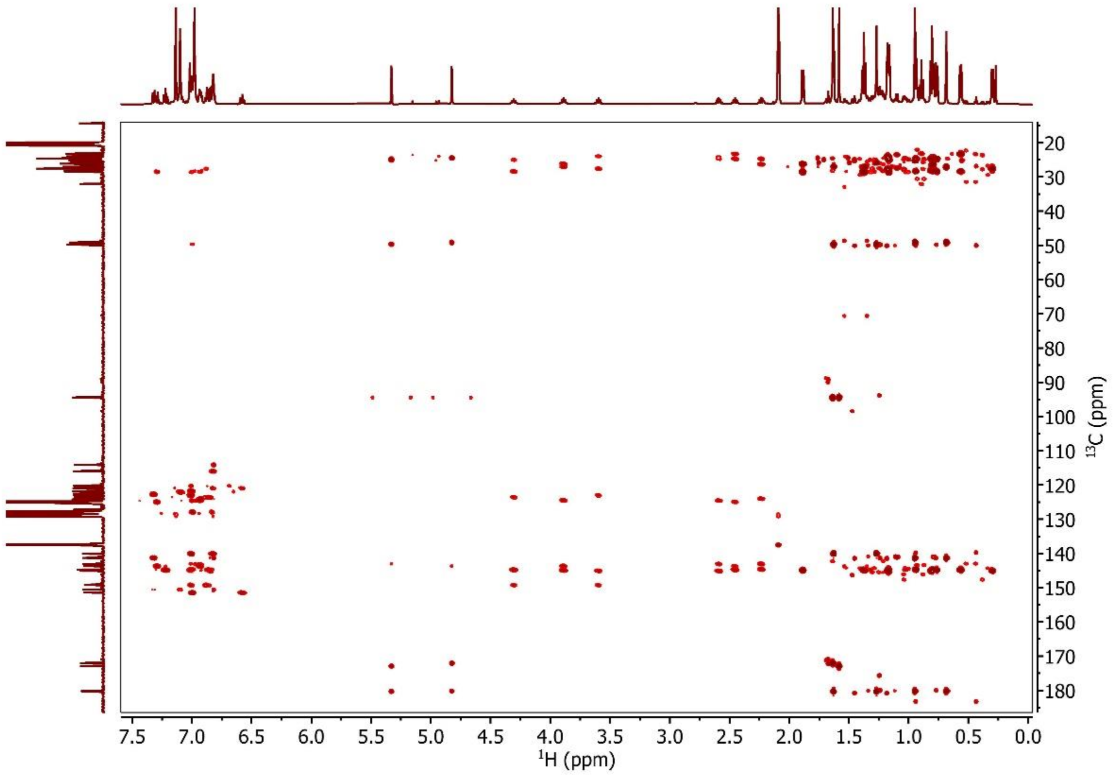

Figure S26. 500.3/125.8 MHz ^{1}H,^{13}C HMBC spectrum of complex **5** in toluene-d_8.

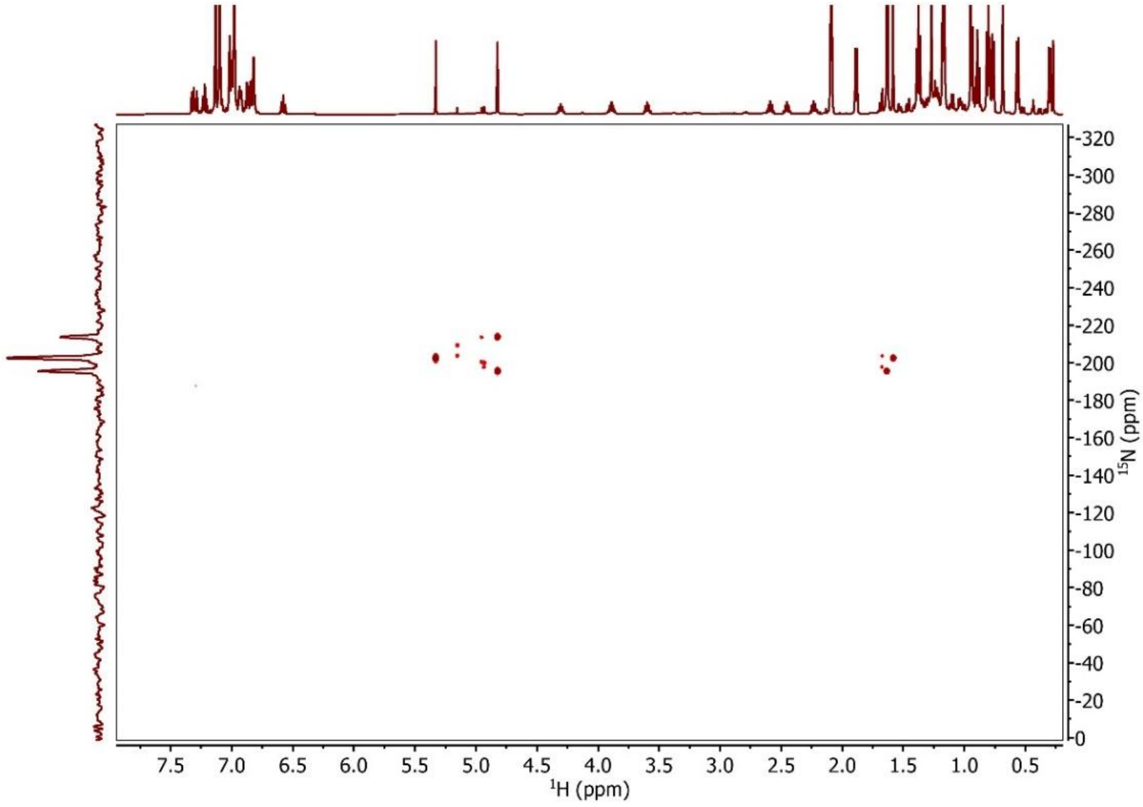

Figure S27. 500.3/50.7 MHz ^{1}H,^{15}N HMBC spectrum of complex **5** in toluene-d_8.

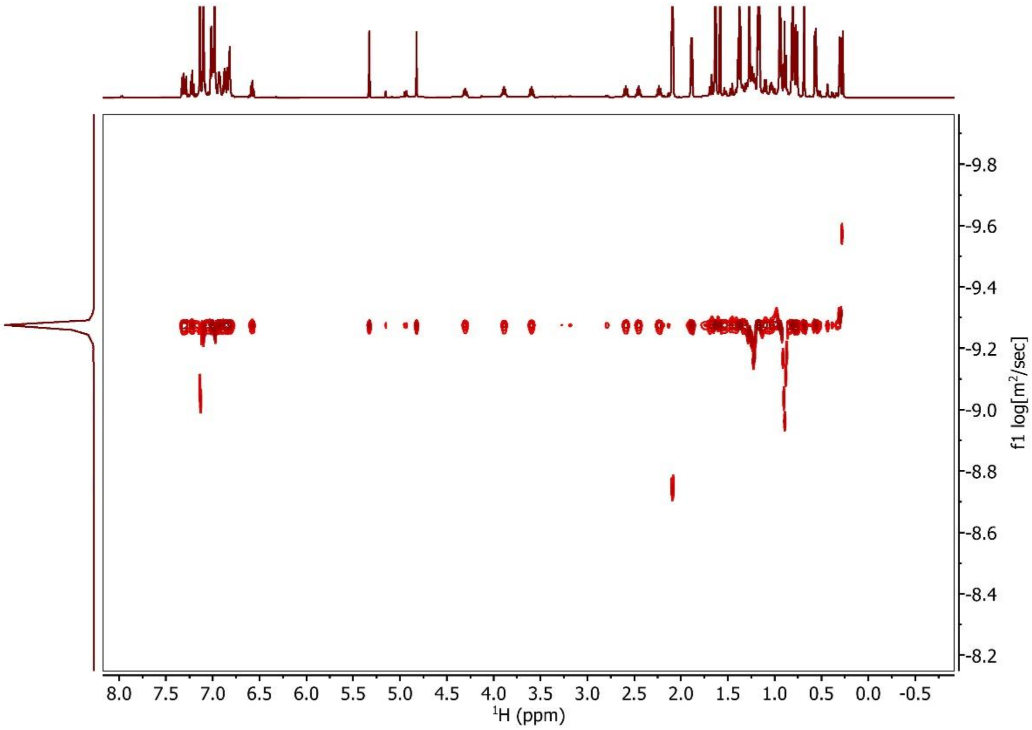

Figure S28. 500.3 MHz ^{1}H DOSY spectrum of complex **5** in toluene-d_8.

Photophysical Data

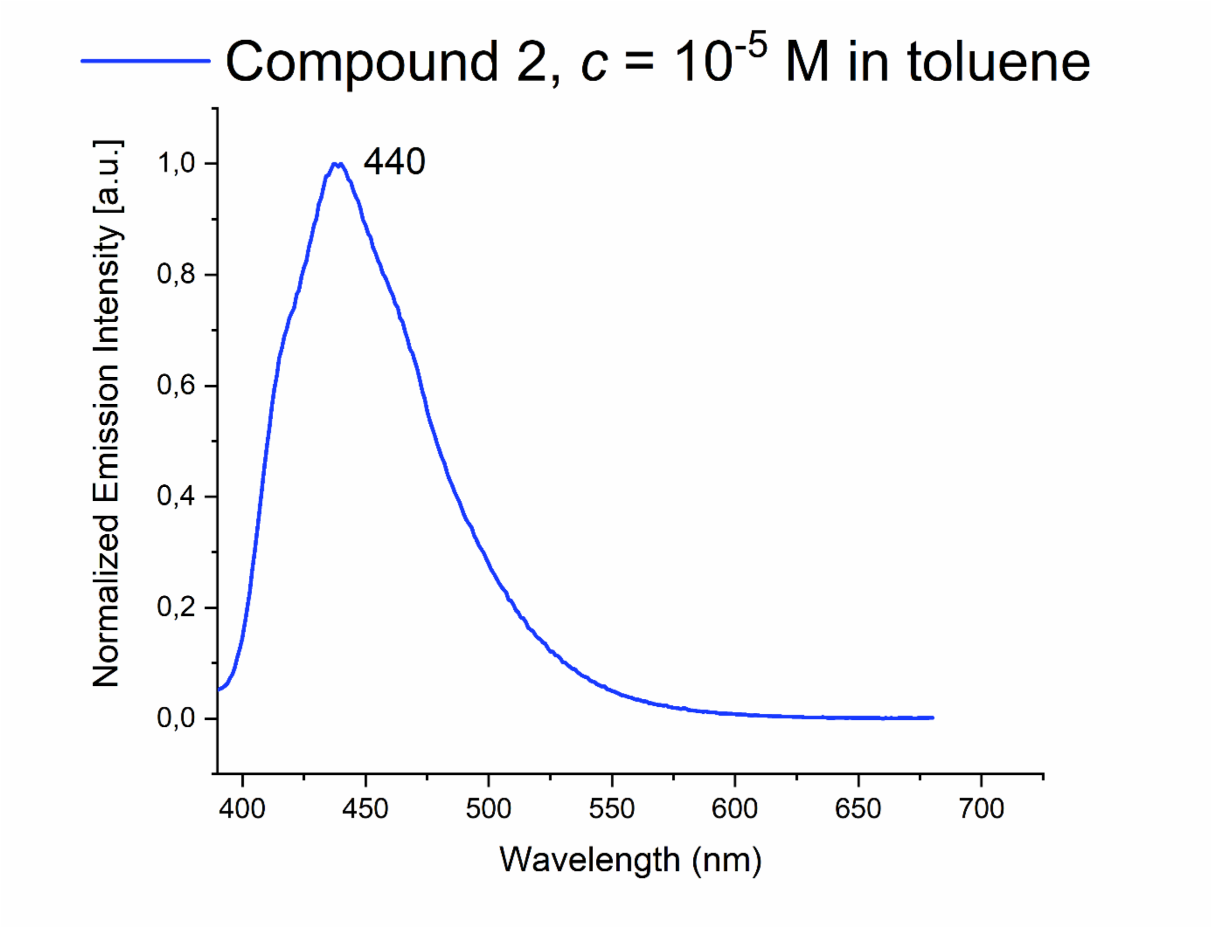

Figure S29: Emission spectra of complex **2** in toluene (10^{-5} M).

Crystallographic data

General Data Acquisition and Processing

The diffraction data were collected using Mo Kα radiation and a Bruker Photon III C7 Detector. The data were integrated with *SAINT*.[1] A multi-scan absorption correction was applied using *SADABS*[2] or *TWINABS*[3]. The structures were solved by *SHELXT*[4] and refined on F^2 using *SHELXL*[5] in the graphical user interface *ShelXle*[6]. All hydrogen atoms bond to carbon atoms were placed according to geometrical criteria and refined with a riding model except those mentioned below.

Table S1: Crystal data and structure refinement for complexes **1**, **2**, **3** at 100(2)K.

Compound	**1**	**2**	**3**
CCDC	2422952	2422953	2314164
Empirical Formula	$C_{64}H_{78}Al_2N_{10}$	$C_{31}H_{49}AlN_6Si_2$, 0.5(C_7H_8)	$C_{57}H_{71}Al_2N_7$, 0.5(C_6H_{14})
Formula weight	1041.32	634.99	951.25
T [K]	100(2)	100(2)	100(2)
λ [Å]	0.71073	0.71073	0.71073
Crystal system	Triclinic	Monoclinic	Monoclinic
Space group	$P\bar{1}$	$C2/c$	$P2_1$/c
a [Å]	11.071(2)	28.496(3)	15.158(2)
b [Å]	11.439(2)	17.773(2)	15.994(2)
c [Å]	26.393(3)	19.542(2)	23.383(3)
α [°]	100.37(3)	-	-
β [°]	91.88(2)	128.56(2)	103.53(2)
γ [°]	116.23(2)	-	-
V [Å^3]	2925.3(10)	7739(2)	5511.6(13)
Z	2	8	4
μ [mm^{-1}]	0.098	0.144	0.097
F(000)	1116	2744	2052
Crystal size [mm]	0.33x0.214x0.177	0.195x0.168x0.115	0.340x0.248x0.182
Θ range [°]	2.033 to 25.023	1.466 to 26.438	1.879 to 26.444
Reflections collected	46646	218671	339316
Independent reflections	10244	7938	11322
R_{int}	0.0434	0.0632	0.0968
Data/restraints/parameters	10244/1221/838	7938/122/439	11322/0/619
GooF	1.030	1.048	1.034
$R1$ [$I > 2\sigma(I)$]	0.0359	0.0387	0.0461
$wR2$ [all data]	0.0878	0.0973	0.1022
ρ_{max}/ρ_{min} [e A^{-3}]	0.292/-0.233	0.342/-0.280	0.309/-0.296
Shape and color	yellow block	yellow block	yellow block

$^aR1 = \Sigma||F_o| - |F_c||/\Sigma|F_o|$. $^bwR_2 = [\Sigma w(F_o^2 - F_c^2)^2/\Sigma(F_o^2)^2]^{1/2}$

Table S2: Crystal data and structure refinement for complexes **4**, **5** at 100(2)K.

Compound	**4**	**5**
CCDC	2422954	2422955
Empirical Formula	$C_{60}H_{79}Al_2N_7$, C_7H_8	$C_{62}H_{81}Al_2N_5$, 1.5(C_6H_6)
Formula weight	1044.39	1067.43
T [K]	100(2)	100(2)
λ [Å]	0.71073	0.71073
Crystal system	Monoclinic	Monoclinic
Space group	$P2_1/c$	$P2_1/n$
a [Å]	12.206(2)	12.855(2)
b [Å]	21.963(3)	27.485(3)
c [Å]	23.341(3)	18.298(2)
β [°]	94.19(2)°	100.18(2)
V [Å^3]	6240.5(16)	6363.3(14)
Z	4	4
μ [mm^{-1}]	0.091	0.090
F(000)	2256	2308
Crystal size [mm]	0.419x0.237x0.223	0.504x0.252x0.158
Θ range [°]	1.673 to 26.435	2.188 to 26.425
Reflections collected	468696	380111
Independent reflections	12734	13006
R_{int}	0.0636	0.0655
Data/restraints/parameters	12734/767/744	13006/ 2 / 657
GooF	1.039	1.038
$R1$ [$I > 2\sigma(I)$]	0.0399	0.0376
$wR2$ [all data]	0.1059	0.0990
ρ_{max}/ρ_{min} [e A^{-3}]	0.527/-0.317	0.271/-0.253
Shape and color	yellow block	yellow block

$^aR1 = \Sigma||F_o| - |F_c||/\Sigma|F_o|$. $^bwR_2 = [\Sigma w(F_o^2 - F_c^2)^2/\Sigma(F_o^2)^2]^{1/2}$

Crystal structure of 1

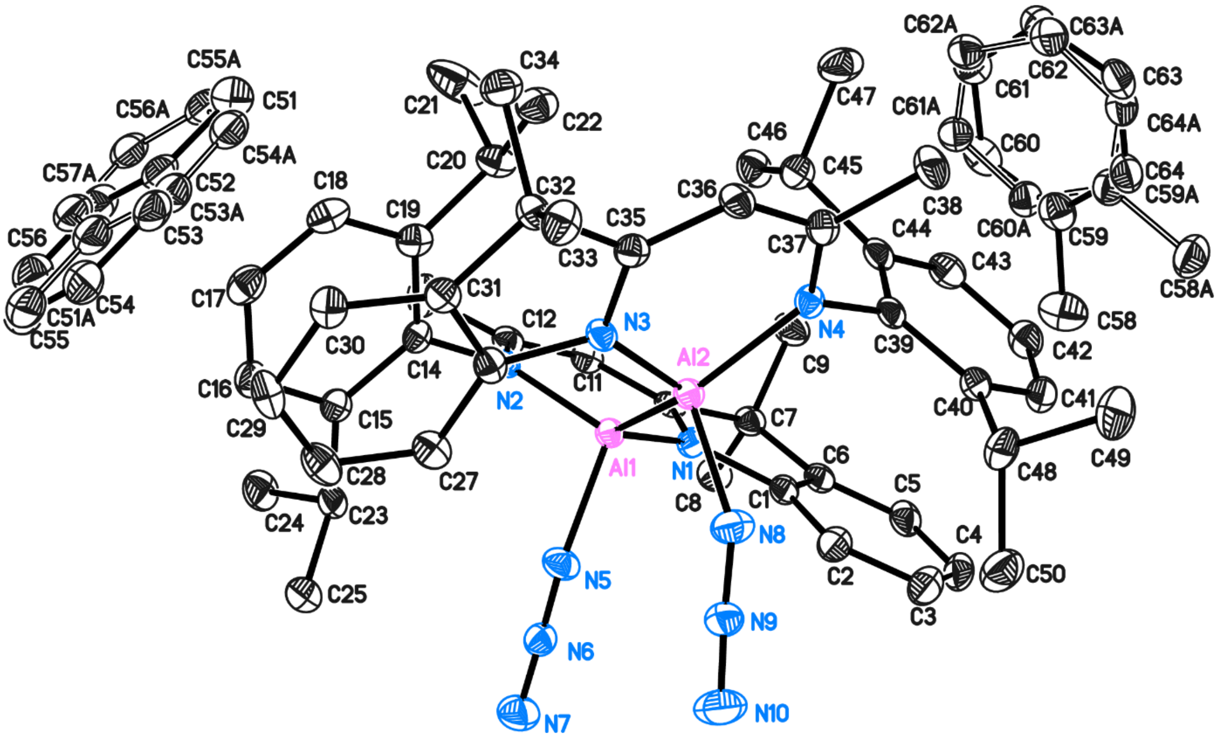

Figure S30: Asymmetric unit of **1** with thermal ellipsoids shown at 50% probability level. Hydrogen atoms are omitted for clarity. The hydrogen atoms bond to C11 and C36 were refined freely. Both C-H distances were restrained to be similar. Both toluene molecule are disordered over two positions and were refined with distance restraints and restraints for anisotropic displacement parameters. The occupancies of the minor positions refined to 0.212(4) and 0.037(2), respectively. The data were measured on a non-merohedrally twinned crystal with the twin law 0.955 -0.090 0.020 -0.978 -0.955 -0.010 0 0 -1. The fractional contribution of the minor component refined to 0.4496(8).

Table S3: Bond lengths [Å] and angles [°] for **1**.

Al(1)-N(5)	1.8669(18)	N(3)-C(26)	1.424(2)
Al(1)-N(1)	1.9043(16)	N(4)-C(37)	1.343(2)
Al(1)-N(2)	1.9310(16)	N(4)-C(39)	1.457(2)
Al(1)-Al(2)	2.6008(12)	N(5)-N(6)	1.203(2)
Al(2)-N(8)	1.8686(18)	N(6)-N(7)	1.150(2)
Al(2)-N(3)	1.9009(16)	N(8)-N(9)	1.210(2)
Al(2)-N(4)	1.9225(17)	N(9)-N(10)	1.142(2)
N(1)-C(10)	1.357(2)	C(1)-C(2)	1.383(3)
N(1)-C(1)	1.423(2)	C(1)-C(6)	1.397(3)
N(2)-C(12)	1.339(2)	C(2)-C(3)	1.399(3)
N(2)-C(14)	1.455(2)	C(3)-C(4)	1.384(3)
N(3)-C(35)	1.345(2)	C(4)-C(5)	1.398(3)

C(5)-C(6)	1.385(3)	C(43)-C(44)	1.395(3)
C(6)-C(7)	1.509(3)	C(44)-C(45)	1.523(3)
C(7)-C(9)	1.536(3)	C(45)-C(46)	1.530(3)
C(7)-C(10)	1.536(2)	C(45)-C(47)	1.532(3)
C(7)-C(8)	1.543(3)	C(48)-C(50)	1.531(3)
C(10)-C(11)	1.379(3)	C(48)-C(49)	1.538(3)
C(11)-H(11)	0.955(18)	C(51)-C(52)	1.506(3)
C(11)-C(12)	1.409(3)	C(52)-C(53)	1.385(3)
C(12)-C(13)	1.504(3)	C(52)-C(57)	1.390(3)
C(14)-C(15)	1.411(3)	C(53)-C(54)	1.379(4)
C(14)-C(19)	1.412(3)	C(54)-C(55)	1.395(4)
C(15)-C(16)	1.391(3)	C(55)-C(56)	1.371(4)
C(15)-C(23)	1.523(3)	C(56)-C(57)	1.377(4)
C(16)-C(17)	1.386(3)	C(51A)-C(52A)	1.504(15)
C(17)-C(18)	1.379(3)	C(52A)-C(53A)	1.381(14)
C(18)-C(19)	1.396(3)	C(52A)-C(57A)	1.388(14)
C(19)-C(20)	1.520(3)	C(53A)-C(54A)	1.383(14)
C(20)-C(22)	1.527(3)	C(54A)-C(55A)	1.386(14)
C(20)-C(21)	1.529(3)	C(55A)-C(56A)	1.382(14)
C(23)-C(25)	1.528(3)	C(56A)-C(57A)	1.384(14)
C(23)-C(24)	1.538(3)	C(58)-C(59)	1.501(4)
C(26)-C(27)	1.387(3)	C(59)-C(60)	1.387(5)
C(26)-C(31)	1.396(3)	C(59)-C(64)	1.391(4)
C(27)-C(28)	1.395(3)	C(60)-C(61)	1.384(5)
C(28)-C(29)	1.390(3)	C(61)-C(62)	1.386(5)
C(29)-C(30)	1.400(3)	C(62)-C(63)	1.391(5)
C(30)-C(31)	1.381(3)	C(63)-C(64)	1.386(4)
C(31)-C(32)	1.509(3)	C(58A)-C(59A)	1.502(11)
C(32)-C(35)	1.534(2)	C(59A)-C(64A)	1.381(12)
C(32)-C(34)	1.536(3)	C(59A)-C(60A)	1.384(11)
C(32)-C(33)	1.542(2)	C(60A)-C(61A)	1.369(11)
C(35)-C(36)	1.387(3)	C(61A)-C(62A)	1.383(12)
C(36)-H(36)	0.957(18)	C(62A)-C(63A)	1.382(12)
C(36)-C(37)	1.401(3)	C(63A)-C(64A)	1.385(12)
C(37)-C(38)	1.509(3)		
C(39)-C(40)	1.407(3)	N(5)-Al(1)-N(1)	107.24(7)
C(39)-C(44)	1.416(3)	N(5)-Al(1)-N(2)	106.86(7)
C(40)-C(41)	1.396(3)	N(1)-Al(1)-N(2)	94.25(7)
C(40)-C(48)	1.522(3)	N(5)-Al(1)-Al(2)	97.21(6)
C(41)-C(42)	1.376(3)	N(1)-Al(1)-Al(2)	121.37(5)
C(42)-C(43)	1.389(3)	N(2)-Al(1)-Al(2)	128.42(5)

N(8)-Al(2)-N(3)	101.88(7)	H(11)-C(11)-C(10)	117.7(13)
N(8)-Al(2)-N(4)	103.48(8)	H(11)-C(11)-C(12)	116.2(13)
N(3)-Al(2)-N(4)	94.74(7)	C(10)-C(11)-C(12)	126.14(17)
N(8)-Al(2)-Al(1)	106.60(6)	N(2)-C(12)-C(11)	123.02(17)
N(3)-Al(2)-Al(1)	120.85(5)	N(2)-C(12)-C(13)	121.15(16)
N(4)-Al(2)-Al(1)	125.96(6)	C(11)-C(12)-C(13)	115.83(16)
C(10)-N(1)-C(1)	108.19(14)	C(15)-C(14)-C(19)	121.22(16)
C(10)-N(1)-Al(1)	125.50(12)	C(15)-C(14)-N(2)	120.14(16)
C(1)-N(1)-Al(1)	126.30(12)	C(19)-C(14)-N(2)	118.61(16)
C(12)-N(2)-C(14)	119.18(15)	C(16)-C(15)-C(14)	117.97(17)
C(12)-N(2)-Al(1)	126.31(12)	C(16)-C(15)-C(23)	118.72(17)
C(14)-N(2)-Al(1)	114.51(11)	C(14)-C(15)-C(23)	123.21(16)
C(35)-N(3)-C(26)	108.53(14)	C(17)-C(16)-C(15)	121.51(18)
C(35)-N(3)-Al(2)	124.99(12)	C(18)-C(17)-C(16)	119.85(17)
C(26)-N(3)-Al(2)	126.43(12)	C(17)-C(18)-C(19)	121.36(18)
C(37)-N(4)-C(39)	119.61(15)	C(18)-C(19)-C(14)	118.02(17)
C(37)-N(4)-Al(2)	126.13(12)	C(18)-C(19)-C(20)	119.90(17)
C(39)-N(4)-Al(2)	114.26(11)	C(14)-C(19)-C(20)	121.97(16)
N(6)-N(5)-Al(1)	140.59(14)	C(19)-C(20)-C(22)	113.11(16)
N(7)-N(6)-N(5)	175.91(19)	C(19)-C(20)-C(21)	110.91(17)
N(9)-N(8)-Al(2)	135.60(14)	C(22)-C(20)-C(21)	108.73(17)
N(10)-N(9)-N(8)	176.2(2)	C(15)-C(23)-C(25)	112.08(15)
C(2)-C(1)-C(6)	122.25(17)	C(15)-C(23)-C(24)	110.58(16)
C(2)-C(1)-N(1)	126.93(16)	C(25)-C(23)-C(24)	109.86(17)
C(6)-C(1)-N(1)	110.80(16)	C(27)-C(26)-C(31)	121.94(16)
C(1)-C(2)-C(3)	117.23(18)	C(27)-C(26)-N(3)	127.49(16)
C(4)-C(3)-C(2)	121.3(2)	C(31)-C(26)-N(3)	110.55(16)
C(3)-C(4)-C(5)	120.67(18)	C(26)-C(27)-C(28)	117.19(17)
C(6)-C(5)-C(4)	118.72(18)	C(29)-C(28)-C(27)	121.40(18)
C(5)-C(6)-C(1)	119.81(18)	C(28)-C(29)-C(30)	120.57(17)
C(5)-C(6)-C(7)	131.30(17)	C(31)-C(30)-C(29)	118.42(17)
C(1)-C(6)-C(7)	108.88(15)	C(30)-C(31)-C(26)	120.37(17)
C(6)-C(7)-C(9)	112.05(16)	C(30)-C(31)-C(32)	130.94(17)
C(6)-C(7)-C(10)	100.63(14)	C(26)-C(31)-C(32)	108.70(15)
C(9)-C(7)-C(10)	111.34(15)	C(31)-C(32)-C(35)	100.67(14)
C(6)-C(7)-C(8)	112.56(16)	C(31)-C(32)-C(34)	112.60(16)
C(9)-C(7)-C(8)	108.83(16)	C(35)-C(32)-C(34)	110.43(14)
C(10)-C(7)-C(8)	111.30(15)	C(31)-C(32)-C(33)	111.85(15)
N(1)-C(10)-C(11)	124.59(16)	C(35)-C(32)-C(33)	111.82(15)
N(1)-C(10)-C(7)	111.44(15)	C(34)-C(32)-C(33)	109.29(15)
C(11)-C(10)-C(7)	123.98(16)	N(3)-C(35)-C(36)	124.86(16)

N(3)-C(35)-C(32)	111.48(15)	C(54)-C(53)-C(52)	121.3(2)
C(36)-C(35)-C(32)	123.66(17)	C(53)-C(54)-C(55)	119.7(2)
H(36)-C(36)-C(35)	115.9(12)	C(56)-C(55)-C(54)	119.4(2)
H(36)-C(36)-C(37)	117.7(12)	C(55)-C(56)-C(57)	120.5(2)
C(35)-C(36)-C(37)	126.33(17)	C(56)-C(57)-C(52)	121.0(2)
N(4)-C(37)-C(36)	122.55(17)	C(53A)-C(52A)-C(57A)	120.5(16)
N(4)-C(37)-C(38)	120.73(16)	C(53A)-C(52A)-C(51A)	121.4(19)
C(36)-C(37)-C(38)	116.71(16)	C(57A)-C(52A)-C(51A)	118.0(19)
C(40)-C(39)-C(44)	121.46(16)	C(52A)-C(53A)-C(54A)	119.4(18)
C(40)-C(39)-N(4)	120.85(16)	C(53A)-C(54A)-C(55A)	120.3(18)
C(44)-C(39)-N(4)	117.63(15)	C(56A)-C(55A)-C(54A)	120.0(18)
C(41)-C(40)-C(39)	117.63(17)	C(55A)-C(56A)-C(57A)	119.9(18)
C(41)-C(40)-C(48)	118.50(17)	C(56A)-C(57A)-C(52A)	119.7(18)
C(39)-C(40)-C(48)	123.83(16)	C(60)-C(59)-C(64)	118.6(3)
C(42)-C(41)-C(40)	121.95(18)	C(60)-C(59)-C(58)	120.4(4)
C(41)-C(42)-C(43)	119.80(17)	C(64)-C(59)-C(58)	121.0(3)
C(42)-C(43)-C(44)	121.15(18)	C(61)-C(60)-C(59)	120.8(3)
C(43)-C(44)-C(39)	117.98(17)	C(60)-C(61)-C(62)	120.4(4)
C(43)-C(44)-C(45)	120.26(17)	C(61)-C(62)-C(63)	119.3(4)
C(39)-C(44)-C(45)	121.62(15)	C(64)-C(63)-C(62)	120.0(4)
C(44)-C(45)-C(46)	113.53(15)	C(63)-C(64)-C(59)	120.9(3)
C(44)-C(45)-C(47)	110.65(16)	C(64A)-C(59A)-C(60A)	119.3(10)
C(46)-C(45)-C(47)	108.70(16)	C(64A)-C(59A)-C(58A)	117.0(11)
C(40)-C(48)-C(50)	112.19(16)	C(60A)-C(59A)-C(58A)	123.8(12)
C(40)-C(48)-C(49)	109.79(17)	C(61A)-C(60A)-C(59A)	119.4(11)
C(50)-C(48)-C(49)	110.07(17)	C(60A)-C(61A)-C(62A)	121.2(12)
C(53)-C(52)-C(57)	118.0(2)	C(63A)-C(62A)-C(61A)	120.0(13)
C(53)-C(52)-C(51)	121.5(2)	C(62A)-C(63A)-C(64A)	118.4(14)
C(57)-C(52)-C(51)	120.5(2)	C(59A)-C(64A)-C(63A)	121.6(12)

Crystal Structure of 2

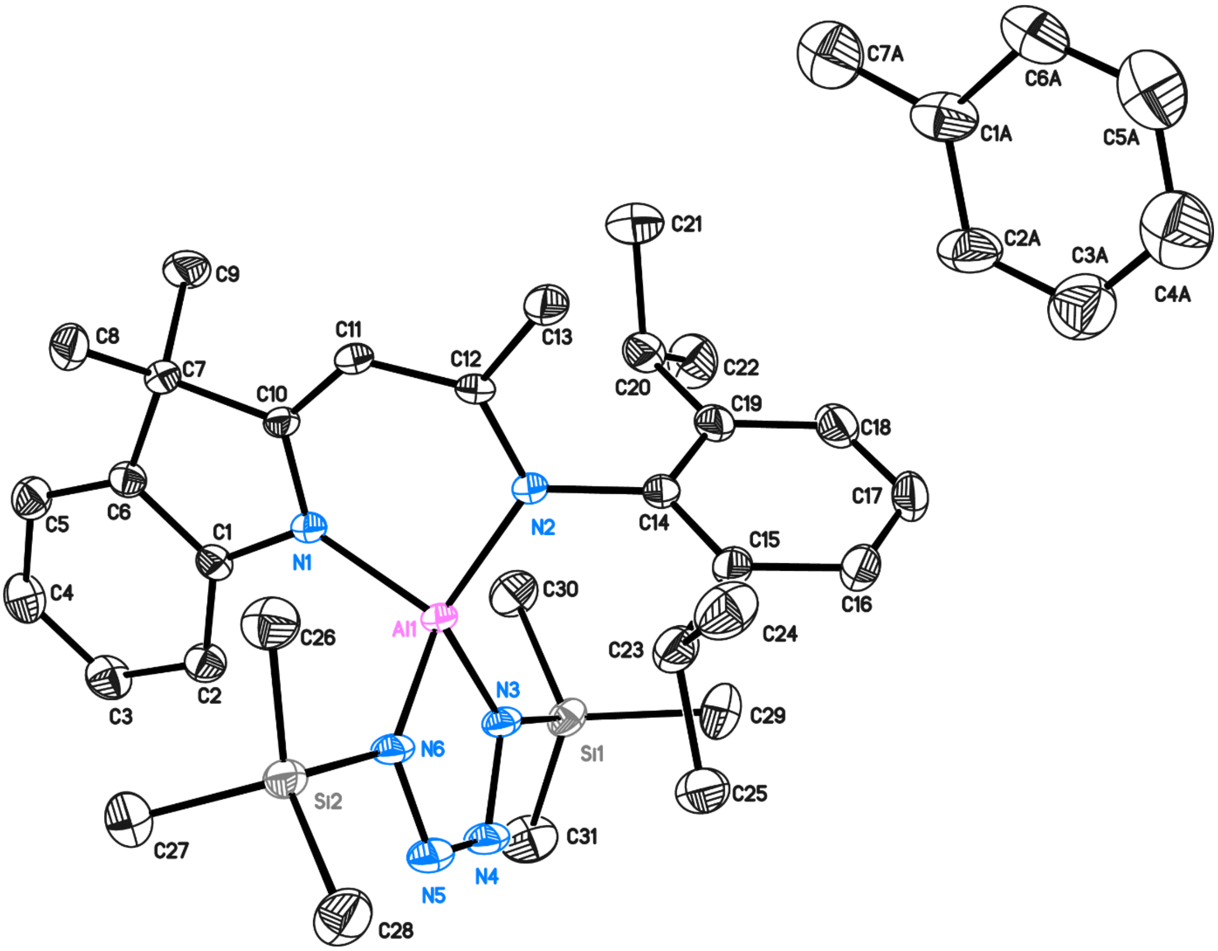

Figure S31: Asymmetric unit of **2** with thermal ellipsoids shown at 50% probability level. Hydrogen atoms are omitted for clarity. One toluene molecule (C1A) is disordered about an two-fold axis and was refined with distance restraints and restraints for anisotropic displacement parameters.

Table S4: Bond lengths [Å] and angles [°] for **2**.

Si(1)-N(3)	1.7417(13)	N(1)-C(10)	1.3448(18)
Si(1)-C(29)	1.858(2)	N(1)-C(1)	1.4255(19)
Si(1)-C(31)	1.8600(18)	N(2)-C(12)	1.3573(18)
Si(1)-C(30)	1.8631(18)	N(2)-C(14)	1.4529(19)
Si(2)-N(6)	1.7414(14)	N(3)-N(4)	1.4128(17)
Si(2)-C(28)	1.8589(18)	N(4)-N(5)	1.2671(19)
Si(2)-C(26)	1.8618(18)	N(5)-N(6)	1.4010(17)
Si(2)-C(27)	1.8672(19)	C(1)-C(2)	1.386(2)
Al(1)-N(3)	1.8260(13)	C(1)-C(6)	1.391(2)
Al(1)-N(6)	1.8312(13)	C(2)-C(3)	1.390(2)
Al(1)-N(1)	1.8708(13)	C(3)-C(4)	1.387(2)
Al(1)-N(2)	1.8812(13)	C(4)-C(5)	1.395(2)

C(5)-C(6)	1.386(2)
C(6)-C(7)	1.512(2)
C(7)-C(10)	1.527(2)
C(7)-C(8)	1.536(2)
C(7)-C(9)	1.541(2)
C(10)-C(11)	1.389(2)
C(11)-C(12)	1.392(2)
C(12)-C(13)	1.508(2)
C(14)-C(15)	1.404(2)
C(14)-C(19)	1.412(2)
C(15)-C(16)	1.396(2)
C(15)-C(23)	1.517(2)
C(16)-C(17)	1.374(3)
C(17)-C(18)	1.385(3)
C(18)-C(19)	1.397(2)
C(19)-C(20)	1.526(2)
C(20)-C(22)	1.533(2)
C(20)-C(21)	1.540(2)
C(23)-C(25)	1.534(2)
C(23)-C(24)	1.536(2)
C(1A)-C(2A)	1.386(7)
C(1A)-C(6A)	1.407(8)
C(1A)-C(7A)	1.494(14)
C(2A)-C(3A)	1.387(10)
C(3A)-C(4A)	1.391(9)
C(4A)-C(5A)	1.376(9)
C(5A)-C(6A)	1.379(9)
N(3)-Si(1)-C(29)	111.15(8)
N(3)-Si(1)-C(31)	110.14(8)
C(29)-Si(1)-C(31)	109.37(9)
N(3)-Si(1)-C(30)	105.59(7)
C(29)-Si(1)-C(30)	110.81(9)
C(31)-Si(1)-C(30)	109.72(9)
N(6)-Si(2)-C(28)	110.58(8)
N(6)-Si(2)-C(26)	106.34(7)
C(28)-Si(2)-C(26)	110.24(9)
N(6)-Si(2)-C(27)	110.07(8)
C(28)-Si(2)-C(27)	110.31(9)
C(26)-Si(2)-C(27)	109.23(9)
N(3)-Al(1)-N(6)	87.04(6)

N(3)-Al(1)-N(1)	121.20(6)
N(6)-Al(1)-N(1)	110.76(6)
N(3)-Al(1)-N(2)	121.87(6)
N(6)-Al(1)-N(2)	121.80(6)
N(1)-Al(1)-N(2)	95.99(6)
C(10)-N(1)-C(1)	108.63(12)
C(10)-N(1)-Al(1)	121.55(10)
C(1)-N(1)-Al(1)	128.45(10)
C(12)-N(2)-C(14)	118.00(12)
C(12)-N(2)-Al(1)	120.89(10)
C(14)-N(2)-Al(1)	121.02(9)
N(4)-N(3)-Si(1)	111.85(9)
N(4)-N(3)-Al(1)	109.82(9)
Si(1)-N(3)-Al(1)	138.32(8)
N(5)-N(4)-N(3)	116.59(12)
N(4)-N(5)-N(6)	116.21(12)
N(5)-N(6)-Si(2)	112.75(10)
N(5)-N(6)-Al(1)	110.32(10)
Si(2)-N(6)-Al(1)	135.59(7)
C(2)-C(1)-C(6)	122.56(14)
C(2)-C(1)-N(1)	127.10(14)
C(6)-C(1)-N(1)	110.29(13)
C(1)-C(2)-C(3)	117.20(15)
C(4)-C(3)-C(2)	121.16(15)
C(3)-C(4)-C(5)	120.82(15)
C(6)-C(5)-C(4)	118.69(15)
C(5)-C(6)-C(1)	119.56(14)
C(5)-C(6)-C(7)	131.57(14)
C(1)-C(6)-C(7)	108.87(13)
C(6)-C(7)-C(10)	100.59(12)
C(6)-C(7)-C(8)	113.13(13)
C(10)-C(7)-C(8)	111.72(13)
C(6)-C(7)-C(9)	112.10(13)
C(10)-C(7)-C(9)	108.56(12)
C(8)-C(7)-C(9)	110.33(13)
N(1)-C(10)-C(11)	124.78(13)
N(1)-C(10)-C(7)	111.50(12)
C(11)-C(10)-C(7)	123.72(13)
C(10)-C(11)-C(12)	125.43(13)
N(2)-C(12)-C(11)	122.74(13)
N(2)-C(12)-C(13)	119.79(13)

C(11)-C(12)-C(13)	117.42(13)	C(19)-C(20)-C(21)	112.12(15)
C(15)-C(14)-C(19)	121.37(14)	C(22)-C(20)-C(21)	109.17(15)
C(15)-C(14)-N(2)	120.76(14)	C(15)-C(23)-C(25)	111.20(14)
C(19)-C(14)-N(2)	117.84(14)	C(15)-C(23)-C(24)	111.13(14)
C(16)-C(15)-C(14)	118.00(16)	C(25)-C(23)-C(24)	110.87(14)
C(16)-C(15)-C(23)	119.17(15)	C(2A)-C(1A)-C(6A)	118.7(9)
C(14)-C(15)-C(23)	122.83(14)	C(2A)-C(1A)-C(7A)	122.3(8)
C(17)-C(16)-C(15)	121.62(17)	C(6A)-C(1A)-C(7A)	118.9(7)
C(16)-C(17)-C(18)	119.86(16)	C(3A)-C(2A)-C(1A)	119.5(9)
C(17)-C(18)-C(19)	121.28(17)	C(2A)-C(3A)-C(4A)	121.3(4)
C(18)-C(19)-C(14)	117.82(16)	C(5A)-C(4A)-C(3A)	119.3(10)
C(18)-C(19)-C(20)	119.49(15)	C(4A)-C(5A)-C(6A)	120.0(10)
C(14)-C(19)-C(20)	122.69(14)	C(5A)-C(6A)-C(1A)	121.1(4)
C(19)-C(20)-C(22)	112.15(14)		

Crystal Structure of 3

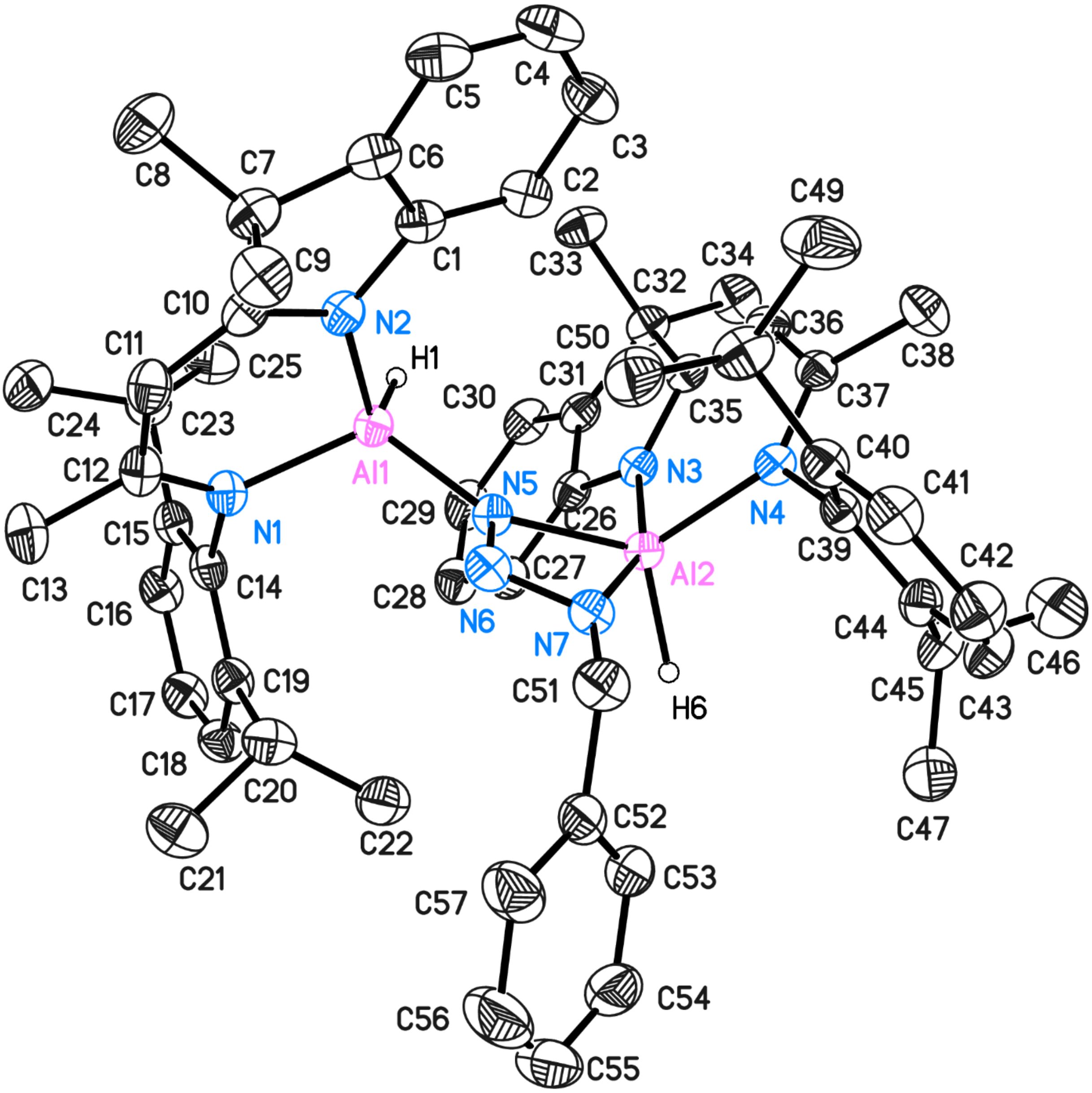

Figure S32: Asymmetric unit of **3** with thermal ellipsoids shown at 50% probability level. All ligand based hydrogen atoms are omitted for clarity. The hydrogen atoms bond to the aluminium atoms were refined freely. One extremely disordered hexane molecule was treated with SQUEEZE, which found an electron count of 51 e^- in a void of 264 $Å^3$.[7]

Table S5: Bond lengths [Å] and angles [°] for **3**.

Al(1)-H(1)	1.499(18)	Al(2)-N(4)	1.9405(14)
Al(1)-N(5)	1.8361(14)	Al(2)-N(3)	1.9492(14)
Al(1)-N(2)	1.8877(14)	Al(2)-N(7)	2.1633(15)
Al(1)-N(1)	1.9061(15)	N(1)-C(12)	1.334(2)
Al(2)-H(6)	1.581(18)	N(1)-C(14)	1.458(2)
Al(2)-N(5)	1.9074(14)	N(2)-C(10)	1.346(2)

N(2)-C(1)	1.422(2)	C(42)-C(43)	1.382(3)
N(3)-C(35)	1.333(2)	C(43)-C(44)	1.391(2)
N(3)-C(26)	1.422(2)	C(44)-C(45)	1.522(2)
N(4)-C(37)	1.344(2)	C(45)-C(47)	1.523(3)
N(4)-C(39)	1.455(2)	C(45)-C(46)	1.525(3)
N(5)-N(6)	1.3436(19)	C(48)-C(50)	1.516(3)
N(6)-N(7)	1.288(2)	C(48)-C(49)	1.536(3)
N(7)-C(51)	1.465(2)	C(51)-C(52)	1.511(3)
C(1)-C(2)	1.382(3)	C(52)-C(53)	1.382(3)
C(1)-C(6)	1.391(2)	C(52)-C(57)	1.391(3)
C(2)-C(3)	1.396(2)	C(53)-C(54)	1.381(3)
C(14)-C(19)	1.404(2)	C(54)-C(55)	1.374(4)
C(14)-C(15)	1.409(2)	C(55)-C(56)	1.378(4)
C(15)-C(16)	1.392(2)	C(56)-C(57)	1.386(3)
C(15)-C(23)	1.523(2)	C(3)-C(4)	1.384(3)
C(16)-C(17)	1.380(3)	C(4)-C(5)	1.389(3)
C(17)-C(18)	1.380(2)	C(5)-C(6)	1.383(3)
C(18)-C(19)	1.393(2)	C(6)-C(7)	1.504(3)
C(19)-C(20)	1.522(2)	C(7)-C(8)	1.530(3)
C(20)-C(22)	1.528(3)	C(7)-C(10)	1.534(2)
C(20)-C(21)	1.537(3)	C(7)-C(9)	1.540(2)
C(23)-C(25)	1.526(3)	C(10)-C(11)	1.381(2)
C(23)-C(24)	1.532(3)	C(11)-C(12)	1.406(2)
C(26)-C(27)	1.382(2)	C(12)-C(13)	1.506(2)
C(26)-C(31)	1.394(2)		
C(27)-C(28)	1.389(2)	H(1)-Al(1)-N(5)	111.9(7)
C(28)-C(29)	1.387(3)	H(1)-Al(1)-N(2)	113.6(7)
C(29)-C(30)	1.386(3)	N(5)-Al(1)-N(2)	107.40(6)
C(30)-C(31)	1.383(2)	H(1)-Al(1)-N(1)	111.2(7)
C(31)-C(32)	1.504(2)	N(5)-Al(1)-N(1)	116.18(6)
C(32)-C(34)	1.525(2)	N(2)-Al(1)-N(1)	95.52(6)
C(32)-C(35)	1.531(2)	H(6)-Al(2)-N(5)	118.4(7)
C(32)-C(33)	1.539(2)	H(6)-Al(2)-N(4)	116.1(7)
C(35)-C(36)	1.388(2)	N(5)-Al(2)-N(4)	121.37(6)
C(36)-C(37)	1.394(2)	H(6)-Al(2)-N(3)	102.3(6)
C(37)-C(38)	1.513(2)	N(5)-Al(2)-N(3)	96.00(6)
C(39)-C(44)	1.404(2)	N(4)-Al(2)-N(3)	91.98(6)
C(39)-C(40)	1.405(2)	H(6)-Al(2)-N(7)	93.3(6)
C(40)-C(41)	1.396(2)	N(5)-Al(2)-N(7)	62.78(6)
C(40)-C(48)	1.521(2)	N(4)-Al(2)-N(7)	94.88(6)
C(41)-C(42)	1.377(3)	N(3)-Al(2)-N(7)	158.12(6)

C(12)-N(1)-C(14)	119.97(14)
C(12)-N(1)-Al(1)	125.62(12)
C(14)-N(1)-Al(1)	114.32(10)
C(10)-N(2)-C(1)	108.57(14)
C(10)-N(2)-Al(1)	124.06(12)
C(1)-N(2)-Al(1)	126.92(11)
C(35)-N(3)-C(26)	108.18(13)
C(35)-N(3)-Al(2)	125.62(11)
C(26)-N(3)-Al(2)	125.80(11)
C(37)-N(4)-C(39)	117.19(13)
C(37)-N(4)-Al(2)	126.57(11)
C(39)-N(4)-Al(2)	116.19(10)
N(6)-N(5)-Al(1)	118.79(10)
N(6)-N(5)-Al(2)	99.49(10)
Al(1)-N(5)-Al(2)	140.97(8)
N(7)-N(6)-N(5)	108.17(13)
N(6)-N(7)-C(51)	114.20(14)
N(6)-N(7)-Al(2)	89.49(9)
C(51)-N(7)-Al(2)	155.70(12)
C(2)-C(1)-C(6)	122.12(16)
C(2)-C(1)-N(2)	127.23(15)
C(6)-C(1)-N(2)	110.65(15)
C(1)-C(2)-C(3)	117.25(17)
C(19)-C(14)-C(15)	120.95(15)
C(19)-C(14)-N(1)	120.11(14)
C(15)-C(14)-N(1)	118.85(15)
C(16)-C(15)-C(14)	118.37(16)
C(16)-C(15)-C(23)	118.86(15)
C(14)-C(15)-C(23)	122.77(15)
C(17)-C(16)-C(15)	121.28(16)
C(16)-C(17)-C(18)	119.63(16)
C(17)-C(18)-C(19)	121.60(17)
C(18)-C(19)-C(14)	118.12(15)
C(18)-C(19)-C(20)	118.42(16)
C(14)-C(19)-C(20)	123.42(15)
C(19)-C(20)-C(22)	111.81(16)
C(19)-C(20)-C(21)	110.65(15)
C(22)-C(20)-C(21)	109.60(16)
C(15)-C(23)-C(25)	111.20(15)
C(15)-C(23)-C(24)	111.50(15)
C(25)-C(23)-C(24)	109.69(16)
C(27)-C(26)-C(31)	121.84(15)
C(27)-C(26)-N(3)	127.58(15)
C(31)-C(26)-N(3)	110.58(14)
C(26)-C(27)-C(28)	117.36(16)
C(29)-C(28)-C(27)	121.26(17)
C(30)-C(29)-C(28)	120.86(17)
C(31)-C(30)-C(29)	118.39(17)
C(30)-C(31)-C(26)	120.26(16)
C(30)-C(31)-C(32)	130.97(16)
C(26)-C(31)-C(32)	108.65(14)
C(31)-C(32)-C(34)	114.24(14)
C(31)-C(32)-C(35)	100.19(13)
C(34)-C(32)-C(35)	112.93(14)
C(31)-C(32)-C(33)	109.87(14)
C(34)-C(32)-C(33)	110.13(15)
C(35)-C(32)-C(33)	109.03(13)
N(3)-C(35)-C(36)	124.43(14)
N(3)-C(35)-C(32)	112.01(14)
C(36)-C(35)-C(32)	123.54(14)
C(35)-C(36)-C(37)	125.46(15)
N(4)-C(37)-C(36)	123.64(15)
N(4)-C(37)-C(38)	120.70(15)
C(36)-C(37)-C(38)	115.64(15)
C(44)-C(39)-C(40)	121.04(15)
C(44)-C(39)-N(4)	118.58(14)
C(40)-C(39)-N(4)	120.38(14)
C(41)-C(40)-C(39)	118.12(16)
C(41)-C(40)-C(48)	118.64(15)
C(39)-C(40)-C(48)	123.12(15)
C(42)-C(41)-C(40)	121.54(16)
C(41)-C(42)-C(43)	119.46(16)
C(42)-C(43)-C(44)	121.54(17)
C(43)-C(44)-C(39)	118.29(16)
C(43)-C(44)-C(45)	118.86(15)
C(39)-C(44)-C(45)	122.78(15)
C(44)-C(45)-C(47)	112.52(15)
C(44)-C(45)-C(46)	110.56(15)
C(47)-C(45)-C(46)	109.98(16)
C(50)-C(48)-C(40)	114.28(16)
C(50)-C(48)-C(49)	108.54(16)
C(40)-C(48)-C(49)	110.05(17)

N(7)-C(51)-C(52)	113.47(15)	C(1)-C(6)-C(7)	108.70(15)
C(53)-C(52)-C(57)	119.13(19)	C(6)-C(7)-C(8)	113.75(15)
C(53)-C(52)-C(51)	121.57(17)	C(6)-C(7)-C(10)	100.82(13)
C(57)-C(52)-C(51)	119.30(19)	C(8)-C(7)-C(10)	112.33(15)
C(54)-C(53)-C(52)	121.0(2)	C(6)-C(7)-C(9)	110.43(15)
C(55)-C(54)-C(53)	119.6(2)	C(8)-C(7)-C(9)	109.95(15)
C(54)-C(55)-C(56)	120.1(2)	C(10)-C(7)-C(9)	109.21(14)
C(55)-C(56)-C(57)	120.5(2)	N(2)-C(10)-C(11)	124.65(15)
C(56)-C(57)-C(52)	119.6(2)	N(2)-C(10)-C(7)	111.13(15)
C(4)-C(3)-C(2)	121.27(18)	C(11)-C(10)-C(7)	124.22(15)
C(3)-C(4)-C(5)	120.54(17)	C(10)-C(11)-C(12)	126.34(16)
C(6)-C(5)-C(4)	118.95(17)	N(1)-C(12)-C(11)	122.67(16)
C(5)-C(6)-C(1)	119.87(17)	N(1)-C(12)-C(13)	120.02(15)
C(5)-C(6)-C(7)	131.39(16)	C(11)-C(12)-C(13)	117.32(15)

Crystal Structure of 4

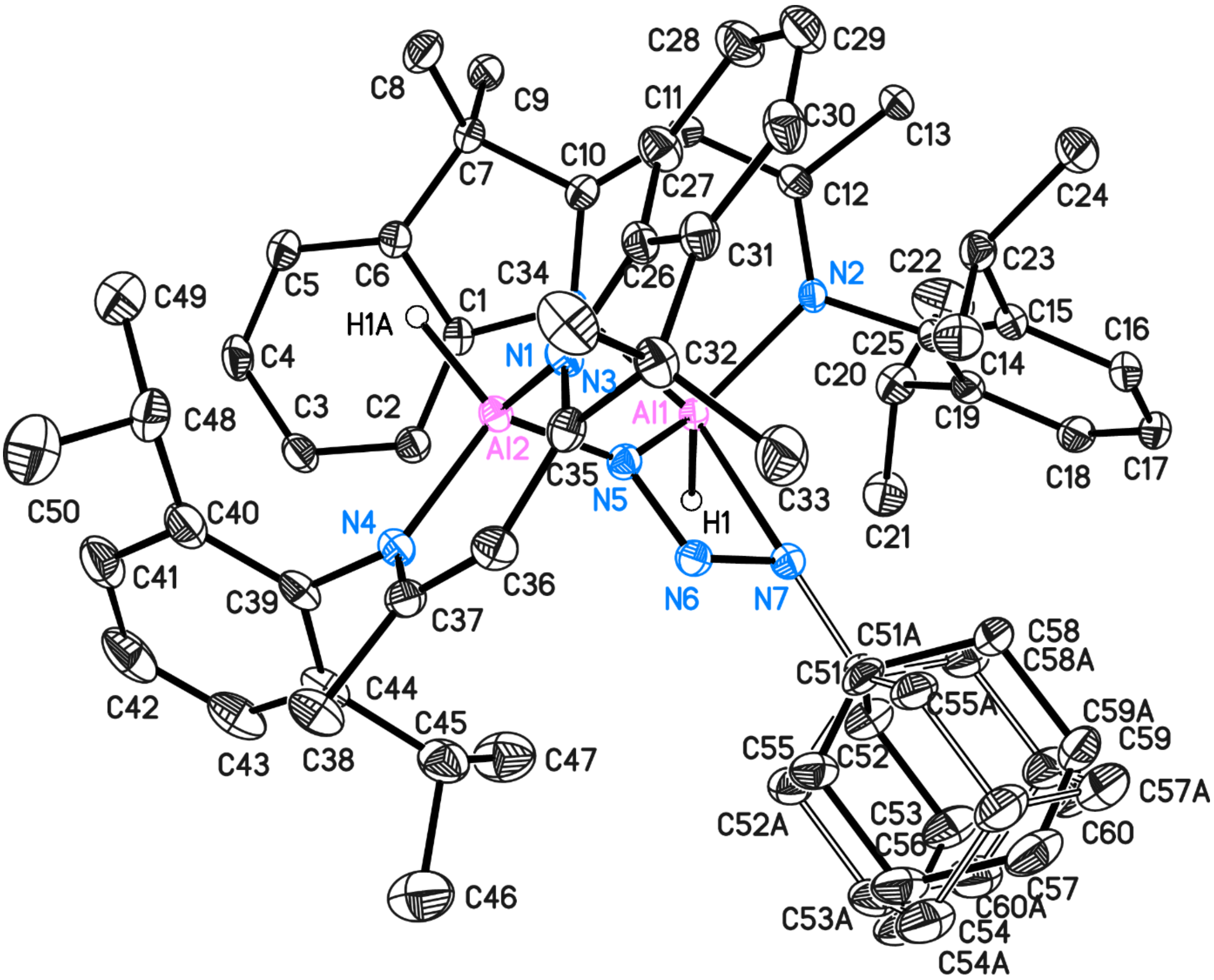

Figure S33: Asymmetric unit of **4** with thermal ellipsoids shown at 50% probability level. All ligand based hydrogen atoms are omitted for clarity. The hydrogen atoms bond to aluminium atoms were refined freely The Al-H distances were restrained to be similar. The hydrogen atoms bond to C11 and C36 were refined freely. Both C-H distances were restrained to be similar. One 1-Adamantyl moiety is disordered over 2 positions (C51, C51A) and is refined with distance restraints and restraints for the anisotropic displacement parameters. The occupancies of the minor positions refined to 0.202(2). A disordered mixture of toluene and hexane was treated with SQUEEZE, which found an electron count of 53 e^- in a void of 291 $Å^3$.[7]

Table S6: Bond lengths [Å] and angles [°] for **4**.

Al(1)-H(1)	1.527(14)	Al(2)-N(5)	1.8321(11)
Al(1)-N(5)	1.8765(11)	Al(2)-N(3)	1.8829(11)
Al(1)-N(2)	1.9453(11)	Al(2)-N(4)	1.9083(11)
Al(1)-N(1)	1.9460(11)	N(1)-C(10)	1.3295(16)
Al(1)-N(7)	2.3347(12)	N(1)-C(1)	1.4193(16)
Al(2)-H(1A)	1.515(14)	N(2)-C(12)	1.3460(16)

N(2)-C(14)	1.4501(15)	C(31)-C(32)	1.5133(19)
N(3)-C(35)	1.3430(17)	C(32)-C(35)	1.5328(18)
N(3)-C(26)	1.4121(16)	C(32)-C(33)	1.534(2)
N(4)-C(37)	1.3328(17)	C(32)-C(34)	1.5365(19)
N(4)-C(39)	1.4502(16)	C(35)-C(36)	1.3859(19)
N(5)-N(6)	1.3565(15)	C(36)-H(36)	0.955(14)
N(6)-N(7)	1.2739(15)	C(36)-C(37)	1.4071(19)
N(7)-C(51)	1.471(5)	C(37)-C(38)	1.5043(18)
N(7)-C(51A)	1.530(18)	C(39)-C(40)	1.405(2)
C(1)-C(2)	1.3838(18)	C(39)-C(44)	1.410(2)
C(1)-C(6)	1.3946(18)	C(40)-C(41)	1.396(2)
C(2)-C(3)	1.3956(18)	C(40)-C(48)	1.521(2)
C(3)-C(4)	1.385(2)	C(41)-C(42)	1.379(3)
C(4)-C(5)	1.394(2)	C(42)-C(43)	1.380(3)
C(5)-C(6)	1.3840(18)	C(43)-C(44)	1.398(2)
C(6)-C(7)	1.5105(18)	C(44)-C(45)	1.528(2)
C(7)-C(10)	1.5289(17)	C(45)-C(47)	1.523(2)
C(7)-C(9)	1.5361(19)	C(45)-C(46)	1.536(2)
C(7)-C(8)	1.5392(19)	C(48)-C(49)	1.532(2)
C(10)-C(11)	1.3909(18)	C(48)-C(50)	1.537(2)
C(11)-H(11)	0.939(14)	C(51)-C(58)	1.533(4)
C(11)-C(12)	1.3913(18)	C(51)-C(52)	1.535(4)
C(12)-C(13)	1.5099(17)	C(51)-C(55)	1.536(3)
C(14)-C(19)	1.4061(18)	C(52)-C(53)	1.539(3)
C(14)-C(15)	1.4094(18)	C(53)-C(60)	1.527(4)
C(15)-C(16)	1.3972(18)	C(53)-C(54)	1.530(3)
C(15)-C(23)	1.5237(18)	C(54)-C(56)	1.523(3)
C(16)-C(17)	1.389(2)	C(55)-C(56)	1.542(3)
C(17)-C(18)	1.382(2)	C(56)-C(57)	1.529(3)
C(18)-C(19)	1.3964(18)	C(57)-C(59)	1.527(3)
C(19)-C(20)	1.5238(19)	C(58)-C(59)	1.538(3)
C(20)-C(21)	1.526(2)	C(59)-C(60)	1.518(4)
C(20)-C(22)	1.536(2)	C(51A)-C(55A)	1.513(13)
C(23)-C(25)	1.5294(19)	C(51A)-C(52A)	1.517(13)
C(23)-C(24)	1.5368(18)	C(51A)-C(58A)	1.533(13)
C(26)-C(27)	1.3866(19)	C(52A)-C(53A)	1.540(9)
C(26)-C(31)	1.3927(18)	C(53A)-C(54A)	1.519(11)
C(27)-C(28)	1.3954(19)	C(53A)-C(60A)	1.521(10)
C(28)-C(29)	1.391(2)	C(54A)-C(56A)	1.511(10)
C(29)-C(30)	1.393(2)	C(55A)-C(56A)	1.545(9)
C(30)-C(31)	1.3819(19)	C(56A)-C(57A)	1.505(11)

C(57A)-C(59A)	1.522(12)
C(58A)-C(59A)	1.542(10)
C(59A)-C(60A)	1.545(11)
H(1)-Al(1)-N(5)	117.6(6)
H(1)-Al(1)-N(2)	114.9(6)
N(5)-Al(1)-N(2)	123.22(5)
H(1)-Al(1)-N(1)	103.3(6)
N(5)-Al(1)-N(1)	95.89(5)
N(2)-Al(1)-N(1)	91.82(5)
H(1)-Al(1)-N(7)	88.0(6)
N(5)-Al(1)-N(7)	60.16(4)
N(2)-Al(1)-N(7)	102.66(4)
N(1)-Al(1)-N(7)	155.99(4)
H(1A)-Al(2)-N(5)	113.0(6)
H(1A)-Al(2)-N(3)	112.0(6)
N(5)-Al(2)-N(3)	107.31(5)
H(1A)-Al(2)-N(4)	112.3(6)
N(5)-Al(2)-N(4)	115.81(5)
N(3)-Al(2)-N(4)	94.90(5)
C(10)-N(1)-C(1)	108.17(10)
C(10)-N(1)-Al(1)	126.35(9)
C(1)-N(1)-Al(1)	125.39(8)
C(12)-N(2)-C(14)	117.03(10)
C(12)-N(2)-Al(1)	126.44(9)
C(14)-N(2)-Al(1)	116.40(8)
C(35)-N(3)-C(26)	108.89(11)
C(35)-N(3)-Al(2)	123.82(9)
C(26)-N(3)-Al(2)	126.64(9)
C(37)-N(4)-C(39)	120.00(11)
C(37)-N(4)-Al(2)	125.33(9)
C(39)-N(4)-Al(2)	114.51(8)
N(6)-N(5)-Al(2)	117.44(8)
N(6)-N(5)-Al(1)	103.90(8)
Al(2)-N(5)-Al(1)	138.62(6)
N(7)-N(6)-N(5)	109.42(10)
N(6)-N(7)-C(51)	115.50(15)
N(6)-N(7)-C(51A)	113.2(5)
N(6)-N(7)-Al(1)	85.28(7)
C(51)-N(7)-Al(1)	153.27(14)
C(51A)-N(7)-Al(1)	155.0(5)
C(2)-C(1)-C(6)	121.96(12)
C(2)-C(1)-N(1)	127.17(11)
C(6)-C(1)-N(1)	110.86(11)
C(1)-C(2)-C(3)	117.42(12)
C(4)-C(3)-C(2)	121.03(12)
C(3)-C(4)-C(5)	121.04(13)
C(6)-C(5)-C(4)	118.36(13)
C(5)-C(6)-C(1)	120.18(12)
C(5)-C(6)-C(7)	131.46(12)
C(1)-C(6)-C(7)	108.34(11)
C(6)-C(7)-C(10)	100.15(10)
C(6)-C(7)-C(9)	112.89(11)
C(10)-C(7)-C(9)	110.20(10)
C(6)-C(7)-C(8)	111.57(11)
C(10)-C(7)-C(8)	110.57(11)
C(9)-C(7)-C(8)	110.99(11)
N(1)-C(10)-C(11)	124.48(11)
N(1)-C(10)-C(7)	112.42(11)
C(11)-C(10)-C(7)	122.98(11)
H(11)-C(11)-C(10)	117.2(10)
H(11)-C(11)-C(12)	117.1(10)
C(10)-C(11)-C(12)	125.03(12)
N(2)-C(12)-C(11)	123.94(11)
N(2)-C(12)-C(13)	119.70(11)
C(11)-C(12)-C(13)	116.31(11)
C(19)-C(14)-C(15)	121.51(11)
C(19)-C(14)-N(2)	118.59(11)
C(15)-C(14)-N(2)	119.89(11)
C(16)-C(15)-C(14)	117.80(12)
C(16)-C(15)-C(23)	119.58(12)
C(14)-C(15)-C(23)	122.62(11)
C(17)-C(16)-C(15)	121.33(13)
C(18)-C(17)-C(16)	119.71(12)
C(17)-C(18)-C(19)	121.37(13)
C(18)-C(19)-C(14)	118.03(12)
C(18)-C(19)-C(20)	119.78(12)
C(14)-C(19)-C(20)	122.04(11)
C(19)-C(20)-C(21)	113.25(12)
C(19)-C(20)-C(22)	110.28(12)
C(21)-C(20)-C(22)	109.51(12)
C(15)-C(23)-C(25)	112.23(11)

C(15)-C(23)-C(24)	111.66(11)	C(44)-C(45)-C(46)	111.27(13)
C(25)-C(23)-C(24)	109.63(11)	C(40)-C(48)-C(49)	111.79(14)
C(27)-C(26)-C(31)	122.10(12)	C(40)-C(48)-C(50)	110.73(13)
C(27)-C(26)-N(3)	126.98(12)	C(49)-C(48)-C(50)	110.43(14)
C(31)-C(26)-N(3)	110.91(11)	N(7)-C(51)-C(58)	109.9(2)
C(26)-C(27)-C(28)	117.19(13)	N(7)-C(51)-C(52)	106.5(2)
C(29)-C(28)-C(27)	121.15(13)	C(58)-C(51)-C(52)	108.8(3)
C(28)-C(29)-C(30)	120.77(13)	N(7)-C(51)-C(55)	114.2(3)
C(31)-C(30)-C(29)	118.54(13)	C(58)-C(51)-C(55)	108.2(2)
C(30)-C(31)-C(26)	120.23(13)	C(52)-C(51)-C(55)	109.2(2)
C(30)-C(31)-C(32)	131.43(12)	C(51)-C(52)-C(53)	110.6(2)
C(26)-C(31)-C(32)	108.33(11)	C(60)-C(53)-C(54)	110.0(2)
C(31)-C(32)-C(35)	100.64(10)	C(60)-C(53)-C(52)	109.24(18)
C(31)-C(32)-C(33)	111.97(12)	C(54)-C(53)-C(52)	108.41(18)
C(35)-C(32)-C(33)	111.27(12)	C(56)-C(54)-C(53)	109.59(19)
C(31)-C(32)-C(34)	110.83(12)	C(51)-C(55)-C(56)	109.4(2)
C(35)-C(32)-C(34)	110.35(11)	C(54)-C(56)-C(57)	109.5(2)
C(33)-C(32)-C(34)	111.33(12)	C(54)-C(56)-C(55)	109.62(18)
N(3)-C(35)-C(36)	124.59(12)	C(57)-C(56)-C(55)	109.87(18)
N(3)-C(35)-C(32)	111.22(11)	C(59)-C(57)-C(56)	109.52(19)
C(36)-C(35)-C(32)	124.14(12)	C(51)-C(58)-C(59)	110.7(2)
H(36)-C(36)-C(35)	117.5(10)	C(60)-C(59)-C(57)	109.2(2)
H(36)-C(36)-C(37)	116.7(10)	C(60)-C(59)-C(58)	110.0(2)
C(35)-C(36)-C(37)	125.54(12)	C(57)-C(59)-C(58)	108.8(2)
N(4)-C(37)-C(36)	122.68(12)	C(59)-C(60)-C(53)	109.8(2)
N(4)-C(37)-C(38)	120.54(12)	C(55A)-C(51A)-C(52A)	110.8(10)
C(36)-C(37)-C(38)	116.73(12)	C(55A)-C(51A)-N(7)	110.0(9)
C(40)-C(39)-C(44)	121.69(13)	C(52A)-C(51A)-N(7)	111.3(9)
C(40)-C(39)-N(4)	118.33(12)	C(55A)-C(51A)-C(58A)	110.1(10)
C(44)-C(39)-N(4)	119.88(12)	C(52A)-C(51A)-C(58A)	110.0(10)
C(41)-C(40)-C(39)	118.05(14)	N(7)-C(51A)-C(58A)	104.4(9)
C(41)-C(40)-C(48)	118.94(14)	C(51A)-C(52A)-C(53A)	108.3(8)
C(39)-C(40)-C(48)	123.00(13)	C(54A)-C(53A)-C(60A)	109.8(8)
C(42)-C(41)-C(40)	121.14(15)	C(54A)-C(53A)-C(52A)	110.0(7)
C(41)-C(42)-C(43)	120.13(14)	C(60A)-C(53A)-C(52A)	108.9(7)
C(42)-C(43)-C(44)	121.49(15)	C(56A)-C(54A)-C(53A)	110.2(8)
C(43)-C(44)-C(39)	117.50(14)	C(51A)-C(55A)-C(56A)	109.2(8)
C(43)-C(44)-C(45)	119.28(13)	C(57A)-C(56A)-C(54A)	109.8(9)
C(39)-C(44)-C(45)	123.20(13)	C(57A)-C(56A)-C(55A)	108.4(7)
C(47)-C(45)-C(44)	113.02(14)	C(54A)-C(56A)-C(55A)	109.0(7)
C(47)-C(45)-C(46)	109.51(13)	C(56A)-C(57A)-C(59A)	111.2(9)

C(51A)-C(58A)-C(59A)	108.4(9)	C(58A)-C(59A)-C(60A)	108.3(8)
C(57A)-C(59A)-C(58A)	109.3(8)	C(53A)-C(60A)-C(59A)	109.7(8)
C(57A)-C(59A)-C(60A)	109.0(9)		

Crystal Structure of 5

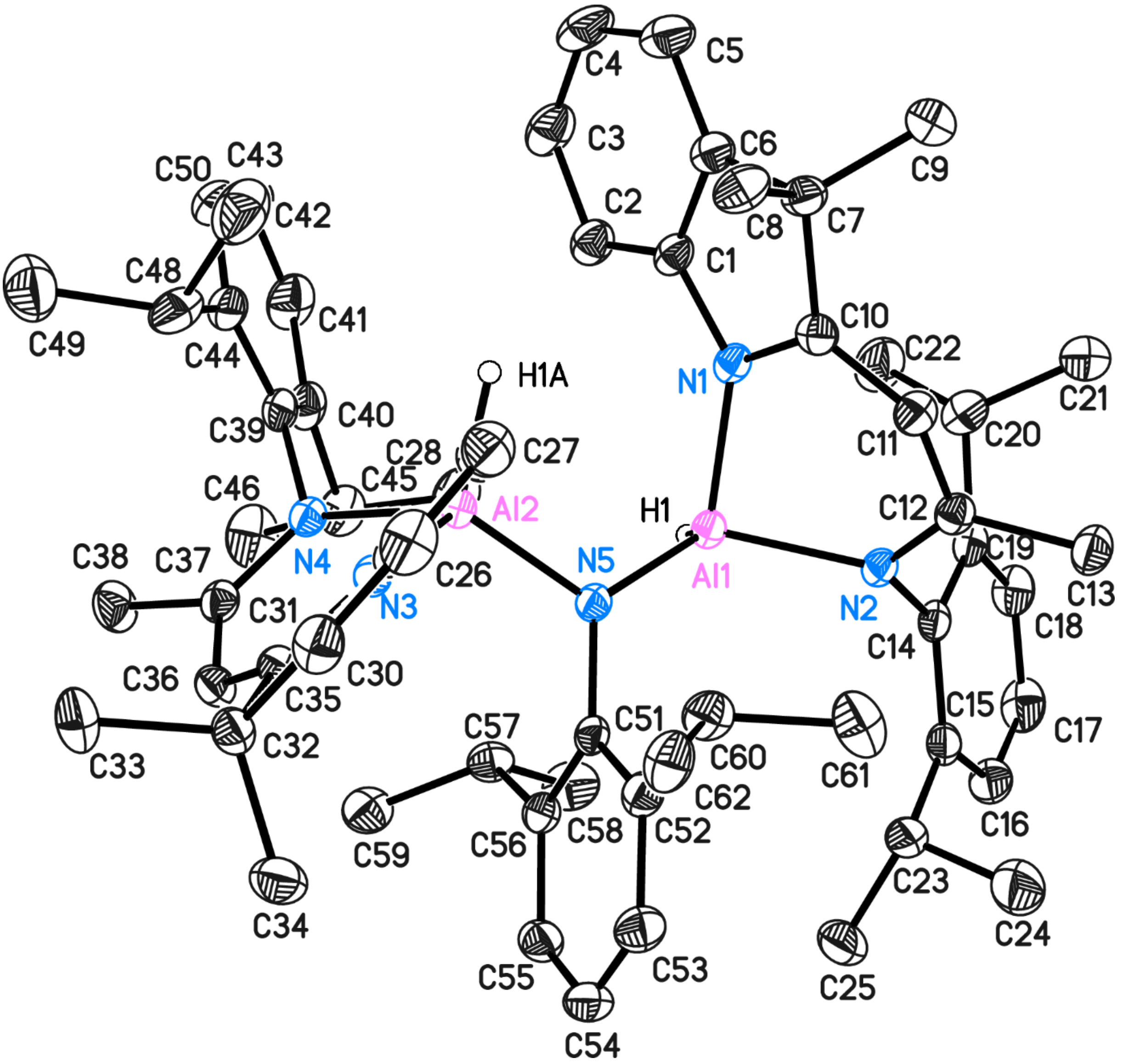

Figure S34: Asymmetric unit of **5** with thermal ellipsoids shown at 50% probability level. All ligand based hydrogen atoms are omitted for clarity. The hydrogen atoms bond to aluminium atoms were refined freely The Al-H distances were restrained to be similar. The hydrogen atoms bond to C11 and C36 were refined freely. Both C-H distances were restrained to be similar. Three (one and a half in the asymmetric unit) disordered benzene molecules were treated with SQUEEZE, which found an electron count of 133 e^{-} in a void of 566 Å^{3}.[7]

Table S7: Bond lengths [Å] and angles [°] for **5**.

Al(1)-H(1)	1.495(13)	Al(2)-N(3)	1.9035(11)
Al(1)-N(5)	1.8402(11)	Al(2)-N(4)	1.9543(11)
Al(1)-N(1)	1.9081(11)	N(1)-C(10)	1.3446(17)
Al(1)-N(2)	1.9318(11)	N(1)-C(1)	1.4219(16)
Al(2)-H(1A)	1.495(13)	N(2)-C(12)	1.3379(16)
Al(2)-N(5)	1.8295(11)	N(2)-C(14)	1.4600(16)

N(3)-C(35)	1.3462(16)	C(35)-C(36)	1.3882(18)
N(3)-C(26)	1.4189(16)	C(36)-H(36)	0.947(13)
N(4)-C(37)	1.3337(17)	C(36)-C(37)	1.4032(19)
N(4)-C(39)	1.4604(16)	C(37)-C(38)	1.5119(18)
N(5)-C(51)	1.4363(16)	C(39)-C(40)	1.405(2)
C(1)-C(2)	1.3854(19)	C(39)-C(44)	1.4078(19)
C(1)-C(6)	1.3944(19)	C(40)-C(41)	1.399(2)
C(2)-C(3)	1.393(2)	C(40)-C(45)	1.519(2)
C(3)-C(4)	1.384(2)	C(41)-C(42)	1.382(2)
C(4)-C(5)	1.397(2)	C(42)-C(43)	1.376(2)
C(5)-C(6)	1.3830(19)	C(43)-C(44)	1.393(2)
C(6)-C(7)	1.5095(19)	C(44)-C(48)	1.522(2)
C(7)-C(9)	1.5296(19)	C(45)-C(47)	1.531(2)
C(7)-C(10)	1.5317(17)	C(45)-C(46)	1.539(2)
C(7)-C(8)	1.5436(19)	C(48)-C(50)	1.520(2)
C(10)-C(11)	1.3892(18)	C(48)-C(49)	1.535(2)
C(11)-H(11)	0.950(13)	C(51)-C(56)	1.4202(18)
C(11)-C(12)	1.4045(18)	C(51)-C(52)	1.4247(18)
C(12)-C(13)	1.5096(18)	C(52)-C(53)	1.3952(19)
C(14)-C(15)	1.4054(18)	C(52)-C(60)	1.5215(19)
C(14)-C(19)	1.4079(19)	C(53)-C(54)	1.381(2)
C(15)-C(16)	1.401(2)	C(54)-C(55)	1.378(2)
C(15)-C(23)	1.519(2)	C(55)-C(56)	1.3981(18)
C(16)-C(17)	1.383(2)	C(56)-C(57)	1.5234(19)
C(17)-C(18)	1.375(2)	C(57)-C(58)	1.5282(19)
C(18)-C(19)	1.3949(19)	C(57)-C(59)	1.5327(19)
C(19)-C(20)	1.5247(19)	C(60)-C(62)	1.534(2)
C(20)-C(22)	1.522(2)	C(60)-C(61)	1.536(2)
C(20)-C(21)	1.530(2)		
C(23)-C(24)	1.529(2)	H(1)-Al(1)-N(5)	117.4(6)
C(23)-C(25)	1.532(2)	H(1)-Al(1)-N(1)	110.2(6)
C(26)-C(27)	1.3859(18)	N(5)-Al(1)-N(1)	109.09(5)
C(26)-C(31)	1.3953(18)	H(1)-Al(1)-N(2)	104.0(6)
C(27)-C(28)	1.3935(19)	N(5)-Al(1)-N(2)	120.46(5)
C(28)-C(29)	1.384(2)	N(1)-Al(1)-N(2)	92.79(5)
C(29)-C(30)	1.396(2)	H(1A)-Al(2)-N(5)	113.2(6)
C(30)-C(31)	1.3823(19)	H(1A)-Al(2)-N(3)	110.8(6)
C(31)-C(32)	1.5133(18)	N(5)-Al(2)-N(3)	112.22(5)
C(32)-C(35)	1.5332(18)	H(1A)-Al(2)-N(4)	106.7(6)
C(32)-C(34)	1.5390(19)	N(5)-Al(2)-N(4)	120.29(5)
C(32)-C(33)	1.5391(18)	N(3)-Al(2)-N(4)	91.75(5)

C(10)-N(1)-C(1)	107.97(10)	C(15)-C(14)-N(2)	121.80(12)
C(10)-N(1)-Al(1)	118.97(9)	C(19)-C(14)-N(2)	117.01(11)
C(1)-N(1)-Al(1)	132.90(9)	C(16)-C(15)-C(14)	117.55(13)
C(12)-N(2)-C(14)	117.71(10)	C(16)-C(15)-C(23)	119.32(12)
C(12)-N(2)-Al(1)	121.50(9)	C(14)-C(15)-C(23)	123.11(12)
C(14)-N(2)-Al(1)	117.87(8)	C(17)-C(16)-C(15)	121.82(13)
C(35)-N(3)-C(26)	108.17(10)	C(18)-C(17)-C(16)	119.72(13)
C(35)-N(3)-Al(2)	121.33(9)	C(17)-C(18)-C(19)	121.11(14)
C(26)-N(3)-Al(2)	130.43(8)	C(18)-C(19)-C(14)	118.65(12)
C(37)-N(4)-C(39)	118.69(10)	C(18)-C(19)-C(20)	118.38(12)
C(37)-N(4)-Al(2)	122.55(9)	C(14)-C(19)-C(20)	122.97(12)
C(39)-N(4)-Al(2)	116.76(8)	C(22)-C(20)-C(19)	111.92(13)
C(51)-N(5)-Al(2)	119.13(8)	C(22)-C(20)-C(21)	110.71(13)
C(51)-N(5)-Al(1)	121.68(8)	C(19)-C(20)-C(21)	111.01(12)
Al(2)-N(5)-Al(1)	118.27(6)	C(15)-C(23)-C(24)	111.57(12)
C(2)-C(1)-C(6)	121.53(12)	C(15)-C(23)-C(25)	112.22(13)
C(2)-C(1)-N(1)	127.84(12)	C(24)-C(23)-C(25)	109.01(13)
C(6)-C(1)-N(1)	110.63(11)	C(27)-C(26)-C(31)	121.66(12)
C(1)-C(2)-C(3)	117.67(14)	C(27)-C(26)-N(3)	127.47(12)
C(4)-C(3)-C(2)	121.29(14)	C(31)-C(26)-N(3)	110.86(11)
C(3)-C(4)-C(5)	120.56(14)	C(26)-C(27)-C(28)	117.46(13)
C(6)-C(5)-C(4)	118.55(14)	C(29)-C(28)-C(27)	121.46(13)
C(5)-C(6)-C(1)	120.38(13)	C(28)-C(29)-C(30)	120.47(13)
C(5)-C(6)-C(7)	130.87(13)	C(31)-C(30)-C(29)	118.65(13)
C(1)-C(6)-C(7)	108.71(11)	C(30)-C(31)-C(26)	120.27(12)
C(6)-C(7)-C(9)	113.43(12)	C(30)-C(31)-C(32)	131.24(12)
C(6)-C(7)-C(10)	100.02(10)	C(26)-C(31)-C(32)	108.49(11)
C(9)-C(7)-C(10)	113.36(11)	C(31)-C(32)-C(35)	100.31(10)
C(6)-C(7)-C(8)	110.29(11)	C(31)-C(32)-C(34)	111.85(11)
C(9)-C(7)-C(8)	110.38(12)	C(35)-C(32)-C(34)	110.17(11)
C(10)-C(7)-C(8)	108.91(11)	C(31)-C(32)-C(33)	111.56(11)
N(1)-C(10)-C(11)	124.64(12)	C(35)-C(32)-C(33)	111.85(11)
N(1)-C(10)-C(7)	111.69(11)	C(34)-C(32)-C(33)	110.71(11)
C(11)-C(10)-C(7)	123.36(12)	N(3)-C(35)-C(36)	124.35(12)
H(11)-C(11)-C(10)	116.7(9)	N(3)-C(35)-C(32)	111.54(11)
H(11)-C(11)-C(12)	117.4(9)	C(36)-C(35)-C(32)	123.75(11)
C(10)-C(11)-C(12)	123.27(12)	H(36)-C(36)-C(35)	117.9(9)
N(2)-C(12)-C(11)	122.36(12)	H(36)-C(36)-C(37)	116.9(9)
N(2)-C(12)-C(13)	120.86(11)	C(35)-C(36)-C(37)	123.57(12)
C(11)-C(12)-C(13)	116.65(11)	N(4)-C(37)-C(36)	122.66(12)
C(15)-C(14)-C(19)	121.14(12)	N(4)-C(37)-C(38)	120.40(12)

C(36)-C(37)-C(38)	116.78(12)	C(56)-C(51)-C(52)	117.76(12)
C(40)-C(39)-C(44)	120.77(12)	C(56)-C(51)-N(5)	121.72(11)
C(40)-C(39)-N(4)	122.46(12)	C(52)-C(51)-N(5)	120.52(11)
C(44)-C(39)-N(4)	116.71(12)	C(53)-C(52)-C(51)	119.54(12)
C(41)-C(40)-C(39)	118.07(14)	C(53)-C(52)-C(60)	118.21(12)
C(41)-C(40)-C(45)	118.89(13)	C(51)-C(52)-C(60)	122.22(12)
C(39)-C(40)-C(45)	122.88(12)	C(54)-C(53)-C(52)	122.24(13)
C(42)-C(41)-C(40)	121.38(15)	C(55)-C(54)-C(53)	118.66(13)
C(43)-C(42)-C(41)	119.76(14)	C(54)-C(55)-C(56)	121.67(13)
C(42)-C(43)-C(44)	121.30(14)	C(55)-C(56)-C(51)	120.13(12)
C(43)-C(44)-C(39)	118.47(14)	C(55)-C(56)-C(57)	117.83(12)
C(43)-C(44)-C(48)	118.90(13)	C(51)-C(56)-C(57)	122.04(11)
C(39)-C(44)-C(48)	122.56(12)	C(56)-C(57)-C(58)	111.81(12)
C(40)-C(45)-C(47)	113.18(13)	C(56)-C(57)-C(59)	111.89(11)
C(40)-C(45)-C(46)	110.43(12)	C(58)-C(57)-C(59)	109.67(12)
C(47)-C(45)-C(46)	109.24(12)	C(52)-C(60)-C(62)	113.19(12)
C(50)-C(48)-C(44)	112.70(13)	C(52)-C(60)-C(61)	110.61(12)
C(50)-C(48)-C(49)	109.84(13)	C(62)-C(60)-C(61)	108.21(11)
C(44)-C(48)-C(49)	110.48(13)		

Computational Details

Gas-phase calculations were performed with the use of Gaussian16 software[8] at the B3LYP/6-31G(d) level of theory, which offers a balance between accuracy and computational cost. Coordinates were taken from crystallographic data and were kept frozen for further calculations. Such obtained wave function was further used to perform QTAIM partitioning of electron density with the use of AIMAll software.[9] Natural bonding analysis of compound **1** was performed with the natural bond orbital[10] (NBO 3.1) partitioning scheme implemented in the Gaussian16. Natural charges and Wiberg bond indexes[11] (WBI) were obtained directly from NBO analysis.

To calculate the free energy changes associated with the proposed mechanism for the synthetic route leading to compound **2**, initial geometry optimizations were carried out at the HF/6-31G(d) level in the presence of toluene, modeled using the Polarizable Continuum Model (PCM), to generate starting geometries for subsequent refinements including electron correlation, performed using the hybrid density functional B3LYP with the same basis set. All calculations were performed using the Gaussian 16 software.

Despite extensive efforts to optimize the structures in the presence of toluene using the PCM method, larger compounds, namely **1**, **6**, **7**, and **I**, exhibited SCF convergence issues at certain stage of the minimization process, even when employing more computationally demanding approaches such as the quadratically convergent (QC) method, which combines linear searches far from convergence with Newton-Raphson steps near convergence (unless an energy increase is detected). The extended QC (XQC) method, which adds further robustness to the QC approach, was also attempted but did not lead to successful convergence. For this reason, these specific structures were initially optimized in the gas phase, i.e. removing the solvent model, resulting in minima without imaginary frequencies. Subsequent single-point PCM calculations were then performed to obtain free energies in the solvent environment. Minimization of all other compounds successfully converged in the presence of solvent without any imaginary frequencies.

It is worth noting that, when PCM was used, compounds **1** and **I** exhibited a single imaginary frequency, associated with motions involving groups around the Al–Al bond. Compounds **6** and **7** showed two imaginary modes each; however, due to the symmetry of these molecules, the modes correspond to equivalent atomic displacements on opposite halves. In compound **6**, the modes are related to the rotation of methyl groups, while in compound **7**, they result from the displacement of methyl hydrogen atoms toward the aluminum centers. The appearance of one or two imaginary frequencies is expected, even though the structures correspond to minima in the gas phase, as this can result from the change in environment when transitioning

to the PCM solvent model. Nonetheless, the computed energies remain reliable and offer a meaningful representation of the relative energetics, owing to the isotropic nature of the PCM environment. Additionally, calculations for all compounds were carried out in the singlet spin state, except for compound **7**, where the singlet state resulted in dissociation of the structure. Therefore, the dimer was computed in the triplet spin state.

Table S8: QTAIM topological properties of the electron density at bond critical points of Al−Al, Al−N and N−N chemical bonding. Electron density $\rho(\boldsymbol{r})$ and Laplacian of electron density $\boldsymbol{\nabla^2\rho}(\mathbf{r}) = \boldsymbol{\lambda}_1 + \boldsymbol{\lambda}_2 + \boldsymbol{\lambda}_3$ are given in atomic units and refer to e bohr^{-3} and e bohr^{-5}, respectively. Ellipticity ε is dimensionless and calculates as $(\boldsymbol{\lambda}_1/\boldsymbol{\lambda_2}) - \mathbf{1}$.

BCP	$\boldsymbol{\rho}(\mathbf{r})$	$\boldsymbol{\nabla^2\rho}(\mathbf{r})$	$\boldsymbol{\lambda_1}$	$\boldsymbol{\lambda_2}$	$\boldsymbol{\lambda_3}$	ε
Al2–N8	0.072	0.47	-0.11	-0.10	0.68	0.033
Al1–N5	0.071	0.47	-0.11	-0.10	0.69	0.038
Al2–NNA	0.060	-0.05	-0.05	-0.05	0.04	
Al1–NNA	0.060	-0.06	-0.05	-0.05	0.04	
N8–N9	0.465	-1.01	-0.88	-0.86	0.73	0.021
N9–N10	0.553	-1.52	-1.07	-1.07	0.62	0.008
N5–N6	0.473	-1.05	-0.90	-0.89	0.73	0.017
N6–N7	0.541	-1.43	-1.04	-1.04	0.64	0.006

Table S9: Wiberg bond indices (WBIs) of chosen bonds in complex **1**.

Bond	WBI	Bond	WBI
Al1–Al2	0.91		
Al1–N1	0.30	Al2–N3	0.30
Al1–N2	0.31	Al2–N4	0.30
Al1–N5	0.44	Al2–N8	0.45
N5–N6	1.63	N8–N9	1.62
N6–N7	2.24	N9–N10	2.25

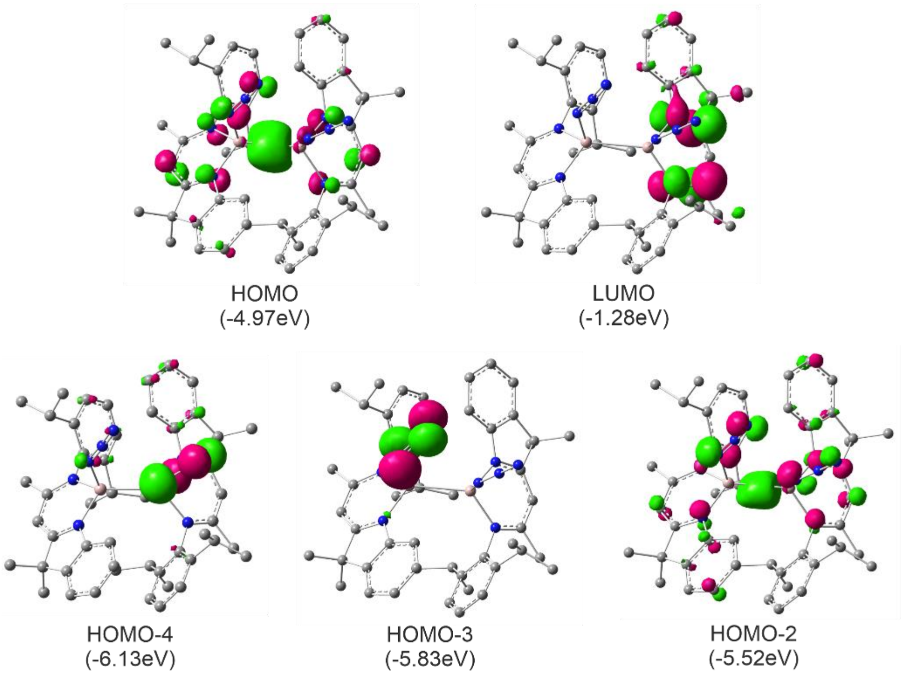

Figure S35: Chosen molecular orbitals in complex **1**. Surfaces are drawn at an isovalue of 0.05 au.

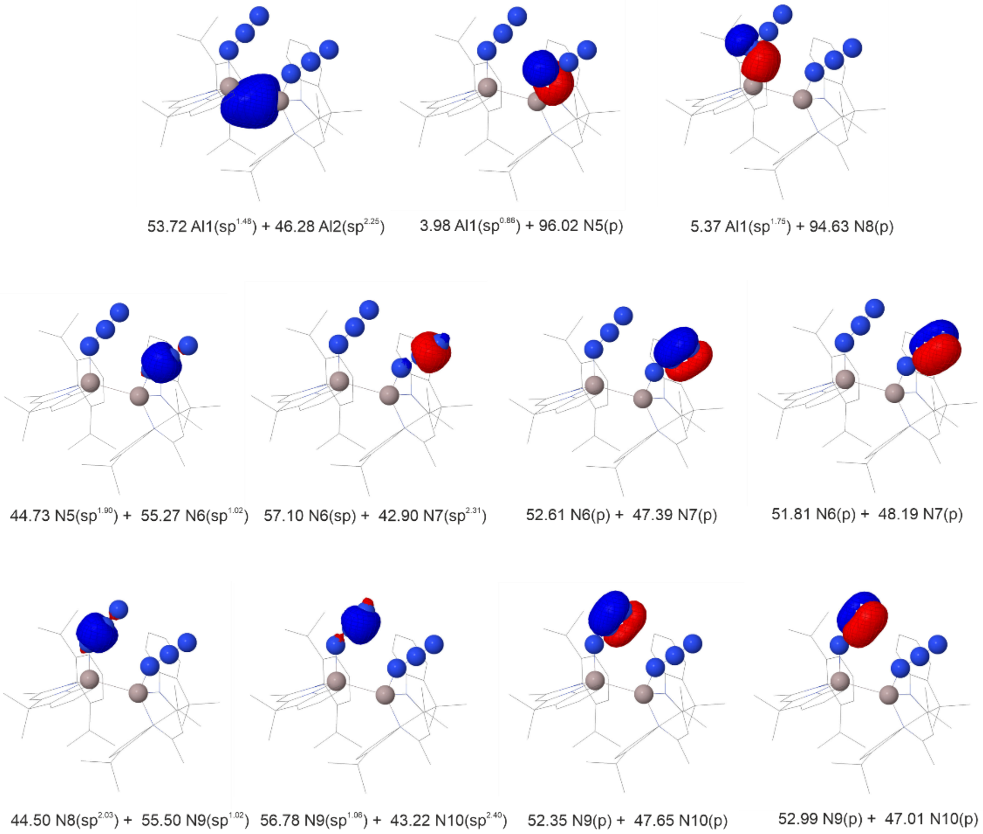

Figure S36: Natural bonding orbitals for Al−Al, Al−N and N−N chemical bonds observed in complex **1**.

Table S10: NBO details of the Al−Al, Al−N and N−N bonding in complex **1**; natural charges of the atoms are denoted as q_A and q_B.

A–B	occupancy	A contribution %	B contribution %	q_A	q_B
Al1–Al2	BD(1) 1.78	53.72 ($sp^{1.48}$)	46.28 ($sp^{2.25}$)	1.31	1.32
Al1–N5	BD(1) 1.60	3.98 ($sp^{0.86}$)	96.02 (p)	1.31	-0.76
Al2–N8	BD(1) 1.62	5.37 ($sp^{1.75}$)	94.63 (p)	1.32	-0.76
N5–N6	BD(1) 1.99	44.73 ($sp^{1.90}$)	55.27 ($sp^{1.02}$)	-0.76	0.23
	BD(1) 1.99	57.10 (sp)	42.90($sp^{2.31}$)		
N6–N7	BD(2) 1.99	52.61 (p)	47.39 (p)	0.23	-0.18
	BD(3) 1.99	51.81 (p)	48.19(p)		
N8–N9	BD(1) 1.99	44.50 ($sp^{2.03}$)	55.50($sp^{1.02}$)	-0.76	0.23
	BD(1) 1.99	56.78 ($sp^{1.06}$)	43.22 ($sp^{2.40}$)		
N9–N10	BD(2) 1.99	52.35 (p)	47.65 (p)	0.23	-0.17
	BD(3) 1.98	52.99 (p)	47.01 (p)		

Reference

[1] Bruker AXS Inc., *SAINT v8.40B*, Madison, WI, USA, **2019**.

[2] L. Krause, R. Herbst-Irmer, G. M. Sheldrick, D. Stalke, *J. Appl. Crystallogr.* **2015**, *48*, 3.

[3] M. Sevvana, M. Ruf, I. Usón, G. M. Sheldrick, R. Herbst-Irmer, *Acta Crystallogr. D* **2019**, *75*, 1040.

[4] G. M. Sheldrick, *Acta Crystallogr. A* **2015**, *71*, 3.

[5] G. M. Sheldrick, *Acta Crystallogr. C* **2015**, *71*, 3.

[6] C. B. Hübschle, G. M. Sheldrick, B. Dittrich, *J. Appl. Crystallogr.* **2011**, *44*, 1281.

[7] A. L. Spek, *Acta Crystallogr. C* **2015**, *71*, 9.

[8] M. J. Frisch, G. W. Trucks, H. B. Schlegel, G. E. Scuseria, M. A. Robb, J. R. Cheeseman, G. Scalmani, V. Barone, G. A. Petersson, H. Nakatsuji, X. Li, M. Caricato, A. V. Marenich, J. Bloino, B. G. Janesko, R. Gomperts, B. Mennucci, H. P. Hratchian, J. V. Ortiz, A. F. Izmaylov, J. L. Sonnenberg, D. Williams-Young, F. Ding, F. Lipparini, F. Egidi, J. Goings, B. Peng, A. Petrone, T. Henderson, D. Ranasinghe, V. G. Zakrzewski, J. Gao, N. Rega, G. Zheng, W. Liang, M. Hada, M. Ehara, K. Toyota, R. Fukuda, J. Hasegawa, M. Ishida, T. Nakajima, Y. Honda, O. Kitao, H. Nakai, T. Vreven, K. Throssell, J. A. Montgomery, Jr., J. E. Peralta, F. Ogliaro, M. J. Bearpark, J. J. Heyd, E. N. Brothers, K. N. Kudin, V. N. Staroverov, T. A. Keith, R. Kobayashi, J. Normand, K. Raghavachari, A. P. Rendell, J. C. Burant, S. S. Iyengar, J. Tomasi, M. Cossi, J. M. Millam, M. Klene, C. Adamo, R. Cammi, J. W. Ochterski, R. L. Martin, K. Morokuma, O. Farkas, J. B. Foresman, and D. J. Fox, *Gaussian16*, Gaussian, Inc., Wallingford CT, **2016**.

[9] *AIMAll (Version 19.10.12)*, Todd A. Keith, Overland Park KS, USA, **2019 (aim.tkgristmill.com)**.

[10] E. D. Glendening, A. E. Reed, J. E. Carpenter, and F. Weinhold., *NBO Version 3.1: natural bond orbital analysis program*, **2001**.

[11] K. B. Wiberg, *Tetrahedron* **1968**, *24*, 1083.
